# What works to improve early grade literacy in Latin America and the Caribbean? A systematic review and meta‐analysis

**DOI:** 10.1002/cl2.1067

**Published:** 2019-12-30

**Authors:** Rebecca Stone, Thomas de Hoop, Andrea Coombes, Pooja Nakamura

**Affiliations:** ^1^ American Institutes for Research Washington District of Columbia

## 1 PLAIN LANGUAGE SUMMARY

### 1.1 Early grade literacy (EGL) interventions in Latin America and the Caribbean (LAC) are only effective under certain conditions

Children across the world are not acquiring basic reading and math skills despite increases in primary school enrollment and attendance. Teacher training and nutrition programs in LAC are not effective in improving EGL overall, but they may be, under certain conditions. Technology in schools can be detrimental to learning outcomes if these programs only focus on technology.

### 1.2 What is this review about?

Approximately 250 million children across the world are not acquiring basic reading and math skills, even though about 50% of them have spent at least 4 years in school. Educational policies on EGL in the LAC region have long suffered from a disjuncture between school practice and research.

This review examines the effectiveness and fidelity of implementation of various programs implemented in the LAC region that aim to improve EGL outcomes, including teacher training, school feeding, computer‐aided instruction, nutrition, and technology‐in‐education.

What is the aim of this review?

This Campbell systematic review summarizes findings from 107 studies to inform policy for EGL in the LAC region.

### 1.3 What studies are included?

This review includes four types of EGL studies from the LAC region:

(1) Quantitative interventions (23 studies)
(2) Qualitative interventions (6 studies)
(3) Quantitative noninterventions (61 studies)
(4) Qualitative noninterventions (14 studies).

### 1.4 What are the main findings of this review?

Overall, programs did not have statistically significant effects on EGL outcomes. But there are instances in which programs may have positive or negative effects.

For example, teacher training did not show positive effects on EGL outcomes, but a study from Chile showed that teacher training can possibly positively affect EGL outcomes in high‐income economies when it is well implemented and complemented by sustained coaching. Similarly, nutrition programs did not improve EGL outcomes. However, a study from Guatemala showed positive effects on EGL, possibly because Guatemala has high rates of stunting and wasting.

Although there is no statistically significant effect of technology‐in‐education programs on EGL outcomes in the LAC region, a study from Peru showed that the distribution of laptops to children can have adverse effects, particularly when not complemented by additional programs.

Other studies showed that phonemic awareness, phonics, fluency, and comprehension are associated with reading ability. Furthermore, poverty and child labor are negatively correlated with EGL outcomes. This finding supports the result that nutrition programs may be effective in settings with high rates of stunting and wasting.

Finally, the quality of preschool and promoting social learning are positively associated with EGL outcomes.

### 1.5 What do the findings of this review mean?

Teacher training, nutrition, and technology‐in‐education programs on average do not show positive effects on EGL outcomes in the LAC region. However, there are several factors that could potentially enable positive impacts. These include combining teacher training with coaching, targeting school feeding and other nutrition programs to low‐income countries with high rates of stunting and wasting, and combining technology‐in‐education programs with a strong focus on pedagogical practices.

The review also identifies some opportunities for improving the design and implementation of EGL programs. Studies support the need to teach phonological awareness (PA) skills early on, but caution is required considering the small evidence‐base in the LAC region. The evidence also supports investing in preschool quality through well‐implemented teacher training.

Finally, ministries of education in low‐income countries with high rates of stunting and wasting could consider investing in programs to improve the nutrition outcomes of students.

Caution is needed in interpreting these findings since the evidence base on what works to improve EGL outcomes in the LAC region is weak, with indications of publication bias.

### 1.6 How up‐to‐date is this review?

The review authors searched for studies published up to February 2016.

## 2 EXECUTIVE SUMMARY

### 2.1 Background

Improvements in students' learning achievement have lagged behind in low‐ and middle‐income countries despite significant progress in school enrollment numbers. Approximately 250 million children across the world are not acquiring basic reading and math skills, even though about 50% of them have spent at least 4 years in school (United Nations Educational, Scientific and Cultural Organization (UNESCO), 2014). The World Development Report (World Bank, 2018) presents evidence showing that learning is unlikely to improve unless the quality of each factor improves. The LAC region has experienced some positive trends in educational outcomes in the last decade, including improved EGL outcomes for third‐grade students in the majority of the countries. However, educational policies on EGL in the LAC region have long suffered from a disjuncture between school practice and research. As a result, policy makers, pedagogy and curriculum specialists, and other stakeholders in the region are unable to determine high‐quality research and what works in improving EGL outcomes. For this reason, they are unable to shape policy, practice, and programs in an evidence‐driven manner.

### 2.2 Objectives

This systematic review examines the effectiveness of various programs implemented in the LAC region that aim to improve EGL outcomes, including teacher training, school feeding, computer‐aided instruction, programs with an emphasis on nutrition, and technology in education programs. In addition, we assess the fidelity of implementation of programs that aim to improve EGL outcomes as well as the factors that predict EGL outcomes. Finally, we examine the experiences and perspectives of various stakeholders about EGL in the LAC region.

Specifically, this review addressed the following research questions:

1. What is the impact of reading programs, practices, policies, and products aimed at improving the reading skills of children from birth through Grade 3 on reading outcomes in the LAC region?
2. What factors predict the reading outcomes of children from birth through Grade 3?
3. What factors contribute to improving the reading outcomes of children from birth through Grade 3?

### 2.3 Search methods

We searched electronic databases, gray literature, relevant journals, and institutional websites, and we performed keyword hand searches and requested recommendations from key stakeholders. The search was conducted from July to August 2015 and we finalized the search in February 2016. In addition, we used novel computational approaches (specifically Wikilabeling) to maximize the comprehensiveness of the review.

### 2.4 Selection criteria

This review includes studies that are relevant for the literacy of children in early grades in the LAC region. This literature included both studies with an emphasis on education and studies with a focus on enabling factors that are linked to education programs or reading outcomes. For example, we included studies with a focus on nutrition that may indirectly influence reading outcomes. We developed a theory of change to identify these enabling factors.

To answer our research questions, we included four study types. The first types are experimental and multivariate nonexperimental studies that include a control or comparison group. We defined these studies as “quantitative intervention studies.” We included these studies to determine the impact of specific programs on EGL outcomes. The second study type consists of qualitatively oriented studies with a focus on interventions. These studies usually emphasize the process of program implementation or the experiences of beneficiaries about the performance of the program. We defined these studies as “qualitative intervention studies.” The third study type emphasizes the predictors of reading outcomes but does not focus on the effects of a specific program. We defined these studies as “quantitative nonintervention studies.” We included these studies to increase our understanding of intermediate outcomes and their ability to predict reading outcomes. Fourth, we included qualitative studies that discuss literacy in the LAC region but do not include an emphasis on a specific program. We defined these studies as “qualitative nonintervention studies.”

### 2.5 Data collection and analysis

We systematically coded information from the studies included in the review and critically appraised them. We conducted statistical meta‐analysis and sensitivity analysis using the data extracted from quantitative experimental and quasiexperimental studies. We also used narrative synthesis techniques to synthesize the findings from qualitative studies and studies that focused on predictors of literacy outcomes.

### 2.6 Results

We included 107 studies with a focus on EGL in the LAC region. Initial searches resulted in 9,696 articles. Following a manual review of the abstracts, we were left with a total of 164 studies that underwent full‐text review. During this phase, an additional 57 articles were removed as not relevant, resulting in 107 studies included in the final review.

The 107 included articles were comprised of quantitative intervention research, quantitative nonintervention research, qualitative intervention research, and qualitative nonintervention research. We included 23 articles with studies that were experimental or quasiexperimental with a focus on the effects of specific development programs on EGL outcomes. Three of these 23 articles (Cardoso‐Martins, Mesquita, & Ehri, 2011; Larraín, Strasser, & Lissi, 2012; Vivas, 1996) each covered two distinct studies bringing the number of quantitative intervention studies included to 26. We also included 61 quantitative studies that had an emphasis on EGL outcomes but did not emphasize a specific intervention, 14 qualitative studies without a focus on a specific intervention, and six qualitative studies that focused on a specific intervention. Most of the studies included in our review of evidence were published journal articles and came from either Mexico or South America; significantly fewer articles were from Central America and the Caribbean. Almost all articles were published in English or Spanish. More than 90% of the articles were focused on high‐ to upper‐middle‐income countries.

We only found few quantitative intervention studies with a low risk of bias. Of the 26 included studies, seven were rated as having a low risk of selection bias, five were rated as having a medium risk of selection bias, and eight were rated as having a high risk of selection bias. Furthermore, 11 studies were rated as having a low risk of performance bias, seven studies were rated as having a medium risk of performance bias, and eight studies were rated as having a high risk of performance bias. We rated 14 studies as having a low risk of outcome and analysis reporting bias, five studies as having a medium risk of outcome and analysis reporting bias, and seven studies as having a high risk of outcome analysis reporting bias. Finally, we rated 17 studies as having a low risk of other biases, eight studies as having a medium risk of other biases, and one study as having a high risk of other biases.

Meta‐analyses did not show the average and statistically significant effects of development programs on EGL outcomes, but a narrative synthesis of the limited number of high‐quality quantitative intervention studies did show some examples of development programs that may have positive effects on EGL outcomes in specific circumstances and contexts. For example, a meta‐analysis that focused on teacher training did not show positive effects on EGL outcomes (95% confidence interval [CI] = −0.17, 0.48; evidence from two programs). However, a study from Chile showed that teacher training programs can possibly positively affect EGL outcomes in high‐income economies when they are well implemented and complemented by the sustained coaching of teachers (Pallante & Kim, 2013). In addition, a meta‐analysis that focused on nutrition programs did not show positive effects on EGL outcomes (95% CI = −0.08, 0.25; evidence from two programs). However, a study from Guatemala showed some evidence that nutrition programs can have positive effects on EGL outcomes in contexts where stunting and wasting are high (Hoddinott et al., 2013). On average, we also did not find statistically significant effects of technology in education programs on EGL outcomes in the LAC region (SMD = −0.01, 95% CI = −0.13, 0.10; evidence from three studies). However, a study from Peru showed that the distribution of laptops to children can have adverse effects on EGL outcomes, particularly when the distribution of laptops is not complemented by additional programs (Cristia, Ibarrarán, Cueto, Santiago, & Severín, 2012).

The findings of the quantitative nonintervention studies indicate that phonemic awareness, phonics, fluency, and comprehension are associated with reading ability. The research also indicates that poverty and child labor are negatively correlated with EGL outcomes. This finding on the importance of poverty and socioeconomic factors for EGL outcomes supports the quantitative intervention result that nutrition programs may be effective in improving EGL outcomes in contexts with high rates of stunting and wasting. Finally, the quantitative nonintervention studies show that the quality of preschool is positively associated with EGL outcomes.

Both qualitative and quantitative studies indicated that consideration of context is key to improving reading outcomes. The most frequently discussed topic in qualitative nonintervention articles was the need to promote social learning to improve EGL.

We found some indications for publication bias in the studies that focus on the effects of teacher practices, parental involvement, and Information and Communication Technology (ICT) programs on EGL outcomes in the LAC region; that is, it is possible that some studies that focus on EGL in the LAC region were not published because they did not find statistically significant effects.

### 2.7 Authors' conclusions

Our review highlighted several important implications for practice and policy related to the rollout, design, and potential impact of education programs that aim to improve EGL outcomes in the LAC region. First, our quantitative evidence suggests that teacher training, nutrition, and technology in education programs on average do not show positive effects on EGL outcomes in the LAC region. However, the quantitative narrative synthesis shows several factors that could potentially enable positive impacts of these programs on EGL outcomes. These factors include combining teacher training with teacher coaching, targeting school feeding and other nutrition programs to low‐income countries with high rates of stunting and wasting, and combining technology in education programs with a strong focus on pedagogical practices. However, the evidence‐base is too small to derive strong conclusions about the ability of these components to improve EGL outcomes in the LAC region.

Second, the systematic review identified some promising opportunities for improving the design and implementation of education programs that aim to improve EGL outcomes. We found evidence for a strong correlation between PA and reading ability. In addition, studies focused on the importance of PA and phonics to help students become strong decoders. These findings suggest the need to teach PA skills early on, but caution is required considering the small evidence‐base in the LAC region.

Third, the review suggests that more resources may potentially need to be focused on enhancing the quality of preschools through well‐implemented teacher training. The findings of this review suggest that such teacher training could possibly enhance reading outcomes if the training is complemented with sustained teacher coaching. The evidence‐base is, however, again too small to derive strong conclusions about the effects of teacher training in preschools.

Fourth, ministries of education in low‐income countries with high rates of stunting and wasting could consider potentially investing in programs to improve the nutrition outcomes of students in order to improve EGL outcomes. These efforts may be less effective in middle‐ or high‐income countries, however, and more evidence is needed to derive strong conclusions about the effects of programs that aim to improve nutrition on EGL outcomes.

In general, the evidence base on what works to improve EGL outcomes in the LAC region is weak. We only found a small number of studies that can present credible estimates on the impact of development programs on EGL outcomes. The majority of the studies suffer from either a medium or high risk of selection bias or a medium or high risk of performance bias. Furthermore, we found some indications for publication bias in the studies that focus on the effects of teacher practices and parental involvement on EGL outcomes in the LAC region.

## 3 BACKGROUND

### 3.1 The problem, condition, or issue

There is evidence of a global learning crisis (Berry, Barnett, & Hinton, 2015; Nakamura, de Hoop, & Holla, 2019; Pritchett & Sandefur, 2013). School enrollment has improved, but EGL and math assessment data have shown high “zero” scores in literacy assessments in many low‐ and middle‐income countries (e.g., Annual Status of Education Report [ASER], 2013; EGRA data, n.d.).

The findings of the latest World Development Report on education highlight how educational outcomes are affected directly by the quality of school inputs, school management, and teachers, as well as the education preparedness of learners. In theory, improvements in the quality of one of these factors could result in improvements in learning outcomes. However, the World Development Report (World Bank, 2018) presents evidence demonstrating that learning is unlikely to improve unless the quality of each factor improves. A systematic review of Snilstveit et al. (2012) also argues that education programs are unlikely to improve learning outcomes unless they ease more than one constraint.

**Figure 1:**
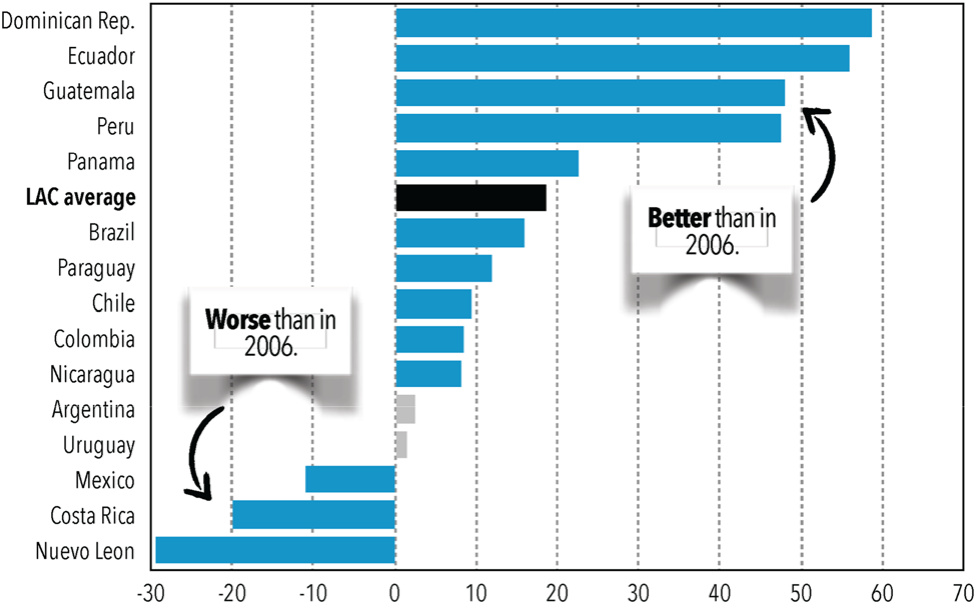
Change in mean scores in third‐grade reading, 2006–2013.(1) Only changes shown in blue or black are statistically significant. (2) The mean score for the region includes all countries in this graph with equal weight *Source:* from Are Latin American children's reading skills improving? Highlights of the second and third regional comparative and explanatory studies (SERCE & TERCE). Washington, DC: American Institutes for Research; p. 15. Reprinted with permission

The LAC region is composed of more than 40 countries and territories on two continents with five different official languages (English, Spanish, French, Dutch, and Portuguese) and many more regional languages. The region has experienced some positive trends in educational outcomes in the last decade, including improvements in pupil/teacher ratios, increases in the percentage of trained teachers (UNESCO, 2014, p. 8), and improved EGL outcomes for third‐grade students in the majority of the countries (see Figure 1).

**Figure 2:**
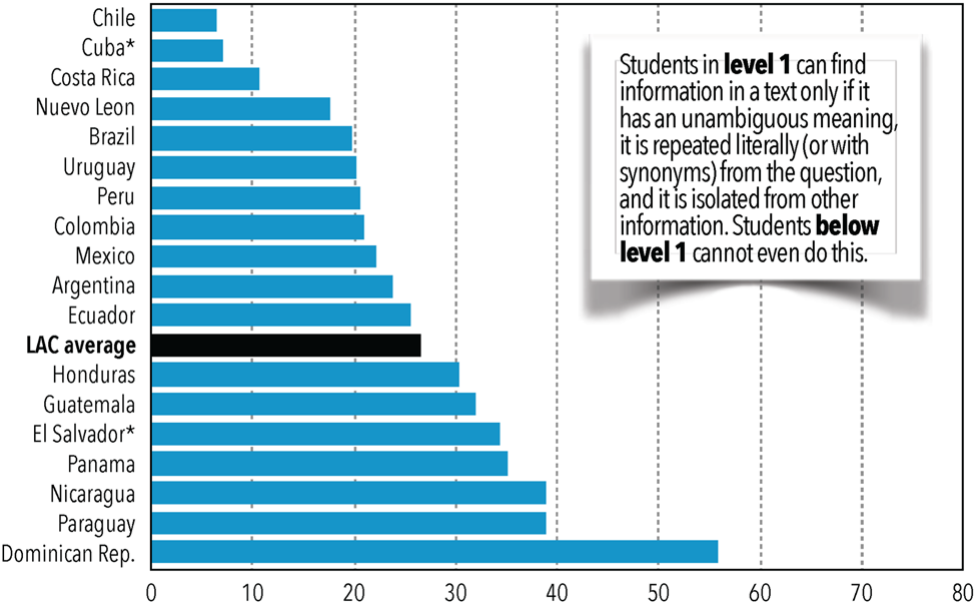
Percentage of third graders scoring at level 1 or below on reading, 2013.(1) Lowest levels include level 1 and below. (2) The mean score for the region includes all countries except for Cuba, El Salvador, and Honduras with equal weights. (3) Cuba's and El Salvador's scores are from 2006 *Source:* from Are Latin American children's reading skills improving? Highlights of the second and third regional comparative and explanatory studies (SERCE & TERCE). Washington, DC: American Institutes for Research; p. 19. Reprinted with permission

However, we still find great disparities among the poor, rural, indigenous, and other disadvantaged groups in the region. In addition, one in four third graders performed so poorly that they were categorized in the lowest level of the reading test, and <5% of the third graders performed so well that they were categorized as achieving the highest levels of reading. Figure 2 depicts these challenges by demonstrating that there are still a significant number of third graders scoring at the lowest levels of reading. In fact, more than 60% of third‐grade students have only achieved basic reading skills (Levels 1 and 2).

There are many reasons to explain the poor literacy outcomes in the region but one of the key potential reasons is the lack of evidence‐based training, preparation and support for teachers. According to Bruns and Luque (2015) “the seven million teachers of LAC are the critical actors in the region's efforts to improve education quality and raise student learning levels, which lag far behind those of OECD countries and East Asian countries such as China.” Some of the reasons they cite are the low standards for entry into teacher training, poor quality training programs that are removed from the realities of the classroom, few career incentives, and weak support for teachers once they are on the job. In addition, teachers are not receiving the training they need to deal with students at very different learning levels, different ages, speaking different languages, and so forth, which is the reality of most LAC classrooms.

Evidence‐informed EGL policy can contribute to mitigating some of the concerns associated with EGL outcomes in the LAC region. However, up until now, education policies to improve reading outcomes have only been informed by evidence to a limited extent.

### 3.2 The interventions

National governments and development agencies in the LAC region have created a range of programs to improve EGL outcomes. Some of these programs specifically aim to improve EGL outcomes while others might improve EGL through indirect mechanisms. This review aimed to include any program that had the potential to affect EGL outcomes. We found and included research on the following program types and practices: teacher training, technology in education programs, school feeding and other nutrition programs, school governance programs, preschool programs, teacher practices and general pedagogical approaches, parental and community participation, and curricula. We discuss each of these intervention types below.

Teacher training programs can take several forms ranging from extensive multiyear, one‐on‐one coaching delivered to teachers in their classrooms to training workshops delivered outside of the classroom. Emerging evidence suggests that teacher training models that emphasize sustained in‐class coaching may produce larger effects on learning outcomes than short‐term training models in developing countries (Kraft, Blazar, & Hogan, 2018). For instance, a study from South Africa showed that monthly visits from specialized training coaches resulted in statistically significant effects on reading outcomes (0.25 standard deviations), while two 2‐day training sessions (provided over the course of a year) did not result in statistically significant effects on reading outcomes (Cilliers & Taylor, 2017).

Technology in education programs involve providing technological equipment (e.g., laptops, digital game‐based technology, mobile phones) to teachers or learners and integrating these tools into existing curriculums or including technology as additional tools. The equipment may have been refurbished and donated by the private sector or produced specifically for classroom instruction (Barrera‐Osorio & Linden, 2009; Cristia et al., 2012). Some programs may complement the distribution of technological equipment with training modules for teachers on the use of technology in the classroom for specific subjects. Other programs do not provide any complementary training. Studies that have examined the impact of technology in education programs on learning outcomes in low‐ and middle‐income countries suggest mixed evidence with a pattern of null results for programs that do not focus on complementary training for teachers (Bulman & Fairlie, 2016, p. 2). However, recent evidence shows more promising results for programs that include a strong focus on teaching at the right level. For example, Muralidharan et al. (2019) showed that a technology‐based afterschool instruction program with a strong emphasis on learning at the right level produced large and statistically significant effects on reading outcomes in India.

School feeding and nutrition programs vary in their modes of delivery and expected outcomes. Most programs are delivered within schools and provide meals (typically breakfast or lunch) to participating children (Adrogue & Orlicki, 2013; Powell, Walker, Chang, & Grantham‐McGregor, 1998). Other programs may provide children with specific nutrients that might be missing from their diets (Maluccio et al., 2009). Nutrition programs may aim to improve school attendance and boost learning outcomes, in addition to aiming to aiming to improve children's food security and nutritional status.

School governance interventions address school management issues that affect the delivery, quality, and financing of education. These programs often focus on decentralizing decision‐making at the school level or improve parents' and communities' involvement in school management. Some school governance interventions involve cash transfers to schools and provision of matching funds for private investment to schools along with institutional changes, which allows parents to decide how to allocate funds (Bando, 2010). Other models might provide support to poor performing schools based on needs identified in a school improvement plan (Lockheed, Harris, & Jayasundera, 2010).

Early childhood education programs often focus on preschool before the start of primary education. The effects of preschool can be moderated by variations in the length of time spent in preschool, availability and quality of school resources, quality of instruction and extraschool factors such as household income (Gardinal‐Pizato, Marturano, & Fontaine, 2012).

Interventions aimed at supporting parents in fostering children's early literacy take varied approaches and have shown mixed results. In developed countries, several interventions focus on addressing parent tutoring to improve children's literacy (Hannon, 1995; Tizard, Schofield, & Hewison, 1982; Topping, 1995). Several reviews have summarized the findings from literacy training programs for parents (Brooks, 2002; National Literacy Trust, 2001), but the effectiveness of parent training on children's literacy has not been established through systematic reviews, largely because of methodological discrepancies among the studies (Sylva, Scott, Totsika, Ereky‐Stevens, & Crook, 2008).

Interventions that target curriculum and teacher practices for literacy instruction take varied approaches as well. For instance, some interventions encourage teachers to explain unknown words to learners during storybook reading in order to boost reading comprehension (Larraín et al., 2012). Other interventions focus on providing PA training to boost learners' letter sound recognition (Cardoso‐Martins et al., 2011). Curricular interventions involve more actors and may have systemwide outcomes. For instance, interventions may focus on the reform of an existing curriculum to integrate content across subject areas or implement teaching strategies that cater to different cognitive levels (Roofe, 2014).

### 3.3 How the intervention might work

**Figure 3:**
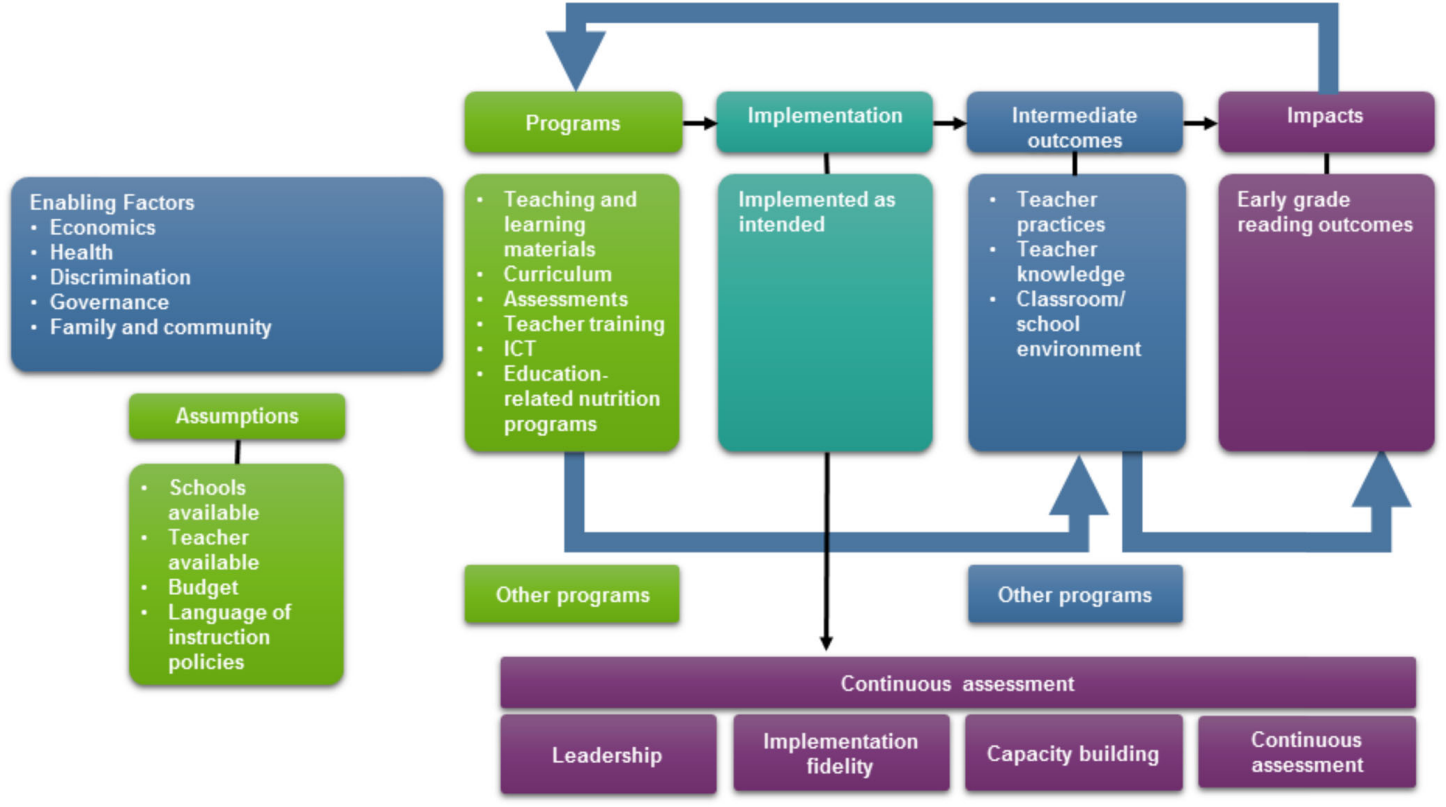
Theory of change

We developed a generic theory of change that—for all types of programs described above—maps out the plausible linkages across enabling factors, education‐ and noneducation‐related programs or initiatives that are associated with literacy, intermediate outcomes, and reading outcomes, as well as the assumptions that underlie the theory of change. The theory of change explains how programs or initiatives can contribute to improving EGL outcomes in a sustainable manner. Figure 3 depicts the theory of change.

The theory of change begins with the enabling factors and assumptions that are necessary for any intervention or program to be able to impact EGL outcomes in the LAC region. These factors refer to assumptions that need to be in place to enable successful programs that are effective in improving reading outcomes. Then, education programs are implemented along with other noneducation programs that may have complementary, indirect, or moderating effects on EGL. Successful implementation then enables the achievement of intermediate EGL outcomes, such as changes in teacher knowledge and practices, which can, in turn, improve EGL. Finally, we include key elements for sustainability—namely, leadership, implementation fidelity, capacity building, and continuous assessment—that enable implementation to continue producing outcomes and impacts. Sustainability also depends on overcoming potential barriers, including financing, motivation at the community level, turnover in the government, and prioritization of these goals among competing initiatives.

The theory of change also considers mechanisms that influence how stakeholders interact with programs or practices, as well as external or contextual factors that influence implementation and the linkages in the conceptual framework. Importantly, the linkages in the conceptual framework can be moderated by the enabling environment. This enabling environment consists of the institutions and other contextual characteristics that need to be in place to enable the implementation of successful programs that are effective in improving EGL outcomes. For example, teacher training programs are likely to be more effective in an environment with a sufficient number of qualified teachers with the incentive to attend school. Similarly, teaching students how to read is likely to be more effective in an environment in which students are not stunted or wasted. Finally, a strong governance structure sets the stage for high‐quality education by ensuring that schools and teachers are available and have a budget within which they can implement programs or practices.

### 3.4 Why it is important to do the review

The World Conference on Education for All held in 1990 expanded the focus of the education agenda from access to quality and brought a new interest in the quality of education students received (World Conference on Education for All, 1990). Two of the six goals adopted at the Jomtien conference led to greater interest and support for EGL development. They were Goal 1: the expansion of early childhood care and development activities; and Goal 3, improvement in learning achievement.

There is evidence that programs in low‐ and middle‐income countries that focus on increasing educational inputs without addressing other constraints to learning are not sufficient to improve learning outcomes (Snilstveit et al., 2012). Banerjee et al. (2007) note that increasing inputs fail to have an impact on student attainment if what is being taught remains too difficult for students to learn. Similarly, a number of studies focused on computer‐assisted learning programs did not find significant impacts. For example, Cristia et al. (2012) analyzed the effect of the One‐Laptop‐Per‐Child program for students in rural Peru; they found little impact on the attendance and educational attainment of students. They argue that this lack of impact is due to the computers not containing software directly linked to class material, such as mathematics or reading, as well not having clear instruction on how teachers should use the computers in class.

This evidence shows the importance of identifying programs that are effective in improving learning outcomes. Recent systematic reviews show that structured pedagogical interventions targeted at teaching the right skills are among the most effective education interventions to improve learning outcomes in low‐ and middle‐income countries, particularly when the structured pedagogical intervention primarily focuses on teaching in the mother tongue (Evans & Popova, 2015; Snilstveit et al., 2012). However, it is important to develop context‐specific solutions for the LAC region. This systematic review aims to do so by providing specific evidence on what works to improve EGL outcomes in this region.

Educational policy around EGL in the LAC has long suffered from a disjuncture between school practice and research. Systematic reviews exist on the effects of education programs on learning outcomes (Evans & Popova, 2015; Snilstveit et al., 2012) and the impact of parental, community, and familial support interventions to improve children's literacy (Spier et al., 2016), but it is unclear whether these global findings can be extrapolated to the LAC region. Also, within the LAC region itself, research on EGL is fragmented and often of poor quality. There is no comprehensive or systematic overview of the EGL research literature specific to the LAC region. As a result, policy makers, pedagogy and curriculum specialists, and other stakeholders in the region are unable to determine what is relevant and are thus unable to shape policy, practice, and programs in an evidence‐driven manner.

Critical gaps in the literature and challenges in the achievement of EGL outcomes remain inside the LAC region. Most of the existing evidence on EGL is from outside the LAC region, and it is unclear whether these findings can be extrapolated to the LAC region. In addition, most of the evidence on EGL, both inside and outside the LAC region, is based on correlations and does not allow for causal claims about the impact of education and noneducation programs on EGL outcomes. These factors limit the possibility of evidence‐informed policy making.

This study will be the first systematic review to assess the evidence on EGL specifically from the LAC region. The review will also provide evidence on additional factors that support early literacy development outside of programs. This information could help to improve the design of early literacy programs at home, in schools and with parents and communities. Policy makers and practitioners need guidance in order to make use of evidence that is voluminous, diverse, and fragmented across disciplines. For research to be relevant to policy, it must be captured and consolidated in a reliable and accessible manner. It is important to differentiate research results on the basis of the quality of the methodology so that policy makers can make decisions that are based on valid findings. To that end, we reviewed and appraised the quality of all of the different methodological approaches used by the evaluations.

## 4 OBJECTIVES

The objective of this systematic review is to synthesize the high‐quality quantitative and qualitative evidence on what works to improve EGL outcomes in LAC. To achieve this goal, we addressed the following research questions.

(1) What is the impact of reading programs, practices, policies, and products aimed at improving the reading skills of children from birth through Grade 3 on reading outcomes in the LAC region?
(2) What factors predict the reading outcomes of children from birth through Grade 3?
(3) What factors contribute to improving the reading outcomes of children from birth through Grade 3?

## 5 METHODS

### 5.1 Criteria for considering studies for this review

#### 5.1.1 Types of studies

To answer our research questions, we included four study types. The first types are experimental and multivariate nonexperimental studies that include a control or comparison group. We defined these studies as “quantitative intervention studies.” We included these studies to determine the impact of specific programs on EGL outcomes. The second study type consists of qualitatively oriented studies with a focus on interventions. These studies usually emphasize the process of program implementation or experiences of beneficiaries about the performance of the program. We defined these studies as “qualitative intervention studies.” The third type of study emphasizes predictors of reading outcomes and does not focus on the effects of a specific program. We defined these studies as “quantitative nonintervention studies.” We included these studies to increase our understanding of intermediate outcomes and their ability to predict reading outcomes. Fourth, we included qualitative studies that discuss literacy in the LAC region but do not include an emphasis on a specific program. We defined these studies as “qualitative nonintervention studies.” We included these studies to assess the experiences and perspectives of key stakeholders, including students, teachers, and policy makers, concerning literacy and reading.

##### 5.1.1.1 Experimental and quasiexperimental studies

We relied on quantitative experimental or quasiexperimental studies to address research question 1. We included both randomized controlled trials (RCTs) and quasiexperimental designs with nonrandom assignment. We include multivariate nonexperimental designs such as regression discontinuity designs, “natural experiments,” and studies in which students or schools self‐select into the program. To be included, the studies needed to collect cross‐sectional or longitudinal data for both beneficiaries and control or comparison groups and use propensity score or other types of matching, difference‐in‐difference estimation, instrumental variables regression, multivariate cross‐sectional or longitudinal regression analysis, or other forms of multivariate analysis, such as the Heckman selection model. The studies did not necessarily have to demonstrate baseline equivalence to be included in the review.

##### 5.1.1.2 Qualitative studies on interventions

The second study type consists of qualitatively oriented studies with a focus on interventions that aim to improve EGL outcomes (either directly or indirectly). These studies usually emphasize the process of program implementation or experiences of beneficiaries about the performance of the program. We defined these studies as “qualitative intervention studies.” We included these studies to assess the experiences and perspectives of key stakeholders, including students, teachers, and policy makers, concerning literacy and reading.

##### 5.1.1.3 Qualitative nonintervention studies

We also included qualitative studies that discuss literacy in the LAC region, but do not include an emphasis on a specific program. We defined these studies as “qualitative nonintervention studies.” We included these studies to assess the experiences and perspectives of key stakeholders, including students, teachers, and policy makers, concerning literacy and reading.

##### 5.1.1.4 Quantitative studies that focus on predictors of reading outcomes

The fourth type of study emphasizes predictors of reading outcomes and does not focus on the effects of a specific program. We defined these studies as “quantitative nonintervention studies.” We included these studies to increase our understanding of intermediate outcomes and their ability to predict reading outcomes.

#### 5.1.2 Types of participants

We included studies that focused on programs that included children in early grades in LAC from birth through grade 3. This time period was selected as it aligns with the funder's (USAID) definition of EGL. In cases where effects were reported for children in early grades and higher grades in LAC, studies were eligible for inclusion if a subgroup of the beneficiaries were children in early grades in LAC from birth through grade 3. Studies were also eligible for inclusion if they included children who were in grade 4 or higher during the endline survey but were in early grades (from birth through grade 3) during the start of the program.

#### 5.1.3 Types of interventions included

The interventions included in this review were programs that aimed to improve EGL outcomes directly or could improve EGL outcomes through indirect mechanisms. We did not exclude studies that focused on programs that did not explicitly aim to improve EGL outcomes.

#### 5.1.4 Types of interventions excluded

We excluded studies that focused on interventions that could influence reading but that did not discuss the link between the intervention and reading outcomes specifically (e.g., studies with a focus on improving IQ).

#### 5.1.5 Types of outcome measures

To address Research Questions 1 and 2, we included studies that focused on EGL outcomes. To be included, studies needed to assess either EGL outcomes or EGL practices.

*EGL outcomes:* We included all studies that focused on a range of measures of EGL, including assessment tests and self‐reported measures of EGL.

*EGL practices:* We included all studies that focused on a range of measures of EGL practices, including measures of the time children spent on reading books.

We did not define outcome criteria to address Research Question 3, because studies included to address this research question were qualitative studies.

#### 5.1.6 Language

We searched for studies published in any language that would have been relevant to the LAC region, including but not limited to English, Spanish, Portuguese, French, and Dutch. We did not exclude any studies based on language.

#### 5.1.7 Types of settings

We included studies from all countries in the LAC region, as defined by the World Bank. We included any studies we found from or about the following countries:

Antigua and Barbuda, Argentina, Aruba, Bahamas, Barbados, Belize, Bermuda, Bolivia, Brazil, British Virgin Islands, Cayman Islands, Chile, Colombia, Costa Rica, Cuba, Curacao, Dominica, Dominican Republic, Ecuador, El Salvador, French Guiana, Grenada, Guadeloupe, Guatemala, Guyana, Haiti, Honduras, Jamaica, Martinique, Mexico, Mont Serrat, Netherlands Antilles, Nicaragua, Panama, Paraguay, Peru, Puerto Rico, Saint Barthelemy, Saint Kitts and Nevis, Saint Lucia, Saint‐Martin, Saint Vincent and the Grenadines, Sint Maarten, Suriname, Trinidad and Tobago, Turks and Caicos Islands, Uruguay, U.S. Virgin Islands, Venezuela

### 5.2 Search methods for identification of studies

#### 5.2.1 Developing the search strategy

To develop and refine the search strategy, we relied on our PICO criteria and consultations with other researchers, librarians, computer scientists, and content experts. Through this process, we selected the most relevant databases for our review. The primary requirement for selected databases—ability to search the full database—is critical to ensure that the selection process was impartial. For example, Google Scholar is a source of unpublished or “grey” literature. However, it does not provide an interface that allows for a systematic search and retrieval of all potentially relevant documents. Rather, the query yields only the top results as defined by the Google search algorithm. After selecting appropriate databases, the team drafted, tested, and refined the initial search queries overall and by database specifications to identify the search string that best captured the most potentially relevant evidence for the population, topic, and time frame of interest.

The systematic review team constructed a database query by identifying search terms using the *population criteria* **.** To capture both quantitative studies for answering Research Question 1, qualitative intervention and nonintervention relevant to Research Question 3, and quantitative nonintervention research relevant to Research Question 2, we did not include search strings for study design, comparison condition, or outcome measures. Using these criteria in the search strategy would have excluded relevant qualitative studies, as well as quantitative and mixed‐methods studies that omitted this information from the title and abstract.

The terms below represent the keywords and phrases that were identified for our English search. Their equivalents in the other target languages are listed in Table 1.

- Population:
  - Birth to grade 3, 0–10, early childhood, preschool, preprimary, primary, kindergarten, grade 1, grade 2, grade 3, day care, early‐grade, elementary
  - Latin America, Caribbean, Central America, South America, Antigua and Barbuda, Argentina, Aruba, Bahamas, Barbados, Belize, Bermuda, Bolivia, Brazil, British Virgin Islands, Cayman Islands, Chile, Colombia, Costa Rica, Cuba, Curacao, Dominica, Dominican Republic, Ecuador, El Salvador, French Guiana, Grenada, Guadeloupe, Guatemala, Guyana, Haiti, Honduras, Jamaica, Martinique, Mexico, Mont Serrat, Netherlands Antilles, Nicaragua, Panama, Paraguay, Peru, Puerto Rico, Saint Barthelemy, Saint Kitts and Nevis, Saint Lucia, Saint‐Martin, Saint Vincent and the Grenadines, Sint Maarten, Suriname, Trinidad and Tobago, Turks and Caicos, Islands, Uruguay, U.S. Virgin Islands, Venezuela

**Table 1:** Search terms in English, Spanish, French, Portuguese, and Dutch

English
(Read* OR Litera* OR writ* OR communic*) AND (primary sch* OR primary grad* OR “grades 1 through 3” OR “grades 1 to 3” OR “grades 1–3” OR “first through third” OR “Grade 1” OR first grade* OR “grade 2” OR second grade* OR “grade 3” OR third grade* OR early grade* OR elementary OR kinder* OR pre‐school* OR preschool* OR prekindergarten* OR preK OR pre‐K OR “early childhood”) AND (Latin America* OR Caribbean OR South America* OR Antigua* and Barbuda OR Argentin* OR Aruba OR Bahama* OR Barbados OR Beliz* OR Bermud* OR Bolivia* OR Brazil* OR “British Virgin Islands” OR “Cayman Islands” OR Chile* OR Colombia* OR Costa Ric* OR Cuba* OR Curaca* OR Dominica* OR “Dominican Republic” OR Ecuador* OR El Salvador* OR French Guiana* OR Grenada* OR Guadeloup* OR Guatemala* OR Guyana* OR Haiti* OR Hondura* OR Jamaica* OR Martinique OR Mexic* OR Mont Serrat OR “Netherlands Antilles” OR Nicaragua* OR Panama* OR Paraguay* OR Peru* OR “Puerto Ric*” OR “Saint Barthelemy” OR “Saint Kitts and Nevis” OR Saint Lucia* OR “Saint‐Martin” OR “Saint Vincent and the Grenadines” OR “Sint Maarten” OR Surinam* OR “Trinidad and Tobago” OR “Turks and Caicos” OR Uruguay OR “Virgin Islands” OR Venezuela)

Spanish
(Leer OR Lecto‐escritura OR Alfabetiz* OR “Ambiente letrado”) AND (“la escuela primaria” OR “grados de primaria” OR “grados 1ero a 3ero” OR “grados 1 a 3” OR “grados 1–3” OR “de primer grado a tercer grado” OR “Grado 1” OR “primer grado” OR “primeros grados” OR “primer grado” OR “grado 2” OR “segundo grado” OR “grado 3” OR “tercer grado “OR “grados iniciales” OR “grados tempranos” OR “educación preescolar” OR “Educación maternal” OR “jardín de infancia” OR “Jardines de infancia” OR Kinder* OR preescolar OR pre‐kindergarten OR “primera infancia” OR “Educación Inicial”) AND (“Latino América” OR Caribe OR “Sud América” OR “América del Sur” OR “Antigua y Barbuda” OR Argentin* OR Arub* OR Baham* OR Barbados OR Belice* OR Bermud* OR Bolivi* OR Brasil OR “Islas Virgenes Birtánicas” OR “Gran Cayman” OR Chil* OR Colombi* OR “Costa Rica” OR Cub* OR Curaca* OR Dominica* OR “República Dominicana” OR Ecuador* OR “El Salvador” OR “Guayana Francesa” OR Grenada* OR Guadalupe OR Guatemal* OR Guyana* OR Guayana OR Haiti* OR Hondur* OR Jamaic* OR Martinic* OR Méxic* OR “Mont Serrat” OR “Antillas Holandesas” OR Nicaragu* OR Panamá* OR Paraguay* OR Perú* OR “Puerto Ric*” OR “San Bartolomé” OR “Saint Kitts y Nevis” OR “Saint Lucia” OR “Saint‐Martin” OR “Saint Vincente y Granadines” OR “San Martín” OR Surinam OR “Trinidad y Tobago” OR “Turks y Caicos” OR Uruguay OR “Islas Vírgenes” OR Venezuel*)

French
(lire OR “la lecture” OR l'écriture OR écrire OR “l'Alphabétisation” OR “environnement lettré” OR “lire‐écrire”) AND (“l'école primaire” OR “Enseignement primaire” OR “l'école élémentaire” OR “première année” OR “deuxième année de cycle 2” OR “cours préparatoire” OR “CP” OR “troisième année de cycle 2” OR “cours élémentaire1re année” OR “CE1” OR “première année du cycle 3” OR “cours élémentaire 2e année” OR “CE2” OR “maternelle” OR “Préscolaire” OR “petite enfance”) AND (“Amérique latine” OR Caraïbes OR “Amérique du Sud” OR “Antigua‐et‐Barbuda” OR Argentine OR Aruba OR Antilles OR Bahamas OR Barbade OR Belize OR Bermudes OR Bolivie OR Brésil OR “Îles Vierges britanniques” OR “Grand Cayman” OR Chili OR Colombie OR “Costa Rica” OR Cuba OR Curaçao OR Dominique OR “République dominicaine” OR Equateur OR “El Salvador” OR Guyane OR Grenade OR Guadeloupe OR Guatemala OR Haïti OR Honduras OR Jamaïque OR Martinique OR Mexique OR “Mont Serrat” OR Nicaragua OR Panama OR Paraguay OR Pérou OR “Puerto Rico” OR “San Bartolomé” OU “Saint Kitts‐et‐Nevis” OR “Saint Lucia” OR “Saint‐Martin” OR “Saint‐Vincent‐et‐Grenadines” OR Suriname OR “Trinité‐et‐Tobago” OR “îles Turks et Caicos” OR Uruguay OR Venezuela)

Portuguese
(Leitura OR Escrever OR Alfabetização OR “Alfabetização Inicial” OR “Alfabetização Infantil” OR “Alfabetização Emergente” OR “Alfabetização de Crianças” OR “Meio de Alfabetização” OR “Ambiente Escritura” OR “Compreensão de leitura” OR “Literatura Infantil” OR “tradições orais indígenas” OR “alfabetização inicial endógena na língua maternal”) AND (“Escola Primária *” OR “graus elementares” OR “graus primeiro‐terceiro” OR “graus 1–3” OR “graus 1–3” OR “primeiro grau para a terceira série” OR “Grau 1” OR “primeiro grau” OR “séries iniciais” OR “pré‐escolar” OR “jardim de infância” OR Creche OR Maternal OR Kinder OR pré‐escola OR pré‐jardim de infância* OR “primeira infância” OR “Educação da Primeira Infância”) AND (“America Latina” OR Caribe OR “América do Sul* OR “Antígua e Barbuda” OR Argentina OR Aruba, OR Bahamas OR Barbados OR Belize OR Bermuda OR Bolívia OR Ilhas Virgens OR Brasil OR Gran Cayman Británicas OR Chile* OR Colômbia* OR Costa Rica* OR Cuba, OR Curacao OR Dominicana* OR Equador OR “El Salvador” OR Grenada OR Guiana OR Guadalupe OR Guatemala* OR Haiti OR Honduras OR Jamaica OR Martinica OR México OR “Mont Serrat” OR “Antilhas Holandesas” OR Nicarágua OR Panamá* OR Paraguai* OR Peru* OR “Porto Rico” OR “São Bartolomeu” OR “São Cristóvão e Nevis” OR “Santa Lúcia” OR “São Martin” OR “São Vicente e Granadinas” OR Suriname OR “Trinidad e Tobago” OR “Turcas e Caicos” OR Uruguai OR Venezuela) AND (meninas OR meninos OR crianças* OR bebês OR infantil)

Dutch
(Lezen* OR Alfabetisering) AND (“basisschool*” OR “basisonderwijs*” OR “groep 3 tot en met 5” “groep 3 tot 5” OR “groep 3–5” OR “groep 3” OR “groep 4”OR “groep 5” OR kleuterschool* OR peuterspeelzaal* OR kinderopvang* OR brede school* OR “vroegste kinderjaren”) AND (“Latijns Amerika*” OR Latijns‐Amerika OR “Zuid Amerika* OR “Zuid‐Amerika* OR Centraal‐Amerika” OR Centraal Amerika” OR Antigua* en Barbuda OR Argentinie* OR Argentinië* OR Aruba OR Bahama's OR Barbados OR Belize OR Bermuda OR Bolivia* OR Brazilië* OR Brazilie* OR “Britse Maagdeneilanden” OR “Kaaimaneilanden” OR Chilli* OR Colombia* OR Columbia* OR “Costa Rica*” OR Cuba* OR Curacao OR Curaçao OR Dominica* OR “Dominicaanse Republiek” OR Ecuador* OR “El Salvador*” OR “Frans Guyana*” OR Grenada* OR Guadeloupe OR Guatemala* OR Guyana* OR Haiti* OR Haïti* OR Honduras OR Jamaica* OR Martinique OR Mexico OR Montserrat OR “Nederlandse Antillen” OR Nicaragua* OR Panama* OR Paraguay* OR Peru* OR “Puerto Rico” OR “Saint Barthelemy” OR “Saint Barthélemy” OR “Saint Kitts en Nevis” OR “Saint Lucia*” OR “Saint‐Martin” OR “Saint Vincent en de Grenadines” OR “Sint Maarten” OR Suriname OR Trinidad en Tobago OR “Turks‐ en Caicoseilanden” OR “Turks en Caicoseilanden” OR Uruguay OR “Maagdeneilanden” OR Venezuela)

We also included time frame (1990–2015) in the search parameters. We selected this time frame because it provided us with access to a large amount of relevant evidence; we also wanted to be more inclusive and make sure we did not leave out any important evidence. In addition, this time frame focuses on the period after the Education for All (EFA) movement and the World Conference on Education for All held in 1990 in Jomtien, Thailand. Based on the population criteria and time frame, we constructed a search string in five languages—English, Spanish, French, Portuguese, and Dutch—to cover the variety of literature most likely to address EGL in the LAC region.

We aimed to make the search strings as broad as possible to retrieve the maximum amount of potentially relevant items from all databases (Schuelke‐Leech, Barry, Muratori, & Yurkovich, 2015). In theory, the use of one standardized search string ensures an unbiased search strategy across all databases. In practice, using one standardized search string is challenging because the search rules are not standardized across repositories. For example, SAGE Publications has an interface that looks for two‐word and longer phrases encapsulated in double quotation marks (e.g., “early grade”). In contrast, the Thomson Reuters Web of Science research platform instructs users to include search terms/phrases in parentheses: (early grad*). The rules of using Boolean logic, including wildcards (e.g., “*” and “?”), are also different across various data sources. Furthermore, some databases impose limits on the number of queries and the length of search strings. As a result, the team modified the search string according to each database and documented the iterative process of modifying the search strings (see Appendix A).

The primary focus of the initial search for evidence was to retrieve as many potentially relevant documents from all data sources as possible. However, different data sources have different search functionalities and interfaces. For example, the SAGE Publications website only allowed us to search by a limited number of keywords (e.g., “early grade” AND literacy OR “early grade” AND reading). As a result, we had to limit our results by several journal categories (e.g., Special Education, Regional Studies, Language and Linguistics). In contrast, we were able to use the full search string at the ScienceDirect website (see Appendix A). To overcome these differences in search capabilities, we exported all 9,696 documents into a comma‐separated value file and applied a “standardized” search string across all documents using the same algorithm in Python 8.

#### 5.2.2 Electronic searches

After the systematic review team developed the broad search strings, research associates with expertise in quantitative or qualitative research used the search terms and strings (in each of the target languages) to conduct an initial search of online databases and development‐focused websites, reviewed bibliographies of accepted articles to find other potentially relevant studies, and sent out emails to EGL experts in the LAC region and beyond in order to cast a broad net and capture as much of the evidence base as possible. We used three primary methods to search for EGL evidence.

Internet searches of predefined online databases, journals, and international development organizations

The review team worked with other researchers, librarians, computer scientists, and content experts to identify appropriate online databases, journals, and international development organizations for our search.

i. **Online databases**:
  - 3ie
  - British Library for Development Studies
  - Campbell Collaboration
  - Cochrane Library
  - Dissertation Abstracts
  - Directory of Open Access Journals (DOAJ)
  - Directory of Open Access Books (DOAB)
  - Development Experience Clearinghouse (DEC)
  - Education International
  - JSTOR Arts & Sciences I–X Collections and JSTOR Business III Collection
  - SAGE Publications
  - ScienceDirect
  - Taylor & Francis
  - Wiley
  - WorldCat
  - Within EBSCO:
    – Academic Search Premier
    – EconLit
    – Education Source
    – ERIC (Education Resource Information Center)
    – Psychology & Behavioral Sciences Collection
    – PsycINFO
    – SocINDEX with Full Text

ii. **Development‐focused databases/websites:**
  - The U.K. Department for International Development (DfID)
  - The United States Agency for International Development (USAID)
  - The Joint Libraries of the World Bank and International Monetary Fund (JOLIS)
  - The British Library for Development Studies (BLDS)
  - Institute of Development Studies (eldis)
  - The International Initiative for Impact Evaluation (3ie)
  - The Abdul Latif Jameel Poverty Action Lab (J‐PAL)
  - Innovations for Poverty Action (IPA)
  - World Health Organization (WHO)
  - United Nations Educational, Scientific and Cultural Organization (UNESCO)
  - The United Nations Children's Fund (UNICEF)
  - The United Nations High Commissioner for Refugees (UNHCR)
  - Population Council
  - World Vision
  - Save the Children
  - Plan International
  - Organization of American States (OAS)

iii. **LAC region databases and websites:**
  - Latindex
  - Red de Revistas Científicas de América Latina y el Caribe, España y Portugal (Redalyc)
  - Scientific Electronic Library Online o Biblioteca Científica Electrónica en Línea (SciELO)
  - Consejo Latinoamericano de Ciencias Sociales (CLACSO)
  - Dialnet
  - eRevistas

Forward and backward snowballing of the references of key papers provided additional studies for review that may not have been found in database searches. Citation searches were conducted in Google Scholar.

#### 5.2.3 Searching other resources

##### 5.2.3.1 Gray literature

To ensure we captured all of the relevant and applicable literature in the region, we reviewed the bibliographies of accepted articles and reports to identify relevant and high‐quality studies that might fit our criteria. We then searched for these studies and applied our inclusion criteria to them.

The research team compiled a list of 43 EGL experts—particularly those from the wider LAC region—and asked them to provide additional sources of evidence that may not have been captured through the online evidence search. We used a snowball approach and asked these experts to share the contacts of others, so that we could identify other relevant research.

### 5.3 Data collection and analysis

#### 5.3.1 Selection of studies

We imported all citations found through the above search methods into the Mendeley reference management software (http://www.mendeley.com/). Mendeley automatically extracted bibliographic data from each book, article, or reference and removed all duplicates.

The following sections detail the additional steps that we took to identify the most potentially relevant articles, review them manually, and apply the strict inclusion criteria.

#### 5.3.2 Screening Phase 1: WikiLabeling

We applied Wikipedia‐based labeling and classification techniques to the abstract data to categorize and screen articles to increase the relevance of retrieved results using the well‐known online encyclopedia, Wikipedia (Egozi, Markovitch, & Gabrilovich, 2011; Gabrilovich & Markovitch, 2006). Due to the broad and inclusive nature of our search strings, much of the initial evidence we captured was not actually relevant to our review. Therefore, we applied Wikipedia‐based labeling to help us identify the most relevant pages. The process of identifying these pages is twofold: first, experts need to share a list of potentially relevant categories. Next, we had to mine Wikipedia to find pages associated with exactly these or similar categories. We then validated the resulting list with the experts again. For example, “learning outcomes,” originally proposed by our experts, maps directly to “outcome‐based education” within Wikipedia. Wikipedia's innate hierarchical structure allowed us to make our categories less ambiguous and better organize them into a meaningful list (Box 1).

Box 1 **List of relevant categories that have individual Wikipedia pages**

- Dual language
- Emergent literacies
- First language
- Fluency
- Free writing
- Grammar
- Language education
- Language proficiency
- Listening
- Literacy
- Orthography
- Outcome‐based education
- Phonemic awareness
- Phonics
- Phonological awareness
- Reading (process)
- Reading comprehension
- Second‐language
- Second language acquisition
- Spoken language
- Transitional bilingual education
- Understanding
- Vocabulary
- Writing

We combined the WikiLabeling results with the “standardized” search term strategy described in the previous section. Although WikiLabeling is generally effective at assessing the overall context of a document and its relevance to a given subject, the search term strategy helps narrow down the search by specific keywords and phrases, such as individual countries and the region name. We used this approach to categorize documents in all target languages (English, French, Spanish, Dutch, and Portuguese).

The “standardized” search term strategy and WikiLabeling are complementary in several important ways:

- Search terms and regular expressions help discover individual words and phrases within a document, no matter where they appear. For example, the geographic region may be mentioned only in the discussion part of a paper when writing about broader potential impacts. Meanwhile, the main body of the paper might have nothing to do with Latin America or the Caribbean (e.g., we have seen some studies evaluating an intervention in sub‐Saharan Africa, which mention other developing countries that could learn from this experience). In contrast, Wikipedia‐based labeling assesses the entire context of the document by comparing all words and phrases used in academic papers and comparing them to the ones used to describe individual concepts, such as “language education” or “phonological awareness.”
- Search strings can cover a wide range of inclusion criteria and be structured to include three or four different variables. WikiLabeling looks into every concept individually and therefore provides a more in‐depth assessment of the relevance of a document for the subject of focus.
- Search term strategies are more flexible and do not depend on the user community curating an online encyclopedia every day. However, the continuous curation in Wikipedia helps improve the quality of knowledge and introduce new meaningful concepts into the scientific language through discovery and analysis applied in WikiLabeling.

For this review, we used the search term strategy followed by Wikipedia‐based labeling and classification to define which documents were most likely to be relevant for the subject in focus. This computational approach can be considered largely systematic and unbiased in how it decides the relevance of documents on a given subject. Both the search term strategy and Wikipedia‐based labeling apply standardized approaches and offer several methods of robust evaluation and validation.

Importantly, our approach supplements but does not replace the human review of potentially relevant articles. We built in several quality control procedures to ensure that our algorithm did not lead to the exclusion of relevant papers. We created four samples, with 100 abstracts each. Within each sample we included a set of 80 randomly selected abstracts that were retrieved by the search strategy, WikiLabeling, or both. The remaining 20 documents were randomly selected from the subset not retrieved by any of our approaches (i.e., 8,145 documents that were considered as irrelevant by the search strategy, WikiLabeling, or both). We then distributed these samples to four senior reviewers and reading experts and asked them to identify the irrelevant articles. This process enabled us to check for both false negatives (articles not retrieved through our search approach—the 20—but which were deemed relevant) as well as false positives (articles retrieved through our search approaches—the 80—but which were deemed irrelevant).

##### 5.3.2.1 Phase 2: Applying inclusion criteria and recording key indicators

After narrowing down our list of articles through WikiLabeling, we imported all remaining 1,824 citations back into the Mendeley reference manager software. We divided citations among reviewers, who applied the predetermined inclusion criteria (see Table 2) to each title and abstract. We chose to err on the side of sensitivity rather than specificity during our initial title and abstract review. Our inclusion criteria were purposefully broad because we did not want to miss any relevant citations due to narrow inclusion criteria. Any article that did not meet one of the following five threshold criteria laid out in Table 2 was automatically excluded from further review.

**Table 2:** Initial inclusion criteria for early grade literacy evidence

#	Category	Criteria	Notes
1	Year of publication	Include *literature* from the last 25 years, a time frame spanning 1990–2015	● If unpublished, the research must have been conducted in that time frame
2	Relevance to the region	The evidence must be from or on the LAC region including any or all of the following: Antigua and Barbuda, Argentina, Aruba, Bahamas, Barbados, Belize, Bermuda, Bolivia, Brazil, British Virgin Islands, Cayman Islands, Chile, Colombia, Costa Rica, Cuba, Curacao, Dominica, Dominican Republic, Ecuador, El Salvador, French Guiana, Grenada, Guadeloupe, Guatemala, Guyana, Haiti, Honduras, Jamaica, Martinique, Mexico, Mont Serrat, Netherlands Antilles, Nicaragua, Panama, Paraguay, Peru, Puerto Rico, Saint Barthelemy, Saint Kitts and Nevis, Saint Lucia, Saint‐Martin, Saint Vincent and the Grenadines, Sint Maarten, Suriname, Trinidad and Tobago, Turks and Caicos Islands, Uruguay, U.S. Virgin Islands, Venezuela	● We will not include research on migrants from the LAC region residing outside the region
3	Relevance to the population	Boys or girls ages birth through Grade 3 in the LAC region, regardless of the age of the child. If they are enrolled in Grade 3 or below, they fall within our population	● We will include all research that focuses at least partly on this age group even if other populations of interest are included
4	Relevance to the topic	The literature must have a focus on reading or literacy (which includes reading and writing)	We will include all research focusing at least partly on reading or literacy even if it addresses multiple areas. We will not include research that could have an effect on reading but does not actually discuss that link (e.g., IQ studies) Research on writing will be included automatically if it also discusses the link to reading or literacy
5	Is it research?	There must be a research question or research objective and a methodology that matches that objective	● If the document is a literature review or systematic review, then we would not include it in our review. We would instead focus on the primary studies cited in that literature review

Abbreviation: LAC, Latin America and the Caribbean.

During the title and abstract review, reviewers selected “yes,” “no,” “unclear,” or “not rated” on the Excel spreadsheet for each of the inclusion criteria (i.e., published since 1990, from or on the LAC region, ages birth to Grade 3, reading or literacy focused, and includes a research question or objective). Here is an explanation of each option:

- Marking “yes” for any of the five criteria indicated that the reviewer should continue onto the next criterion on the coding sheet. If the reviewer marked “yes” to all of the inclusion criteria, then they were required to fill in the remaining indicators outlined in Table 3.
- Marking “no” indicated that the reviewer should stop because the study did not meet the criteria for further review. In this case, the remaining inclusion criteria were automatically marked as “unrated,” signifying that the study failed to meet one of the inclusion criteria and thus, whether it met the other criteria was no longer relevant.
- Marking “unclear” indicated that the study was tagged for review by a senior technical expert who was equipped to determine relevance. At this stage, we followed the motto “When in doubt–include,” and maintained a record of all excluded articles indicating for what criteria they were excluded.

**Table 3:** Key indicators for early grade literacy evidence

Categories	Selection choices
Abstract number	
Citation information	
Abstract	
Document reviewer name	
Country(ies) of focus	Antigua and Barbuda, Argentina, Aruba, Bahamas, Barbados, Belize, Bermuda, Bolivia, Brazil, British Virgin Islands, Cayman Islands, Chile, Colombia, Costa Rica, Cuba, Curacao, Dominica, Dominican Republic, Ecuador, El Salvador, French Guiana, Grenada, Guadeloupe, Guatemala, Guyana, Haiti, Honduras, Jamaica, Martinique, Mexico, Mont Serrat, Netherlands Antilles, Nicaragua, Panama, Paraguay, Peru, Puerto Rico, Saint Barthelemy, Saint Kitts and Nevis, Saint Lucia, Saint‐Martin, Sint Maarten, Saint Vincent and the Grenadines, Suriname, Trinidad and Tobago, Turks and Caicos Islands, Uruguay, U.S. Virgin Islands, Venezuela, or multiple countries
Region	South America, Central America, Caribbean, North America
World Bank income level	Low income, lower‐middle income, upper‐middle income, high income non‐OECD, high‐income OECD
Type of document	Journal article, technical report, dissertation/thesis, book chapter, other
Full text available to AIR	Yes, No, Other
Full text available to public	Yes, No, Other
How was document located?	Source bibliography, hand search of journal, online source, in‐person contact, recommended by a content expert
Language of publication?	English, Spanish, French, Dutch, Portuguese, Bilingual, Other
Target group	Early childhood, preprimary (pre‐k or kindergarten), primary, out‐of‐school children (school‐age children who are not enrolled), other
Type of evidence	*Quantitative*: Intervention‐based: Experimental, Quasiexperimental, Multivariate Regression, Univariate Regression, Graphics, Other
	*Quantitative*: Nonintervention‐based: Psychology, linguistics, reading science studies (methods include structural equation models, multivariate and univariate regressions, lab‐type pilot studies, writing system analyses, other)
	*Qualitative*: Intervention, nonintervention: Case study, focus groups, interviews, multiple methods, other
	*Mixed methods*: Includes both quantitative and qualitative methodologies

Abbreviation: OECD, Organisation for Economic Co‐operation and Development.

Reviewers then used the same Excel spreadsheet to record key indicators (Table 3) for literature that met all five inclusion criteria.^1^

#### 5.3.3 Screening Phase 2: Data extraction and management

We compiled all of the full‐text articles and books that met all inclusion criteria, as well as those that were still unclear after the title and abstract review, and assigned them to senior researchers based on language and type of study. The senior researchers reviewed the articles using separate quality review protocols based on the type of study.

#### 5.3.4 Assessment of risk of bias in included studies

Researchers reviewed the articles using separate quality review protocols (see Appendix D for full versions of each protocol) based on the type of study as follows:

##### 5.3.4.1 Assessment of risk of bias quantitative studies

We used an adapted version of a risk of bias (RoB) assessment tool developed by Hombrados and Waddington (2012). Specifically, we assessed the risk of the following biases:

(1) **Selection bias** and confounding, based on the quality of the identification strategy used to determine causal effects and assessment of equivalence across the beneficiaries and comparison or control group
(2) **Performance bias**, based on the extent of spillovers to the students in the control or comparison groups and contamination of the control or comparison group
(3) **Outcome and analysis reporting biases**, including:
  - The use of potentially endogenous control variables
  - Failure to report nonsignificant results
  - Other unusual methods of analysis
(4) **Other biases**, including:
  - Courtesy and social desirability bias
  - Differential attrition bias
  - Small sample sizes and no clustering of standard errors
  - Strong researcher involvement in the implementation of the intervention and the Hawthorne effect

Two or more reviewers read and rated all quantitative intervention studies to ensure consensus. The reviewers resolved disagreements in assessments through discussion or by third‐party adjudication. Reviewers reread studies several times if something was unclear and maximized the use of all the available information from the studies. Assessments were based on the reporting in the primary studies, erring on the side of caution. For example, in those cases in which it was not clear whether standard errors were clustered, we assumed they were not clustered and took that into consideration in the risk of bias assessment.

##### 5.3.4.2 Quality appraisal of qualitative studies

We adapted the Critical Appraisal Skills Programme (CASP) Qualitative Research Checklist (Critical Appraisal Skills Programme, 2018) to assess the research design, data analysis, ethical considerations, and the relevance to practice. The tool examines reviewers' responses to 11 main questions, each of which has multiple subquestions. Upon reading the full‐text article, reviewers had to select either “High,” “Medium,” “Low,” “N/A,” or “Not Mentioned” for each of the 11 questions and subquestions and provide a justification for their rating. The justification was also supported by text and page numbers from the article. Reviewers were encouraged to comment on both strengths and weaknesses when applicable. The 11 qualitative review questions were divided into three categories: research design, ethics and reflexivity, and relevance to the field as shown below:

Research design:

- Clear statement of research?
- Appropriateness of qualitative methodology?
- Addresses the aims of the research?
- Was the data collected in a way that addressed the research issue?
- Was the data analysis sufficiently rigorous?
- Is there a clear statement of findings?

Ethics and reflexivity:

- Has the relationship between researcher and participants been adequately considered?
- Have ethical issues been taken into consideration?
- Appropriate recruitment strategy?

Relevance to the field:

- How valuable is the research?Information for stakeholders to assess replicability?

In addition to these 11 quality criteria, reviewers summarized the main findings of each qualitative article. Finally, reviewers reviewed the bibliography for each article and identified other relevant references for further review. Pairs of reviewers rated the same studies at the outset to ensure a common understanding of the quality categories, but the remaining articles were reviewed by single reviewers due to time constraints.

##### 5.3.4.3 Quantitative appraisal of correlational studies

For the quantitative appraisal of correlational studies, we used an adapted version of the RoB tool for quantitative intervention studies (Hombrados & Waddington, 2012), removing any questions regarding interventions. The quantitative nonintervention quality review tool assesses the relevance, data and methodology, and analytical approach of the research by eliciting reviewers' responses to the following 18 quality criteria questions:

(1) Did the outcome measure include some measure of reading or a reading subskill (e.g., fluency, PA, language, decoding, letter knowledge, comprehensions etc.)?
(2) If the study did not include a measurement of reading or a reading subskill, is literacy measured in a different manner?
(3) Is the sample selection criteria/justification provided?
(4) Is there data reported on covariates?
(5) Is there information on training test administrators?
(6) Are outcomes collected through self‐reports?
(7) How was language of reading data collection determined?
(8) Did the study report data collection procedures (e.g., quiet room, during school hours, possible fatigue effects)?
(9) Was the unit of allocation and the unit of analysis the same?
(10) Do all students targeted by the study take the reading test/answer the survey questions?
(11) Does the study take into consideration potential data collection implementation failures?
(12) Does the study have a strong conceptual or theoretical framework?
(13) Do the authors generalize only to the reading outcome, and population applicable from the sample?
(14) Do the authors argue convincingly that it is not likely that being monitored influences the behavior of study participants?
(15) Are there appropriate reliability scores for all tests?
(16) Does the study describe the analysis method?
(17) Does the study justify the analysis method (is the analysis method appropriate for the research question/objective)?
(18) Were any participants not included in the analysis? If so, is there justification for why?

Upon reading the full‐text article, reviewers responded to each question by selecting “Yes,” “No,” “Unclear,” or “N/A” and provided a justification for the rating, citing the text whenever possible. Finally, reviewers provided a summary of the article's main findings and their relevance to target stakeholder groups.

In order to synthesize the findings of the quantitative nonintervention research, we first determined which studies should be included in the analysis. To achieve this goal, we referred to the quality protocols filled out by the reviewers for each article and only included studies that were considered high quality. For instance, if there was missing information about data administration or no information provided about how the language of testing was determined, we did not dismiss the study; however, if the reviewers judged that there were notable problems with the method or sample selection, we did not include the study in our analysis.

The below seven ratings from the protocol were considered key to determining inclusion as they ensure that the study is focused on reading and has a strong research design and methodology:

(1) Did the outcome measure include some measure of reading or a reading subskill?
(2) Is the sample selection criteria/justification provided?
(3) Did the study report data collection procedures?
(4) Does the study have a strong conceptual or theoretical framework?
(5) Are there appropriate reliability scores for all tests?
(6) Does the study describe the analysis method?
(7) Does the study justify the analysis method?

##### 5.3.4.4 Quantitative appraisal of mixed‐methods studies

Reviewers completed both a quantitative and a qualitative quality review protocol for mixed‐methods articles.

#### 5.3.5 Measures of treatment effect

We extracted information from each quantitative study to estimate standardized effect sizes. In addition, we calculated standard errors and 95% confidence intervals if possible. We calculated the Hedges' *g* sample‐size‐corrected SMDs for continuous outcome variables, which measures the effect size in units of standard deviation of the outcome variable.

We first calculated SMDs (Cohen's *d*) by dividing the mean difference with the pooled standard deviation by applying the formula in Equation (1):

(1) $$\text{SMD}=\frac{\text{Yt}-\text{Yc}}{\text{Sp}}.$$

SMD refers to the standardized mean differences, Yt refers to the outcome for the treatment group, Yc refers to the outcome for the comparison group, and Sp refers to the pooled standard deviation.

The pooled standard deviation Sp can be calculated by relying on the formulas in Equations (2) and (3):

(2) $$\text{Sp}=\frac{\sqrt{\left(\left({\text{SDy}}^{2}\right)⁎\left(\text{nt}+\text{nc}-2\right)\right)-\left(\frac{\beta^{2}⁎(\text{nt}\times \text{nc})}{\text{nt}+\text{nc}}\right)}}{\text{nt}+\text{nc}}.$$

(3) $$\text{Sp}=\frac{\sqrt{\left(\text{nt}-1\right)\times {\text{st}}^{\begin{array}{c} 2 \end{array}}+\left(\text{nc}-1\right)\times {\text{sc}}^{2}}}{\text{nt}+\text{nc}-2}.$$

We used Equation (2) for regression studies with a continuous dependent variable. In this equation, SDy refers to the standard deviation for the point estimate from the regression, nt refers to the sample size for the treatment group, nc refers to the sample size for the control group, and *β* refers to the point estimate. We used Equation (3) when information was available about the standard deviation for the treatment group and the control group.

We corrected the standardized mean difference for small sample size bias by relying on Equation (4), which transforms Cohen's *d* to Hedges' *g*.

(4) $$\text{SMDcorrected}=\text{SMDuncorrected}\times \left(1–\;\frac{3}{4\times \left(nt+nc-2\right)-1}\right).$$

We also relied on Equation (5) to estimate the standard error of the standardized mean difference:

(5) $$\text{SE}=\sqrt{\frac{\text{nt}+\text{nc}}{\text{nc}\times \text{nt}}+\frac{{\text{SMD}}^{2}}{2\times (\text{nc}+\text{nt})}}.$$

#### 5.3.6 Unit of analysis issues

Where the standard error did not take clustering of outcomes into account in the estimation of standard errors (that is, where the outcome variables were likely to be clustered at a higher level of aggregation than the student level but this was not taken into consideration in the estimation of the standard errors and confidence intervals), we used adjusted standard errors. For these studies with a risk of unit of analysis error, we applied corrections to the standard errors and confidence intervals using the variance inflation factor (Higgins & Green, 2011):

SEcorrected=SEuncorrected×√(1+(m−1)×ICC).

Here, *m* is the number of observations per cluster and ICC is the intracluster correlation coefficient.

To identify the ICC, we relied on a study by Yoshikawa et al. (2015), who estimated the ICC for reading outcomes of students clustered in schools in Chile. They found an ICC of 0.10. Although this estimate is most likely not externally valid for the rest of the LAC region, it is our best estimate of the ICC that is available to us. Thus, we rely on this estimate for our effect size calculations.

#### 5.3.7 Methods for handling dependent effect sizes

We included only one effect size per study in a single meta‐analysis. Where studies reported more than one effect size on the basis of different statistical methods, we selected the effect size with the lowest risk of bias. Where studies presented several impact estimates for different variables that measure the same reading construct, we used a sample‐size weighted average to measure a “synthetic effect size.” Examples of reading constructs include decoding, vocabulary acquisition, and reading comprehension. Importantly, there were insufficient studies that reported impacts on more than one reading construct. The majority of the studies that we were able to include in the meta‐analysis only determined the impact of the evaluated program on a standardized language assessment for the grade level. Furthermore, the majority of the studies did not provide enough information about the assessment of reading to determine which reading constructs were measured. For example, none of the included studies provided details about the contents of the assessment test. Thus, we did not conduct separate meta‐analyses for more than one reading construct because there was insufficient information about effect sizes for different reading constructs. Therefore, we assumed that the effect sizes were similar for different reading constructs or calculated synthetic effect sizes. This approach does not allow us to examine separate impacts on different reading constructs. Furthermore, it requires the assumption that effect sizes are not dependent upon the specific reading construct that is used as an outcome variable. These assumptions are not necessarily realistic, but we needed to make them in order to enable a meta‐analysis across studies. To mitigate these concerns, we complemented the meta‐analysis with a narrative review approach. In addition to the meta‐analysis for EGL outcomes, we were able to conduct a meta‐analysis to determine the effects of nutrition programs on early grade spelling outcomes.

We also calculated synthetic effect sizes for different grades and different age groups and assumed homogenous effects across age groups when heterogeneous effects were not reported. We did not find sufficient studies that reported separate effects for different grades or age groups to report separate meta‐analyses by grade or age group. We also found several studies that only reported average effects for students that meet our inclusion criteria (Grade 3 and below) and students that did not meet our inclusion criteria. We include heterogeneous effect sizes for Grade 3 and below when this information is available as in Barrera‐Osorio and Linden (2009). However, other studies only reported average effects for students in different age groups. In these case, we decided to include a homogenous effect size that assumes the effects are equivalent for each of these age groups. Again, this assumption may not be realistic, but we needed to make this assumption to enable a meta‐analysis. To mitigate this concern, we complemented the meta‐analysis with a narrative review.

#### 5.3.8 Dealing with missing data

If it was not feasible to estimate the effect size because of missing information, we contacted the authors of the primary studies to request the missing information required to calculate the effect sizes, but we ultimately were not successful in retrieving the required information to calculate effect sizes in this way. If we could not retrieve the missing data, we extracted or imputed effect sizes and associated standard errors based on commonly reported statistics such as the *t* or *F* statistic or exact *p* or *z* values using David Wilson's practical meta‐analysis effect‐size calculator. We did this for one primary study (Bando, 2010). When studies did not report sample sizes for the treatment and the control or comparison group, we assumed equal sample sizes across the groups. We did this for three primary studies (Cardoso‐Martins et al., 2011; Cristia et al., 2012; Maluccio et al., 2009).

#### 5.3.9 Quantitative data synthesis

##### 5.3.9.1 Meta‐analysis

We conducted separate meta‐analyses to determine the effects of nutrition programs, teacher training programs, and technology in education programs because these were the three topics for which we had sufficient numbers of studies for a meta‐analysis.

We reported effect sizes for individual studies when we did not have a enough studies for a meta‐analysis or when all studies had a high risk of selection‐bias. However, we were only able to estimate effect sizes for a small number of studies that were not included in the meta‐analyses.

##### 5.3.9.2 Subgroup analysis and investigation of heterogeneity

We started with separate meta‐analyses of RCTs and quasiexperimental evaluations for determining the effects of each of the programs. In addition, we pooled RCTs and quasiexperimental studies in one meta‐analysis.

When the number of studies allowed for it, we examined the heterogeneity of the effect sizes for each outcome across studies. We examined heterogeneity by using *I* ^2^ and *Q* as well as *τ* ^2^ and the visualization of the forest plots (Borenstein, Hedges, Higgins, & Rothstein, 2009). However, we only interpreted heterogeneity for meta‐analyses that included four or more studies. We used Stata (StataCorp) to conduct the meta‐analysis.

Further, we used random‐effects meta‐analysis because the average effect of programs that influence reading outcomes is likely to differ across contexts due to differences in program design or contextual characteristics. This approach is in line with the approach used in a recent systematic review on the effects of women's self‐help groups on women's empowerment (Brody et al., 2015).

##### 5.3.9.3 Assessment of publication bias

We assessed the potential for publication bias using funnel plots based on impact estimates for the studies on nutrition and ICT programs that were included in the meta‐analyses. In addition, we conducted the Egger's test. For other outcome measures, our sample size was insufficient for funnel plots to be informative about the potential for publication bias.

#### 5.3.10 Methods to synthesize qualitative and quantitative nonintervention studies

After using the quality protocols to review full‐text qualitative and quantitative nonintervention articles, we coded the protocols using NVivo qualitative data analysis software (Version 10, 2012; QSR International Pty Ltd.). NVivo is traditionally used to manage and code empirical (or field) data (Bhattacharyya, 2004; Caldeira & Ward, 2003; Patashnick & Rich, 2004). It is also used for secondary data in document analysis, such as reports, websites, and other sources. A team of analysts trained in using the qualitative software program conducted the data analysis process by coding and analyzing the quality ratings and justifications for each study.

To code and analyze the quality ratings and justifications for each article, we created three separate NVivo files for the qualitative intervention research, qualitative nonintervention research, and quantitative nonintervention research. Once we coded the quality criteria and justifications in NVivo, reviewers compared the quality of each criterion across all articles of a research type. For example, a reviewer could compare the quality of the statement of research across all qualitative intervention studies. We then wrote up a synthesis of the findings for each quality criterion for each research type using the NVivo coding structure.

To synthesize the study findings for each research type, we also used NVivo as a tool for qualitative research. Analysts created separate NVivo files for intervention and nonintervention research and imported the reviewers' statements of findings for each included study. They then coded these statements of findings into topic nodes (these were predetermined by literacy experts as covering the main areas of EGL).

Once the coding was complete, the analysts were able to see the findings for each topic area and could then write up the analysis and implications by topic area. The topic nodes included Child Nutrition, Classroom Methodologies, Disabilities, Early grade reading assessments, Language skills for reading, Learning to read in a mother tongue, Learning to read in an L2 or additional language, Literate environment, Longitudinal Research on Reading, Neuroscience of reading, Other, Parental and Community participation, Pre‐Literacy, Print and decoding skills for reading, Reading Habits, Steps in learning how to write, and Teacher training.

We only included findings for high‐ and medium‐quality articles in our synthesis for qualitative studies. To determine which qualitative studies were of sufficient quality to report on the findings, we created an Excel file with all 26 qualitative intervention and nonintervention studies as well as their ratings on each of the quality criterion. This enabled us to see all of the ratings in one view and determine if a study was strong enough to be included. We could then refer back to the original protocol and the reviewers' justifications to make sure that the study met certain criteria such as having a research question, matching methodology, transparent methods of analysis, substantiated findings, and so forth.

#### 5.3.11 Triangulating findings

After conducting the quality review and synthesis of articles, reviewers triangulated the different syntheses by linking the evidence back to the conceptual framework. We examined the impact of the different programs on EGL outcomes and triangulated these findings with the qualitative research to examine whether the fidelity of implementation or experiences and perspectives of different stakeholders may have influenced the impact of these programs. In addition, we assessed the predictors of reading outcomes to increase our understanding of the linkages between intermediate outcomes, such as teacher knowledge and behavior, and reading outcomes. Finally, we used the information from the qualitative research to examine whether and where any links in the conceptual framework broke down. Findings from the qualitative synthesis and the quantitative nonintervention synthesis helped describe, explore, and interpret how specific programs improve reading outcomes.

The triangulation of findings from different research methods allowed us to define and test hypotheses using different methodologies that informed and supplemented each other. This approach allowed us to capture the state of the evidence on whether and how specific programs improve reading outcomes in Latin America as well as the gaps in the evidence.

## 6 DEVIATIONS FROM THE PROTOCOL

### 6.1 Deviations

We deviated from the protocol (American Institutes for Research, 2015) in four main ways. First, we did not conduct a hand search of journals as we had originally intended in our search protocols because of time constraints. Second, we only conducted meta‐analyses to determine the impact of teacher training, technology in education, and nutrition programs because the number of high‐quality studies for other intervention types (e.g., school governance, preschool, teacher practices, parent practices, etc.) for which we were able to calculate effect sizes were not sufficient for a meta‐analysis. Instead, we used narrative synthesis techniques to report on the results of other intervention types. Third, we planned to examine the heterogeneity of the effect sizes visually and by estimating the *I* ^2^ and *Q*, as well as *τ* ^2^ (Borenstein et al. 2009). However, the number of studies included in the meta‐analyses was often too small to obtain reliable estimates of the heterogeneity of the effect sizes. In practice, we only examined heterogeneity for meta‐analyses that included four or more studies. Fourth, we planned to perform a sensitivity analysis for two methodological effect size moderators:

- Risk of bias status for each risk of bias category and
- Study design (RCTs vs. quasiexperimental studies).

However, we were again often not able to conduct such sensitivity analyses because of the small number of studies in the meta‐analyses. We only examined heterogeneity for meta‐analyses that included four or more studies. In these cases, we also examined whether RCTs could be credibly pooled with quasiexperimental studies by conducting a meta‐regression to assess whether RCTs and quasiexperimental studies show statistically significantly different point estimates.

## 7 RESULTS

### 7.1 Results of the search

Our literature search aimed to identify all existing intervention‐ and nonintervention‐based studies and existing literature from or on the LAC region involving reading programs, practices, policies, and products focused on improving reading skills for children from birth through Grade 3.

**Figure 4:**
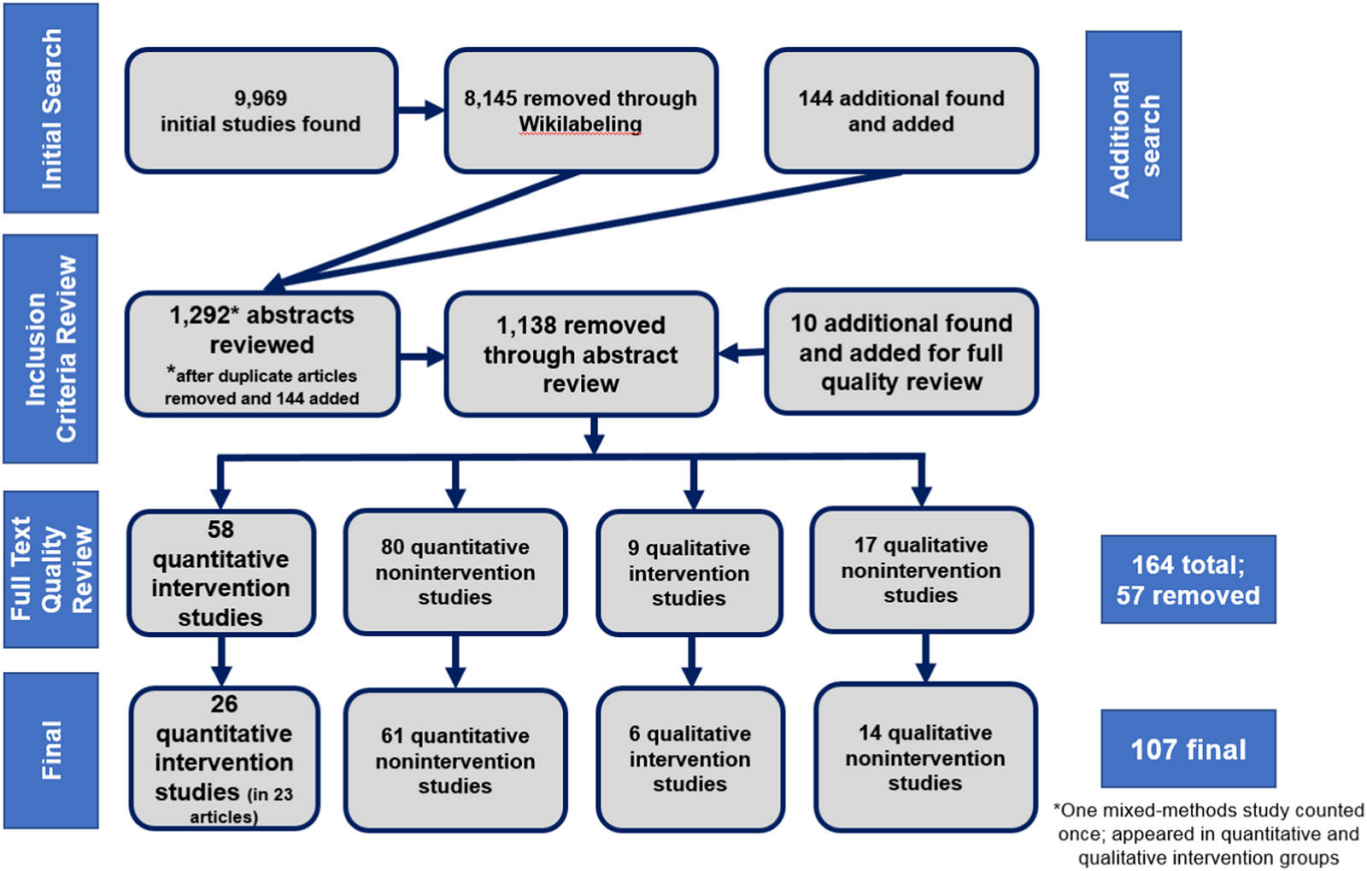
Systematic review phases: initial search to quality review

We conducted the search from July to August 2015 and applied the WikiLabeling approach in September 2015. We finalized the search in February 2016. Figure 4 depicts the systematic review phases from initial search through quality review. It indicates the number of studies that passed into each subsequent phase of review as well as the numbers of studies that were removed at each phase.

We found 9,696 studies using our search strings and modified strings for all online sources. We applied WikiLabeling in order to identify the most relevant of the 9,696 documents and removed 8,145 documents that were identified as irrelevant.

We retrieved 144 additional articles through other search engines that we identified as having potentially relevant research. We reviewed these articles against the inclusion criteria along with the articles identified through WikiLabeling for a total of 1,292 articles reviewed. During this stage, reviewers applied the five inclusion criteria to titles and abstracts and an additional 1,138 articles were rejected (see Appendix E for details on the number of articles rejected for each inclusion criterion).

One hundred sixty‐four articles moved on to the full‐text quality review. The quality review protocols were applied during this phase to 154 articles that either met all five inclusion criteria or met all criteria with one or more criteria listed as unclear (i.e., it could not be determined from reviewing the abstract whether it met the criteria), plus 10 additional studies that were identified through web searches or snowballing of references and met all inclusion criteria. These articles were reviewed in their entirety against the quality review protocol.

During this stage, we rejected an additional 57 articles for the following reasons:

- We were not able to access the full text of the article.
- During the inclusion criteria review, reviewers marked many articles as “unclear.” Upon reviewing the full text, reviewers were able to determine that the articles did not meet the inclusion criteria.
- The article was identified as low quality.

### 7.2 Included studies

The full searching process previously described led to the inclusion of 107 quantitative and qualitative studies. Of these, 32 were intervention studies while 75 were nonintervention studies. We included 26 quantitative intervention studies (both experimental and quasiexperimental) that evaluated the effects of 23 unique programs or program components on reading outcomes. The review of qualitative intervention studies led to the inclusion of six articles. Additionally, we included 14 articles in the review of qualitative nonintervention studies. Finally, we included 61 quantitative noninterventions studies.

Table 4 summarizes the characteristics of all articles included in the final review. The articles are categorized by publication type, year of publication, region and country of focus, language of publication, research type, and the country of focus income level (as determined by the World Bank).

**Table 4:** Characteristics of the final included reviews

	*N*	%
Publication type		
Dissertation/thesis	3	3
Journal article	96	90
Technical report	5	5
Working paper	3	3
Year of publication		
1990–1995	5	5
1996–2000	13	12
2001–2005	15	14
2006–2010	25	23
2010–2016	49	46
Region and country of focus		
Caribbean		12
Cuba	2	
Jamaica	6	
Puerto Rico	5	
Central		5
Costa Rica	1	
Guatemala	4	
North		17
Mexico	18	
South		63
Argentina	10	
Brazil	27	
Chile	15	
Colombia	6	
Guyana	1	
Peru	7	
Uruguay	1	
Venezuela	1	
Multiple countries	3	3
Language of publication		
English	62	58
Portuguese	14	13
Spanish	31	29
Type of research		
Qualitative intervention	6	6
Qualitative nonintervention	14	13
Quantitative intervention	26	24
Quantitative nonintervention	61	57
Country of focus income level (World Bank)		
Lower‐middle income	5	5
Upper‐middle income	78	73
High income	20	19
Not applicable/multiple countries	4	4

Appendix C summarizes the main characteristics of the included studies including program characteristics, outcome measures, sample size, study design, and analysis.

#### 7.2.1 Population and settings

More than 90% of the articles were focused on high‐ to upper‐middle‐income countries. The disproportionate emphasis on high‐income and upper‐middle‐income countries may be explained by the limited available resources and capacity for conducting high‐quality research in low‐income and lower‐middle‐income countries.

#### 7.2.2 Description of the interventions

##### 7.2.2.1 Quantitative interventions

Of the 23 interventions evaluated in the included studies, two were teacher training programs. We also included three studies estimating the impact of technology in education programs. Five studies estimated the impact of nutrition programs and two studies evaluated the impact of a school governance program. Additionally, two studies evaluated preschool programs and six estimated the impact of the adoption of distinct teacher practices, such as the explicit instruction of new words, shared storybook reading, and read‐alouds. Finally, there were three studies estimating the impact of parental involvement interventions.

##### 7.2.2.2 Qualitative interventions

The review of qualitative research on EGR interventions in the LAC region included six articles from Argentina, the Caribbean, Colombia, Jamaica, Peru, and the U.S. Virgin Islands. These six articles focus on bilingual/multilingual education in Peru (Neugebauer & Currie‐Rubin, 2009), curriculum in Jamaica (Roofe, 2014), parental and community participation in Argentina (Stein & Rosemberg, 2012), general pedagogical strategies in Colombia and the U.S. Virgin Islands (Gonzalez, Saenz, Bermeo, & Chaves, 2013; Mahurt, 1993), and teacher training in the Caribbean (Warrican, Down, & Spencer‐Ernandez, 2008).

#### 7.2.3 Outcomes

The included studies estimated the impact of programs on outcome measures such as reading comprehension, reading fluency, letter naming, word recognition, phonemic segmentation fluency, decoding, spelling, language test scores, and national literacy exam test scores. Two other studies focused on more intermediate outcomes such as reading practices (Beuermann, Cristia, Cueto, Malamud, & Cruz‐Aguayo, 2015; Tapia & Benítez, 2013).

Each of the outcome measures can be considered part of a different construct. Reading is a broad concept that can be subdivided into many different constructs. Authors of primary studies use many different operational definitions to measure reading outcomes and practices. Some studies construct indices based on different elements of reading outcomes, while others are more specific in their definition of reading outcomes or practices.

Both approaches have their advantages. Relying on an index addresses the so‐called “indicator soup” problem, which refers to the difficulty of organizing and interpreting results with many outcome variables (King, Samii, & Snilstveit, 2010). However, the construction of indices can also be accompanied by a loss of detail, for example, when interventions have positive effects on decoding, but not on language comprehension.

To mitigate these concerns, we planned to use an iterative approach. We proposed to synthesize the evidence on what works to improve EGL outcomes by conducting two types of analyses. The first analysis would pool all studies that include an outcome measure related to reading outcomes regardless of the specifics of the construct (except for reading practices). The second analysis would then examine the impact of the included programs on different components of reading outcomes, such as decoding, letter recognition, and reading comprehension.

Importantly, however, we were limited in our ability to conduct the second analysis because in several cases it was not entirely clear from the study report whether outcome measures should be considered a decoding, vocabulary acquisition, or a reading comprehension construct. Thus, in practice, we only conducted a narrative review to determine the impact of the programs on specific components of reading outcomes. In some cases, this narrative review was limited to only one study because we did not encounter more than one study that focused on that specific reading construct.

Although most of the included studies only emphasized one outcome measure related to EGL, several studies included more than one outcome measure. Of the 25 program evaluations, 15 included only one outcome measure. Furthermore, of the 25 evaluations, eight evaluations relied on a language test score to measure the impact of the program, five evaluations assessed the impact of the program on reading comprehension, four determined the impact on vocabulary acquisition, two studies focused on early literacy or letter naming, and two evaluations emphasized the impact of the program on reading practices. Other outcome measures that were included in at least one study were word reading, phonemic segmentation, decoding, spelling, English language test scores, and an undetermined measure of literacy outcomes.

Some studies relied on existing or administrative data to determine the impact of the program, while others collected their own reading outcome data. Specifically, of the included studies, 12 studies relied exclusively on existing or administrative data to determine the impact of the program, while the remaining studies collected their own data. Unfortunately, none of the studies presented details about how the assessment test was aligned with the evaluated program so we were not able to assess over‐alignment of the assessment test with the program design. It is important to note that the studies that relied on existing or administrative data had a much larger average sample size than the studies that collected their own data. We discuss the sample size of the included studies in more detail below following a discussion about the context in which the studies took place.

#### 7.2.4 Nonintervention studies

Out of 61 nonintervention studies, 57 had an outcome measure of reading or a reading subskill. In general, PA and reading were measured. Reading measures ranged from word level reading to reading connected text. One example of a study that focused on the essential components of reading and included writing was Plana and Fumagalli (2013). In contrast, some studies focused only on decoding (Jaichenco & Wilson, 2013). One study in the sample measured reading in a different manner than through PA or reading comprehension. Silva et al. (2014) measured students' narrative skills using a wordless picture book that students used to construct a story.

The majority of the studies used reading assessment tests to measure reading outcomes, which reduces the risk of measurement error. Only six of 61 studies in the sample reported information on self‐reports. These involved student (Cervini, 2015), parent (Salazar‐Reyes & Vega‐Pérez, 2013), or teacher surveys (Janus, 2011).

#### 7.2.5 Study designs and methods

##### 7.2.5.1 Quantitative interventions

In order to be included in this report, the quantitative intervention studies needed to use an experimental or a quasiexperimental design to determine the impact of the program of interest. The study designs of the included studies were diverse. Of the 23 included program evaluations, 16 relied on a RCT to determine the impact of the programs. Of these 16 evaluations, seven used a cluster RCT where the program was implemented at the school‐level as opposed to the student‐level. Of the seven remaining studies, four used propensity score matching designs and three used multivariate regression analyses to determine the impact of the evaluated programs on reading outcomes. Cluster‐RCTs are the strongest design for making causal claims about the impact of education programs, but under certain conditions, student‐level RCTs or quasiexperimental designs can also determine causal effects.

##### 7.2.5.2 Quantitative nonintervention studies

Most of the quantitative nonintervention studies in the sample, 55 of 61, gave a description of the analysis methods used. Some studies provided ample description of the statistical analyses conducted (Páez, Tabors, & López, 2007) while others gave brief descriptions and used simple analyses such as histograms (Bandini, Oliveira, & Souza, 2006). One study did not provide a description of the analysis (Melchiori, de Souza, & de Rose, 2012). In 43 out of 61 studies in the sample, all students were tested. Reasons for excluding students from the sample included: that they were absent (Cardoso‐Martins & Da Silva, 2010), researcher error (De Abreu & Cardoso‐Martins, 1998), or because of age (Rindermann, Stegmaier, & Meisenberg, 2014). Ten studies in the sample did not specify this information in their report. Analyses of quantitative nonintervention studies utilized correlational analyses including linear and multiple regressions, analysis of covariance, and analysis of variance. Other studies included only descriptive statistics, percentage counts or scores, *t* tests, or weighted averages. Analyses are discussed in further detail in the quantitative nonintervention study section.

##### 7.2.5.3 Qualitative interventions

In contrast with the quantitative studies, the qualitative studies had no requirements for the type of analysis conducted to be included because authors may have described the same type of analyses differently, making it difficult to neatly categorize the types of analysis. Most of the qualitative intervention studies analyzed data using thematic analysis (e.g., Mahurt, 1993; Roofe, 2014), one study identified themes, but did not specifically mention thematic analysis (Warrican et al. 2008). Another analysis included a description of the constant comparative method (Stein & Rosemberg, 2012). Though the majority of studies described some aspects of analysis, most studies lacked detail in how categories of interest were identified and how data supported the categories.

##### 7.2.5.4 Qualitative nonintervention

Close to half of the qualitative nonintervention studies also primarily used thematic analysis (e.g., Jiménez, Smith, & Martínez‐León, 2003; Kinkhead‐Clark, 2014). Additional articles described analyzing data by identifying themes, though the articles did not specifically mention thematic analysis (Rosado & Campelo, 2011). Other analysis methods included the constant comparative method (Manrique & Borzone, 2010) and discourse analysis (Guevara & Ordoñez, 2012).

##### 7.2.5.5 Publication type

The vast majority of studies included in our review of evidence were published journal articles and came from either Mexico or South America with significantly fewer from Central America and the Caribbean. The only Central American countries represented were Costa Rica and Guatemala, and for the Caribbean, Puerto Rico, Jamaica, and Cuba were represented. Almost all articles were published in English or Spanish. We found no articles in any regional languages.

##### 7.2.5.6 Excluded studies

The full searching process led to the exclusion of 1,148 studies. About 50% of these articles were rejected because they did not focus on the LAC region. Two hundred fifteen articles were rejected because they did not include children in grade 3 and below. Additionally, 134 studies were excluded because they did not focus on reading and 60 were dropped because they were not research papers.

### 7.3 Risk of bias in included studies

**Figure 5:**
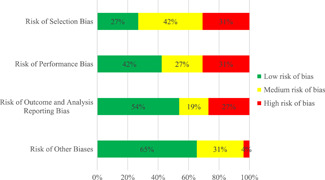
Risk of bias assessment of quantitative intervention studies

We relied on a risk of bias assessment tool with 71 questions with which we could accurately determine four types of risk of bias. The tool is an adapted version of a risk of bias assessment tool developed by Hombrados and Waddington (2012). We examined the risk of selection bias and confounding, performance bias, outcome and analysis reporting bias, and other biases. The complete risk of bias assessment tool and a detailed assessment of the risk of bias of each individual study are included in Appendix D. Figure 5 shows the distribution of low‐, medium‐, and high‐risk bias across the included studies for each of the risk of bias categories.

In general, there was agreement among the reviewers concerning assessments of the risk of selection bias, but initially there were more disagreements about the risk of performance bias, outcome and analysis reporting bias, and other biases. We reached consensus after a detailed discussion about each of the individual studies.

#### 7.3.1 Selection bias and confounding

Selection bias is associated with lack of equivalence in observable or unobservable characteristics across treatment and control/comparison groups. Selection bias may result from self‐selection into the program, which could lead to differences between students who participate in the program and students who do not participate in the program or targeting of a program to schools or students with specific characteristics. Self‐selection may result in differences in unobservable characteristics because participants in development programs are usually more motivated or entrepreneurial (Waddington et al., 2012). The targeting of a program to schools or students with specific characteristics by an implementing agency is more likely to result in differences in observable characteristics. Quasiexperimental methods such as propensity score matching are usually a good alternative to RCTs when a program is targeted to specific students or schools because in those cases it remains feasible to control for observable characteristics in the estimation of the impact of the program (Diaz & Handa, 2006). However, quasiexperimental methods such as propensity score matching usually do not allow for resolving selection bias when selection bias is caused by self‐selection because propensity score matching does not enable researchers to control for unobservable characteristics.

Of the 25 included studies, six were rated as having a low risk of selection bias, 11 were rated as having a medium risk of selection bias, and eight were rated as having a high risk of selection bias. The six studies with a low risk of selection bias were all cluster RCTs with a sufficient sample size to detect small but meaningful effects of the evaluated program on reading outcomes. For example, Cristia et al. (2012) used an RCT, in which 160 schools in Peru were randomly assigned to a program where each student received a laptop. The study relied on national test score data for more than 4,000 students. Similarly, Barrera‐Osorio and Linden (2009) used a cluster RCT with a sample of 5,201 students across 97 schools in Colombia to determine the impact of a program that distributed computers to support education.

We rated RCTs with a small sample size and quasiexperimental evaluations that used propensity score matching with a large sample as having a medium risk of selection bias. RCTs with a small sample size may suffer from lack of equivalence across the treatment and the control group because randomization requires a sufficient number of units of observation to guarantee equivalence across observable and unobservable characteristics. For example, Larraín et al. (2012) relied on a sample size of 62 children from three public kindergartens to determine the impact of more complex word elaboration on vocabulary acquisition. Such sample sizes are usually not sufficient to detect small but meaningful effects of a program on reading outcomes. Furthermore, the likelihood of publication bias is higher for studies with such low sample sizes because it is more likely that studies with such small sample sizes and statistically insignificant effects are not accepted for publication in peer‐reviewed journals (Borenstein et al., 2009). As a result, the inclusion of studies with small sample sizes may result in an overestimate of the impact of development programs on reading outcomes. The majority of the included RCTs with a small sample size also only showed limited or no baseline data to demonstrate equivalence in observable characteristics. For example, Larraín et al. (2012) did not show baseline values for the beneficiary and control students. Furthermore, Murad and Topping (2000) only showed evidence for nonsignificant differences at baseline. However, they did not present the actual values of the baseline data.

We rated studies that relied on propensity score matching and a large sample size as having a medium risk of selection bias because propensity score matching does not enable researchers to entirely control for self‐selection. The quasiexperimental studies we included did involve some self‐selection in all cases. For example, Felício et al. (2012) relied on propensity score matching to determine the impact of preschool on EGL outcomes in Brazil. However, participation in preschool is entirely dependent on self‐selection, so the use of propensity score matching does usually not allow for demonstrating causal effects of participation in preschool in these specific cases.

Finally, we rated RCTs with very small sample size and problems in the implementation of the randomization and nonexperimental studies that relied on ordinary least squares (OLS) regression analysis without a baseline as having a high risk of selection bias. Problems in the implementation of the randomization included control students that switched to the treatment group (crossovers), deliberate exclusion of part of the sample that did not comply with the randomization, and too high or unknown attrition rates. For example, Gomez Franco (2014) excluded teachers who did not comply with the instructions provided during teacher training from his analysis on the impact of a teacher training program for preschool teachers. The exclusion of these teachers from the analysis is likely to result in significant overestimates of the impact of the program. Rugerio Tapia and Benítez (2013) also relied on a sample of 10 beneficiary mothers and 10 control mothers to determine the impact of a program that encourages mothers to jointly read with their children. This sample size is likely to result in lack of equivalence across beneficiary and control mothers. Mendive et al. (2016) determined the impact of a preschool professional development program for teachers by relying on a sample with attrition rates over 50%. Such attrition rates are very likely to result in selection bias as well due to lack of equivalence across beneficiary and control students. OLS regression analysis without a baseline also does not allow for addressing selection bias. Thus, these studies should be considered as having a high risk of selection bias. For example, Campos et al. (2011) used hierarchical regression analysis to determine the impact of participation in preschool on EGL outcomes in Brazil. The use of hierarchical regression analysis does not enable researchers to control for bias from unobservable characteristics and is thus likely to result in biased impact estimates.

#### 7.3.2 Performance bias

Performance bias refers to bias that results from spillovers or contamination. Spillovers are indirect benefits of the program that result from interaction with the treatment group. These indirect benefits may, in turn, result in underestimates of the impact of the program if they are not taken into consideration in the analysis. For example, Miguel and Kremer (2004) found evidence that the effects of deworming on school enrollment were considerably underestimated when control students interacted closely with treatment students because control students are less likely to be infected with intestinal worms if they interact with dewormed treatment students. Similarly, control students may be positively affected by a program if beneficiary students help them with their homework. Contamination refers to benefits for the control group because of the unintentional assignment of the program to the control group. For example, on the ground program implementers may not know about the random assignment of schools to a program and as a result start implementing the program in the control schools. Spillovers and contamination are less likely when the assignment of the program happens at the school level. In those cases, the likelihood of interaction between treatment students and control students is lower than when treatment and control students come from the same school. Furthermore, program implementers are also less likely to make mistakes in the allocation of benefits when program assignment is at the school level than when program assignment is at the classroom or student level.

Of the 25 included evaluations, 10 studies were rated as having a low risk of performance bias, seven studies were rated as having a medium risk of performance bias, and eight studies were rated as having a high risk of performance bias. We rated studies that relied on comparisons between students in schools and found no evidence or only marginal evidence for contamination of the control group as low risk of performance bias. For example, Adrogue and Orlicki (2013) used a difference‐in‐difference analysis to identify the impact of an in‐school feeding program on reading outcomes in Argentina. Their comparison across schools is not likely to suffer from bias due to spillovers or contamination because there is no evidence of interaction between the beneficiary and comparison students.

We rated studies that relied on comparisons across students in different classrooms but within the same school and studies that found some evidence for contamination of the control or comparison group as having a medium risk of bias. For example, Murad and Topping (2000) used a sample where the beneficiary and control students came from the same school. In this case, there is a risk of spillovers because of the possibility of interaction between the beneficiary and the comparison students. This interaction may, in turn, result in indirect benefits for the comparison students, which could lead to underestimates of the impact of the program.

Finally, we rated studies that relied on comparisons between students in the same classroom and studies that found major evidence for contamination of the control group as having a high risk of performance bias. For example, one study randomly assigned students in the same classroom to a school breakfast program without taking into consideration the likely option of sharing food between students (Powell et al., 1998). In this case, the risk of contamination was considered high because of a high likelihood of food sharing. This contamination could then result in underestimates of the impact of the program.

#### 7.3.3 Outcome and analysis reporting bias

Outcome and analysis reporting bias refers to bias that results from the failure to report certain (usually nonsignificant) results and the use of unusual or incorrect methods of analysis. The failure to report specific results may indicate evidence for publication bias. For example, researchers may have incentives to only report statistically significant results and fail to report results that are not statistically significant. This failure to report results may lead researchers to overestimate the impact of programs on reading outcomes because the meta‐analysis may only include statistically significant results. Unusual estimation methods may also be an indication of outcome and analysis reporting bias. For example, researchers may choose arbitrary thresholds to ensure that results become statistically significant. Alternatively, researchers may choose to include certain control variables and exclude other control variables to ensure that results are statistically significant. Finally, incorrect estimation methods may also result in a bias in the impact estimates. For example, researchers may choose to include potentially endogenous control variables, which may result in a bias in the impact estimates.

Of the 25 included studies, we rated 13 studies as having a low risk of outcome and analysis reporting bias, five studies as having a medium risk of outcome and analysis reporting bias, and seven studies as having a high risk of outcome analysis reporting bias. Specifically, studies that reported impact estimates on all relevant outcome variables associated with reading and used appropriate estimation methods were rated as having a low risk of outcome and analysis reporting bias. For example, Pallante and Kim (2013) report impact estimates on letter naming, word recognition, vocabulary acquisition, and phonemic segmentation. This wide range of outcome measures indicates that the authors did not selectively report the impact of the program on outcome measures where they found statistically significant effects.

Studies that were selective in their reporting of heterogeneous effect were rated as having a medium risk of outcome and analysis reporting bias. For example, Simeon et al. (1995) only reported positive and statistically significant heterogeneous effects of deworming on spelling outcomes. They did not report heterogeneous effects on reading outcomes, possibly because the results were not statistically significant. Nonetheless, the authors did present average impacts on all of the included outcome measures regardless of the statistical significance of the results. Similarly, Neugebauer and Currie‐Rubin (2009) only presented impact estimates on an assessment test they developed themselves but not on a standardized assessment test.

Finally, we rated studies as having a high risk of outcome and analysis reporting bias when (a) studies did not report nonsignificant impact estimates (even if the study informally reported the lack of significance for these outcome variables in the text), (b) studies used arbitrary thresholds to determine the treatment status of certain students, and (c) studies switched control students to the control group when they did not comply with the program recommendations. For example, Mendive et al. (2016) used an arbitrary threshold to determine whether teachers were successfully implementing teacher practices following a teacher training program. They reported statistically significant effects of the compliance with appropriate teacher practices on reading outcomes. However, it remains unclear whether the results of the study were robust to the use of alternative thresholds. Felício et al. (2012) also reported only statistically significant effects of participation in preschool on reading outcomes, while they downplayed nonsignificant effects as irrelevant.

#### 7.3.4 Other biases

Other biases may include courtesy and social desirability bias, Hawthorne and John Hendry Effects, the inclusion of outcome variables that are not validated in the context of LAC, strong researcher involvement in the implementation of the program, and a failure to cluster standard errors when the program is assigned at a unit of intervention above the measurement level. Courtesy bias refers to a situation where the respondent gives the answer that he or she feels the interviewer wants to hear. Social desirability bias refers to a situation where the respondent gives the answer he or she believes is considered the socially correct answer. Self‐reported data tend to suffer from courtesy and social desirability bias (White & Phillips, 2012). Hawthorne effects refer to a bias that results from extra motivation for the treatment group because the beneficiaries know that they are part of the treatment group while John Henry effect refers to the opposite effect, where control students are motivated to catch up with the treatment group. Bias may also result from the use of outcome variables that are not validated in the context of Latin America. For example, researchers may use tests that are contextually appropriate for the United States but not for the Latin American context.

Strong researcher involvement in the implementation of the program may result in a better or worse implementation of the program than should be expected when the program is implemented at scale. In addition, strong researcher involvement may increase the likelihood of the Hawthorne effect. Finally, a failure to cluster standard errors when that is considered appropriate, such as in cluster RCTs, may result in conclusions that are too optimistic about the statistical significance of the effects of development programs on reading outcomes.

Of the 25 included studies, we rated 17 studies as having a low risk of other biases, six studies as having a medium risk of other biases, and two studies as having a high risk of other biases.

Studies that did not appear to suffer from any of the other biases mentioned above were rated as having a low risk of other bias.

Studies that experienced one (and only one) of the problems discussed above were rated as having a medium risk of other biases. For example, Vivas (1996) did not account for clustering of the standard errors in the impact estimates of a story‐reading‐aloud program on reading outcomes in Venezuela. As a result, the study may have overestimated the statistical significance of the impact estimates. In another example, Mendive et al. (2016) used videos to measure the behavior of teachers but did not take into consideration the option that teachers may have changed their behavior due to the videos. This Hawthorne effect could have resulted in a bias in the impact estimates.

Finally, studies that suffered from more than one of the other biases discussed above were rated as having a high risk of other biases. These studies are likely to be biased because they suffer from more than one other methodological problem. For example, Gomez Franco (2014) did not account for clustering of the standard errors in the impact estimates of a teacher training program for teachers in preschool in Chile. Furthermore, the impact estimates presented in this study may also be biased due to the use of videos to measure teacher behavior.

### 7.4 Quality appraisal of studies included for the narrative meta‐synthesis

Only six qualitative intervention articles were considered high quality and included in the findings. These six articles focus on bilingual/multilingual education in Peru (Neugebauer & Currie‐Rubin, 2009), curriculum in Jamaica (Roofe, 2014), parental and community participation in Argentina (Stein & Rosemberg, 2012), general pedagogical strategies in Colombia and the U.S. Virgin Islands (Gonzalez et al., 2013; Mahurt, 1993), and teacher training in the Caribbean (Warrican et al. 2008).

Only 14 qualitative nonintervention articles were considered high quality and included in the findings. These studies focused on: assessment in multiple countries (Leal Carretero & Suro Sánchez, 2012); pedagogical approaches in Brazil, Mexico, and Puerto Rico (Gómez Nashiki, 2008; Medina & Costa, 2013; Ribeiro & Souza, 2012; Rosado & Campelo, 2011); parental and community participation in Jamaica and Puerto Rico (Kinkhead‐Clark, 2014; Volk & de Acosta, 2001, 2003); bilingual/multilingual education in Colombia (Guevara & Ordoñez, 2012); reading skills in Argentina (Manrique & Borzone, 2010); teaching practices for reading in Jamaica, Mexico, and Argentina (Diuk, 2007; Jiménez et al., 2003; Webster, 2009); and literacy acquisition among deaf students (Massone & Baez, 2009).

#### 7.4.1 Research design

We discuss the quality of the qualitative intervention research in this section through a summary and analysis of the research designs, ethics, and reflexivity, and the relevance of the research to the field.

#### 7.4.2 Statement of research

A clear statement of purpose forms the basis for how a researcher decides on methods, measurement, and analysis of a problem (Ford, 2009). Our review assumes the purpose of the research, or problem statement, “may be phrased as statements of research purpose, as specific research questions, or as research hypotheses, depending on the purpose of the study and selected design” (McMillan & Schumacher, 2001, p. 86). A research statement serves to introduce the reader to the research, provide context, and create a framework in which to report results that in the end guide the entire exercise (Bryman, 2007). We rated the quality of the research statement on the following parameters:

**Table:** 

**Quality review criteria**
Clear statement of research – The goal of the research – Why it is important

##### 7.4.2.1 Qualitative intervention

Reviewers rated the clarity of the stated goals as “high” on all six articles when both the goal and the methods by which the goal will be realized are clearly stated in the text. Successful research statements also justify goals by explaining their importance. In comparison, weak goals are not clearly articulated or contradict other portions of the text. For example, Mahurt (1993) did not include an explanation of the programs they are evaluating anywhere in the text.

Effective statements of importance not only explain why the research is necessary but also show why findings would be important within the research context as well as within the larger community of stakeholders. Neugebauer and Currie‐Rubin (2009) successfully demonstrate the importance of their research in Peru through the following statement:

The need for research focused on read‐alouds in such communities is particularly compelling given the nature of read‐aloud pedagogy (the integration of oral elaborations of text and vocabulary with written narratives) and the tradition of oral story telling that is central to many indigenous cultures. Given the strong emphasis in these communities on oral histories as a means of “communicat[ing] ideas and images” (Mello, 2001, p.1), read‐alouds can extend the connection between oral narratives and written genres. Furthermore, this instructional format includes community experiences and simultaneously provides a wealth of language‐rich pedagogy especially useful for bilingual populations (p. 297).

In this passage, Neugebauer and Currie‐Rubin (2009) explain the relevance of the research for the local communities as well as how the research would be applicable to the larger field, particularly bilingual populations. Of the surveyed articles, the majority communicated the importance of their stated goals.

##### 7.4.2.2 Qualitative nonintervention

Nearly all qualitative nonintervention articles clearly stated the goal of the research. Reviewers rated the quality of 11 articles as “high” and three articles as “medium” quality on the clarity of the research goals. The articles where quality was rated high clearly stated the goal and wove the goal throughout the article. Articles, where quality was rated as low, did not clearly state their goal or did not weave the goal throughout the article.

The majority of nonintervention articles also effectively communicated the importance of the stated goal. Reviewers rated 13 of the 14 articles as either “high” or “medium” quality for demonstrating the importance of the research goals. Articles rated as high quality showed importance by highlighting gaps in the existing literature or situating the research within continuing challenges to EGL. For example, Manrique and Borzone (2010) argue that their research in Argentina is necessary because the existing literature does not explain the difficulties that children from marginalized sectors have in processing process written narratives. Refer to Table E1 in Appendix E for quality ratings of research statements for all qualitative studies.

#### 7.4.3 Methodology

We assessed the quality of the papers' methodologies to the extent they were described using the criteria below:

**Table:** 

Quality review criteria
● Appropriateness of qualitative methodology – Does the research interpret or illuminate the actions and/or subjective experiences of research participants? ● Research design addresses the aims of the research – Is the research guided by research questions or hypotheses? – Has the researcher justified the research design? (i.e., have they discussed how they decided which methods to use)?

##### 7.4.3.1 Qualitative intervention

Reviewers rated two qualitative intervention articles as “high,” one article as “medium,” and two as “low” quality on including research questions or a hypothesis, while one article did not clearly identify the research questions or hypothesis. In articles that included strong research questions or hypotheses, the research questions or hypotheses were explicitly stated in the text and guided the overall research. In comparison, low performing articles included research questions that were not well formulated or did not align with the data researchers collected.

The majority of included studies failed to explicitly convey the methodologies used in the research. Two articles scored high, one scored medium and three scored low quality. Strong articles clearly articulated the methodology including the methods used, rationale for using particular methods, and an explanation of how the researchers used the methodologies. Surveyed research papers used a variety of methodologies including observations, case studies, qualitative interviews, and journaling. Overall, only one study altered the methods during the evaluation to reflect more of a case study format. The other studies (*n* = 5) did not report any modification to the methods.

Most of the surveyed papers adequately justified the use of qualitative methods. Reviewers rated four articles as “high,” one article as “medium” and one as not mentioned on appropriateness of qualitative methodology and research design. Compelling justifications explained how the research aimed to achieve its goals through an understanding of the subjective experiences of teachers and students. For example, Mahurt (1993) used a case study to provide intensive, in‐depth exploration using a hermeneutic phenomenology theoretical framework. However, only three of the six articles scored high on research methodology justification. The other articles did not explain how methodologies were used and why. For example, the article by Roofe (2014) does not explain why focus group discussion or semistructured interviews were chosen or why the study was limited to only 11 teachers.

##### 7.4.3.2 Qualitative nonintervention

More than half of qualitative nonintervention articles used clearly stated research questions to guide the text. For example, Webster (2009) states, “What is the influence of teacher read‐alouds of informational texts on grade 1 students' science learning as revealed through their drawings and written retellings?” (p. 663). Other articles either included vague research questions embedded in the text, used exploratory research designs that do not necessarily require research questions, or did not include research questions.

Qualitative nonintervention articles successfully supported the use of qualitative methodologies but could provide greater detail to justify the use of specific methods. The majority of surveyed articles (*n* = 13 of 14) effectively used qualitative research to illuminate the actions and subjective experiences of the research participants. The articles included a variety of subjective experiences and perspectives including students' interactions, reactions to particular texts, and perspectives on curricula as well as teachers' actions, goals, reflections, and perspectives on curricula. However, a minority of articles (*n* = 6) explicitly stated the research methodologies used in their respective studies, and none of the surveyed articles discussed modifying their methods. Furthermore, eight articles either included an incomplete discussion or explanation of why particular methods were chosen (Kinkhead‐Clark, 2014), lacked theoretical support for the chosen design (Rosado & Campelo, 2011), or included no explanations of the methodological choices (Ribeiro & Souza, 2012). Similarly, only 10 of the surveyed articles included justifications for why particular methods were best positioned for particular goals and contexts, and none of the articles explained how researchers triangulated multiple methodologies. Refer to Tables E1 and E2 in Appendix E for quality ratings of methodologies for all qualitative studies.

#### 7.4.4 Data

Describing methodologies also entails detailing the setting, justification, process, and the form of data collected. Reviewers accounted for the following elements when rating a study on data quality:

**Table:** 

Quality review criteria
● Was the data collected in a way that addressed the research issue? – If the setting for data collection was justified – If it is clear how data were collected (e.g., focus group, semistructured interview, etc.) – If the researcher has justified the methods chosen – If the researcher has made the methods explicit (e.g., for interview method, is there an indication of how interviews were conducted, did they used a topic guide?) – If methods were modified during the study. If so, has the researcher explained how and why? – If the form of data is clear (e.g., tape recordings, video material, notes, etc.) – If the researcher has discussed saturation of data

##### 7.4.4.1 Qualitative intervention

Evaluators rated three of the qualitative intervention articles as “high” and two as “medium” on presenting details of data collection. Articles rated as medium did not present data collection protocols or articulate the length or timing of data collection. Although all articles touched on the data collection setting, only three described the data collection context. Articles rated as low on this measure did not explain the importance of the site or include a justification for why a particular site is most relevant for the evaluation. Finally, none of the articles included a discussion of data saturation; this discussion may have helped the reader understand cases such as in the study of Roofe (2014) in Jamaica, which included only 11 interviews. This number of interviews could have been sufficient for the study, but a discussion of saturation or selection process would strengthen the article's scientific validity.

##### 7.4.4.2 Qualitative nonintervention

Of the 14 articles reviewed, 11 effectively justified and explained the data collection site. For example, Kinkhead‐Clark (2014) selected the Turtle Islands because it is a diverse cultural setting that offers insight into the role of culture in literacy. Furthermore, the researcher was a teacher in the selected classroom, which allowed her to have increased access to the student participants (Kinkhead‐Clark, 2014). Articles that include weaker explanations of the data collection site lack sufficient detail (Gómez Nashiki, 2008; Rosado & Campelo, 2011). For instance, Rosado and Campelo (2011) state that data collection took place in a school because the research required the study to take place in a school. However, the researchers did not provide a justification for why particular research schools were selected.

Similarly, the majority of surveyed articles successfully described the type and form of collected data. Ten of the 14 articles described the form of data and 11 also described how researchers collected the data. Although articles rated as “low” quality often lacked details of the data collection process, strong articles included clear descriptions of how researchers collected the data as well as the type of data collected. For instance, Volk and de Acosta (2001) state:

From January through to the end of the school year, we observed and audio taped in the classroom twice a month for the three‐hour morning session and for about an hour after lunch; times when most literacy events occurred. We observed and taped in each home once a month for between two and four hours at a time. Observations and interviews were conducted in two of the churches and their Sunday schools; interview data were collected about the other church and Sunday school (p. 197).

Finally, although many of the qualitative nonintervention articles effectively described the data researchers used as the foundation for the analysis and findings, none of the surveyed articles discussed data saturation. Refer to Table E3 in Appendix E for quality ratings of data collection for all qualitative studies.

#### 7.4.5 Data analysis

We reviewed the quality of qualitative data analysis for the included articles on the following criteria:

**Table:** 

Quality review criteria
● Was the data analysis sufficiently rigorous? – If there is a thorough description of the analysis process – If thematic analysis is used. If so, is it clear how the categories/themes were derived from the data? – If the researcher explains how the data presented were selected from the original sample to demonstrate the analysis process (e.g., I chose this because 90% of the participants said something similar) – If sufficient data are presented to support the findings – If contradictory data are taken into account – Whether the researcher critically examined their own role, potential bias, and influence during analysis and selection of data for presentation – If the researcher considered contextual factors that may have influenced the research results (if you do a study in Peru, you must take into consideration context of Peru, Urban vs. Rural, etc.)

##### 7.4.5.1 Qualitative intervention

Out of six articles, only two articles received high ratings for their description of the analysis process, while four did not discuss this process in detail. Three articles used thematic analysis and of these three, two used thematic analysis effectively—that is, the articles used themes to guide the analysis process and supported these themes with data. Five of the six articles used sufficient data in their analysis; however “sufficient” is dependent on the parameters of the research study. For example, Mahurt (1993) used limited but sufficient data sources because the research aimed to look at the struggle of a single teacher trying to enact behavior change. Furthermore, only one article explained how researchers selected the data presented in the article from all of the collected data and only two articles included discussions of contradictory data. Contradictory, minority results are important to note to demonstrate that all findings are taken into account. Failing to report contradictory results may be an indication for a bias in the research findings. Four of the six articles included a consideration of the context in their analysis. For example, the article “Orality, Literacy and Reading: Differences and Complexities Facing the Public School” highlights the importance of context through its description of other development programs in the area including the Ler e Escrivir project. Context is important to consider in this case because some of the changes described in the article could have been a result of the other intervention.

##### 7.4.5.2 Qualitative nonintervention

The qualitative nonintervention articles could improve the description and execution of the data analysis. More than half of the articles (*n* = 10) included a thorough description of the data analysis process. Thorough descriptions explicitly stated the relevant analytical process in sufficient detail for the reader to understand how researchers translated data into findings. For example, Leal Carretero and Suro Sánchez (2012) described their analysis by presenting a comparative table with the characteristics of the tests given to participants then followed up with a categorical analysis (pp. 738–739) in their study on literacy assessments from multiple countries. Although 14 articles reported using thematic analysis, only seven of those did so effectively. Furthermore, 10 of the surveyed articles used sufficient data in their analysis process but only two articles presented data to demonstrate the analysis process. Lower performing articles included analyses that are hard to follow (Medina & Costa, 2013) or lack sufficient detail (Gómez Nashiki, 2008; Guevara & Ordoñez, 2012).

The qualitative nonintervention articles failed to adequately report the limitations and context of the data used in the analysis. Nine articles included some mention of the research context. However, only five articles included a discussion of how researchers' bias may have affected the data analysis process. These articles positioned the research within the analytical process, stating how their background may predisposition them to particular findings. Six articles included a weak discussion of researcher bias, and the remaining three articles did not discuss potential biases in the analysis process. Finally, three of the 14 articles presented information regarding the consideration of contradictory data. Refer to Tables E4 and E5 in Appendix E for quality ratings of data analysis for all qualitative studies.

#### 7.4.6 Statement of findings

We rated articles' statements of findings on these parameters:

**Table:** 

Quality review criteria
● Is there a clear statement of findings? – If the findings are explicit – If there is adequate discussion of the evidence both for and against the researcher's interpretations – If the researcher has discussed the credibility of their findings (e.g., triangulation, respondent validation, more than one analyst) – If the findings are discussed in relation to the original research questions

##### 7.4.6.1 Qualitative intervention

The majority of the selected papers clearly presented findings, but they could have provided more information about how researchers arrived at the findings. Three articles discussed findings in relation to their original research questions or the findings were in direct conversation with them and three did not discuss their findings in terms of the research questions. The majority of articles did not include a discussion of triangulation, respondent validation, multiple analysts, or evidence against interpretations. Only one article included evidence that contradicted the findings of the research. Furthermore, two articles did discuss credibility; one article used the qualitative research to supplement the quantitative research findings (Neugebauer & Currie‐Rubin, 2009) and another triangulated results through multiple qualitative methods (Mahurt, 1993).

##### 7.4.6.2 Qualitative nonintervention

The qualitative nonintervention articles successfully communicated findings but could bolster the credibility of findings through triangulation and the presentation of contradictory data. The reviewers rated seven articles as “high,” six as “medium,” on explicitly stating findings, and one article did not include a clear statement of findings. Articles rated as high clearly articulated findings that linked to the research questions, theoretical framework, context, and analysis (Manrique & Borzone, 2010; Webster, 2009). Although a minority of the articles (*n* = 6) linked findings to the original research questions, this type of presentation improves the organization and flow of the text for the reader (Guevara & Ordoñez, 2012; Medina & Costa, 2013; Volk & de Acosta, 2001; Webster, 2009). Only three articles discussed evidence against the findings and only four discussed triangulation. Articles rated as high typically triangulated findings using multiple data sources (Medina & Costa, 2013), or multiple researchers (Jiménez et al., 2003). For example, Webster (2009) triangulates her findings between the students, the teacher, her observations, and observations of the assistant principal. Refer to Tables E6 in Appendix E for quality ratings of findings statements for all qualitative studies.

#### 7.4.7 Ethics and reflexivity

Reviewers assessed the quality of an article's transparency on ethics based on its described recruitment strategy, its recognition of potential bias in the researcher‐participant relationship, and its attention to protection of human subjects in research.

#### 7.4.8 Recruitment strategy

We evaluated studies' recruitment strategy on two criteria:

**Table:** 

Quality review criteria
Appropriate recruitment strategy – If the researcher has explained how the participants were selected – If they explained why the participants they selected were the most appropriate to provide access to the type of knowledge sought by the study

##### 7.4.8.1 Qualitative intervention

The qualitative intervention articles included limited information on recruitment strategies. Five out of six articles described how participants were selected. For example, Mahurt (1993) clearly states that participant selection was based on the following criteria:

(a) a teacher who had made a recent decision to change to whole language; (b) a teacher whose decision to change was based on personal factors and not influences from graduate courses or mandates from the school district or administrator; (c) a teacher who seemed interested enough in whole language instruction to continue for at least two years (p. 8).

Furthermore, four out of six articles explained why researchers selected certain participants over other individuals.

##### 7.4.8.2 Qualitative nonintervention

Nine of the qualitative nonintervention articles included an explanation of how researchers selected participants. Volk and de Acosta (2003) explained that they chose to include three children in their study in Puerto Rico to balance the need for rich description of a variety of literacy experiences with the constraints of equipment and time. Furthermore, the researchers selected participants in consultation with their teacher and based on information from observations, an assessment conducted by the teacher, and an informal reading assessment. Thus, the researchers demonstrated the process used for selection as well as what type of criteria were involved. However, the majority of articles included an insufficient explanation of the method used to identify the study population (e.g., Kinkhead‐Clark, 2014; Rosado & Campelo, 2011). Furthermore, the majority of articles (*n* = 11) did not include an explanation of why particular participants were chosen over other participants.

#### 7.4.9 Research‐participant relationship

We evaluated the assessment of researcher‐participant bias using the following criteria:

**Table:** 

Quality review criteria
● Has the relationship between the researcher and participants been adequately considered? – Consider if the researcher critically examined their own role, potential bias, and influence during: (i) Formulation of research questions and research instruments (e.g., asking leading questions) (ii) Data collection, including sample recruitment and choice of location

##### 7.4.9.1 Qualitative intervention

Only one article included a discussion of subjectivity and positionality in the formulation of research questions. The remaining articles did not acknowledge how researchers' bias may affect the formulation of research questions or instruments or how researchers' involvement in “interpreting” questions for participants may have led the participants to a certain answer. Further, only one article mentioned the potential for researcher bias in the data collection process.

##### 7.4.9.2 Qualitative nonintervention

The majority of articles that touched on potential biases focused on how researchers influenced the site selection, while a small number of articles discussed researchers' bias in the sampling and recruitment of participants (Jiménez et al., 2003; Kinkhead‐Clark, 2014; Medina & Costa, 2013; Webster, 2009). Only seven of the articles discussed the researchers' bias in the data collection process. Bias can influence a number of factors during data collection including sampling, recruitment, and site selection. Eight of the articles included a discussion of the researchers' bias in the formulation of research questions. In “Teaching English to Very Young Learners,” the researchers disagreed with the school's early introduction of English as a second language, a concept which they are aiming to better understand. This bias was crucial to present within the text as the authors cannot fully remove this bias from their analysis. However, many articles did not present any information about how the researchers' bias may have affected the various research components. Finally, the majority of articles did not mention any bias in the data analysis process and only five included a discussion of subjectivity or positionality.

#### 7.4.10 Ethics

Although there is no overarching ethical review board covering the entire LAC region, individual institutions, universities, and publications have their own ethical review boards and ethics codes with similar standards that researchers should follow. As a standard protection for human subjects, the CASP qualitative research checklist recommends assessing ethics on the following dimensions:

**Table:** 

Quality review criteria
● Have ethical issues been taken into consideration? – If there are sufficient details of how the research was explained to participants for the reader to assess whether ethical standards were maintained – If the researcher has discussed issues raised by the study on sensitive issues (e.g., issues around informed consent or confidentiality or how they have handled the effects of the study on the participants during and after the study) – If approval has been sought from an ethics committee

##### 7.4.10.1 Qualitative intervention

None of the articles included a description of how researchers explained the study to participants, any reference to working with an institutional review board (IRB) or seeking ethical approval, or a discussion of sensitive issues raised by the study. Ethical standards serve the critical role of protecting informants, particularly vulnerable informants such as children. We recognize, however, that reporting standards vary greatly by field such that an economics journal, for example, might not require any mention of ethical procedures whereas a medical journal would surely require it. Thus, although several of the studies do not report on seeking ethical approval, this does not necessarily mean that they did not obtain it.

##### 7.4.10.2 Qualitative nonintervention

As with the intervention articles, qualitative nonintervention articles included only limited discussions of ethical issues related to the research. Only two of the surveyed articles mentioned obtaining consent from participants and only one article mentioned conducting research through an IRB. The vast majority of articles made no reference to ethical approval or issues of consent. Furthermore, none of the articles included a discussion of how researchers dealt with sensitive issues or took precautions to ensure the well‐being and security of participants. Most of these studies did not cover data that would be considered highly sensitive, although many did work with children, who are considered a vulnerable population. Because most of the reviewed articles did not report on how ethical issues were addressed, it is difficult to say whether or not researchers took into account ethical considerations and to what extent. These procedures are sometimes not reported on in publications because they are so standard that it is assumed that one has completed them. In addition, researchers would not have been required to undergo IRB approvals for some of these studies as they made use of publicly available secondary data sets.

#### 7.4.11 Relevance to the field

Finally, raters reviewed qualitative intervention and nonintervention articles for their relevance to the field based on the following criteria:

**Table:** 

Quality review criteria
● How valuable is the research? – If the researcher discusses the contribution the study makes to existing knowledge or understanding (e.g., do they consider the findings in relation to current policy or relevant research‐based literature?) – If they identify new areas where research is necessary – If the researchers have discussed whether or how the findings can be transferred to other populations or considered other ways the research may be used

##### 7.4.11.1 Qualitative intervention

Reviewers rated two of the six qualitative intervention articles as “high” on communicating the value of the research and two as “medium.” The other two articles did not effectively contextualize findings within the existing literature or explicitly state the relevance to readers or the larger field. For example, although Stein and Rosemberg (2012) do not discuss how the study contributes to existing knowledge or understanding, they do discuss how this research could speak to existing theory around students' learning to write in English. Another way to communicate relevance is through a discussion of how the research can be applied in other contexts. Only two articles included this discussion, while four did not.

Finally, the majority of articles did not identify areas for further research. The two articles that effectively communicated areas for new research suggested expanding the current study (Mahurt, 1993) and continuing research on read‐aloud efficacy in international contexts (Neugebauer & Currie‐Rubin, 2009). However, two articles did not discuss areas for further research and two articles discuss additional research topics in an unclear manner.

##### 7.4.11.2 Qualitative nonintervention

Overall, the qualitative nonintervention articles consistently situated the research within the existing literature and intellectual field. The articles discussed the contribution to existing knowledge, identification of areas for further research, and how the findings could be used. Articles contribute to existing knowledge by supporting existing claims, expanding on existing research, or filling in gaps in the current literature. Ten articles discussed how the findings contributed to existing knowledge, including both existing literature and education policies. For example, Volk and de Acosta (2001) state,

Previous research has emphasized matches and mismatches between teaching and learning practices in homes and classrooms. Often, mismatches are identified as causes or correlates of the low achievement levels of children who come from diverse cultures. But while continuity is an admirable goal, the complex and shifting relationships between literacy practices in these three homes and in this bilingual classroom suggest that an analysis limited to matches and mismatches is oversimplified and misleading. A broader view of literacy that encompasses many literacies that are similar in some ways and different in others may be more appropriate and, ultimately, more useful for teachers (p. 220).

In contrast, very few articles suggested areas for further research. In fact, the majority of articles (*n* = 10) did not include any mention of areas for further research.

#### 7.4.12 Replicability

We assessed replicability based on two dimensions: first, whether stakeholders could replicate the program; and second, whether researchers provided sufficient information for other researchers to replicate the study in different contexts. Typically, systematic reviews with an emphasis on qualitative research assess replicability only on the research design dimension; however, given the context of our review and the end‐users, we also assessed replicability of the program so that stakeholders could independently consider whether example programs may fit their particular context and adapt the program to improve implementation. We used the following criteria to assess replicability:

**Table:** 

Quality review criteria
● Information for stakeholders to assess replicability – Does the paper provide adequate details on the design and implementation of the intervention to enable replication, such as: (i) Length of training (ii) Monitoring tools (iii) Training materials

##### 7.4.12.1 Qualitative intervention

Only two articles provided enough information to repeat the described studies. Neugebauer and Currie‐Rubin (2009) explained exactly how each of the seven techniques described in their article were used and could be easily adapted and used in the classroom. Furthermore, Gonzalez et al. (2013) provided descriptions of the types of collaborative learning strategies researchers implemented in the study classroom; however, there were no explicit statements about the length of the training, the tools or instructional methods used, or the training materials for teachers to be able to implement the methods.

Similarly, a study's replicability depends on whether the researcher includes adequate details on the study design, including much of the quality criteria we previously discussed. Based on our assessment of the prior dimensions of the quality review, the majority of articles did not include enough information to easily replicate the studies that were discussed. Many articles were strong in some dimensions of quality, but these same articles excluded other elements that would be essential for replication. For example, two of the articles do not present methodological protocols, explanations of how methods were actually implemented, nor training materials (Mahurt, 1993; Stein & Rosemberg, 2012).

##### 7.4.12.2 Qualitative nonintervention

None of the qualitative nonintervention articles discussed how findings can be transferred to other populations or used in other ways. Reviewers rated four articles as high, one as medium, three articles as “low,” two as “not applicable” (as they were ethnographic studies), and four articles that did not include any information about the transferability of findings. Volk and de Acosta (2001) discussed how findings could be used to improve teacher practices, Jiménez et al. (2003) discussed the implications of the research, and Guevara and Ordoñez (2012) discussed how their findings might be relatable to similar contexts. For example, Guevara and Ordoñez (2012) offered the following advice for bilingual schools in other monolingual contexts:

It is also essential that children always understand what they are doing and saying in the foreign language and that they also do it in Spanish. The effective, conscious use of the students' knowledge of their first language is a must in helping our monolingual children become good consecutive bilinguals; and a truly bilingual curriculum may be a much better way than what we know as bilingual education to work towards bilingualism at school in monolingual environments (p. 22).

Examples of how the findings can be applied in different contexts help make the findings relevant to practitioners in the region. Refer to Table E6 in Appendix E for quality ratings of relevance and replicability for all qualitative studies.

##### 7.4.12.3 Quality appraisal correlational studies

Systematic reviews typically do not include quantitative nonintervention studies because often these studies are not able to address counterfactual questions. We considered it important to include these studies, however, because they often examine the specifics of reading acquisition mechanisms and trajectories. In addition, these studies are able to uncover predictors of reading success, as part of the larger story of evidence of EGL development in the LAC region. In particular, we believe these studies can guide curricular and standards development, entangle specific aspects—and paths through which—a “bundled” EGL program may impact reading and help develop more targeted, language‐ and country‐specific reading measures.

The quantitative nonintervention studies comprised the largest number of studies in the systematic review. The review included 61 articles from the following countries: Brazil (*N* = 19), Mexico (*N* = 13), Chile (*N* = 10), Argentina (*N* = 6), Peru (*N* = 4), Guatemala (*N* = 3), Cuba (*N* = 2), Puerto Rico (*N* = 1), Colombia (*N* = 1), and Costa Rica (*N* = 1). We also included two studies that involved cross‐country comparisons. The included studies were mostly from psychology and linguistics disciplines and covered a range of topics on predictors of reading skill development in the LAC region.

#### 7.4.13 Quality criteria

All nonintervention studies were rated by reviewers on pooled questions to target the following categories of quality: outcome measures, sample, data collection, data analyses, and external validity. In the following section, we first describe how the whole set of studies were reviewed per category; in the second part, we present reviewers' ratings for each study on each category.

##### 7.4.13.1 Outcome measures

Our most important category was whether or not reading, writing, or some reading‐ or writing‐related subskill was measured. Two main questions were used to determine whether a study was included or not:

(1) Did the outcome measure include some measure of reading or a reading subskill (e.g., fluency, PA, language decoding, letter knowledge, comprehension, etc.)?
(2) If the study did not include a measurement of reading or a reading subskill, was literacy measured in a different manner?

In the sample, 57 of 61 studies had an outcome measure of reading or a reading subskill. In general, PA and reading were measured. Reading measures ranged from word level reading to reading connected text. One example of a study that focused on the essential components of reading and included writing was Plana and Fumagalli (2013). In contrast, some studies focused only on decoding (Jaichenco & Wilson, 2013). One study in the sample measured reading in a different manner than through PA or reading comprehension. Silva et al. (2014) measured students' narrative skills using a wordless picture book that students used to construct a story.

The majority of the studies used reading assessment tests to measure reading outcomes, which reduces the risk of measurement error. However, it is important to note that reviewing the validity of each of the assessments reported on in this study was not included in the original protocol and, therefore, results from these assessments must be interpreted accordingly. Only six of 61 studies in the sample reported information on self‐reports. These involved student (Cervini, 2015), parent (Salazar‐Reyes & Vega‐Pérez, 2013), or teacher surveys (Janus, 2011).

We were also interested in understanding whether the studies provided information on data collector training to determine, to the extent reported, whether there were any concerns regarding the independence of the observers. We found that only 13 of 61 studies provided information on training of test administrators. Test administrators mainly consisted of the study author (De Abreu & Cardoso‐Martins, 1998) and graduate students (Benitez & Flores, 2002). In one study, research assistants were trained over a week‐long period on how to record their classroom observations. They then practiced by observing videotaped and live classrooms in Northeast Brazil. Following this training, pairs of observers were sent to 17 different classrooms in a school to obtain interrater reliability (Fuller et al., 1999). The studies demonstrate a wide range of variability when it comes to data collector training procedures and the degree to which such procedures are reported.

##### 7.4.13.2 Sample

We assessed whether the sample selection criteria were provided to determine whether the sample was appropriate for addressing the research question and to assess the generalizability of the results. We found that 45 of 61 studies provided sample selection criteria or justification of the sample selection process. Samples were generally described by age, grade, gender, economic level, country, and geographical region. In some cases, samples were also described as attending private or public schools (Jiménez, Puente, Alvarado, & Arrebillaga, 2009). Some studies excluded students with visual or hearing impairments (Salles & Parente, 2002), while others included students with hearing impairments (Bandini et al. 2006). One study included students from 16 Latin American countries for a total sample of 90,251 students (Torrecilla & Carrasco, 2014). This study examined the effect of child labor on third‐ and sixth‐grade students' academic achievement in math and reading. Another study compared students from Latin America to students in the United States (Treiman, Kessler, & Pollo, 2006).

##### 7.4.13.3 Data collection

We determined the quality of various aspects of data collection, including training test administrators, data collection procedures, and whether or not the study took into consideration potential data collection implementation failures. Given that we had to rely upon study authors to report this information, we were cautious in interpreting these results. In other words, simply because it was not reported does not mean it was not done.

In the sample, 31 studies reported on data collection procedures. These ranged from individual to group administration of tests in the classroom or another room in the school. Locations were generally described as quiet. One study reported that children were individually tested in a single session in a quiet room in the school (Treiman et al. 2006). Another study reported that the students were tested using a web‐based assessment (Rosas et al., 2015). Nearly half of the studies in the sample did not report the data collection procedures.

Only 10 studies in the sample reported considering data collection failures, for a number of reasons, including priming effects and blinding (Silva et al., 2014) and inability to locate all of the participants (Castro, Lubker, Bryant, & Skinner, 2002). Another reason given for potential data collection errors was the cultural and linguistic differences between the test administrator and the students (Kudo & Bazan, 2009) and lack of cultural appropriateness (Castro et al., 2002). Castro et al. (2002) used a test that was translated and previously used in a United States study. The researchers concluded that it might have lacked cultural appropriateness.

Finally, only nine studies in the sample mentioned that monitoring can influence behavior. Monitoring behavior was not a factor across the studies. The focus of the studies was test performance. Students were assessed either orally or in a written test. In general, no information was provided regarding the behavior of the child while reading. The focus was on the accuracy of test responses, not on the effects of being administered an oral assessment or the effect of students' behavior due to testing.

##### 7.4.13.4 Analysis

The analysis section for each study was important in determining the quality of the entire study. We asked the following questions to determine the quality of the analysis section:

(1) Is there a description of the analytic method(s) used?
  The majority of the studies in the sample, 55 of 61, gave a strong description of the analysis methods used. Some studies provided ample description of the statistical analyses conducted (Páez et al., 2007) while others gave brief descriptions and used simple analyses such as histograms (Bandini et al., 2006). One study did not provide a description of the analysis (Melchiori et al. 2012).
(2) Does the study justify the analysis method (is the analysis method appropriate for the research question/objective)?
  In the sample of studies, 44 of 61 studies used analysis methods that were appropriate for the research question or study objective. In some cases, the analysis method was considered to be too simplistic and did not necessarily yield empirical information. For example, Dias et al. (2006) used *T* tests for analyses and Morales et al. (2013) used differential item functioning.
(3) Were any participants not included in the analysis? If so, is there justification for why?
  In 43 of 61 studies in the sample, all students were tested. Of the studies that excluded students from the sample, reasons provided were that they were absent (Cardoso‐Martins & Da Silva, 2010), researcher error (De Abreu & Cardoso‐Martins, 1998), or because of age (Rindermann et al., 2014). Ten studies in the sample did not specify this information in their report. The absence of these students may have resulted in a bias in the empirical findings.
(4) Was there data reported on covariates?
  Information on covariates was reported in 35 of 61 studies in the sample. Covariates centered on similar characteristics mentioned above for sample descriptions (e.g., age, grade, gender, economic level, country, and geographical region). However, some studies included covariates such as parent's educational levels (Hoddinott et al., 2013; Muñoz, 2002) and sociocultural characteristics influencing students (Iparraguirre, 2014).
(5) Are there appropriate reliability scores for all tests?
  In the sample, 18 of 61 studies reported reliability scores for the tests. Among the studies reporting test scores, Cronbach's *α* was commonly used to calculate reliability scores (Jiménez et al., 2009; Páez et al., 2007). Those studies with tests with reasonable reliability scores were deemed high quality.

##### 7.4.13.5 External validity

We aimed to determine whether authors generalized their findings only to the relevant population of study. In the sample, 47 of the 61 studies generalized the study outcomes to the population in the study. Several studies generalized the study findings to a different grade level or age group (Ramírez, Verdugo, & Sánchez, 2000), another country (de Manrique & Signorini, 1994), or to the population in the study despite a small sample (Bandini et al., 2006). Still, others generalized to the entire population in the country (De Abreu, & Cardoso‐Martins, 1998) and across countries (Abadzi, Crouch, Echegaray, Pasco, & Sampe, 2005). As such, most studies generalized their findings to a relevant population.

In the second part of the analysis, a quality rating of “High,” “Medium,” and “Low” was assigned for each study on each category. Reviewers assigned ratings as they answered the questions above. If the answer to the question was “Yes” and the reviewer could identify portions of the full‐text study that could justify their answer, the study was rated as “High,” and vice versa for “Low.” Reviewers rated studies as “Medium” on categories that were present, but were not strongly backed up in the study.

Two important points emerged in this part of the analyses. First, the notion of an appropriate “theoretical framework” may have been conceptualized slightly different among the reviewers from different disciplinary backgrounds, and therefore, studies with Medium‐ or Low‐quality theoretical frameworks were rechecked by a second reviewer. Second, in terms of quality of data collection procedures, the procedures under which data collection took place (i.e. whether it is was in a quiet room, whether testing was counterbalanced, whether fatigue effects were taken into consideration etc.) were of more importance to these kinds of nonintervention studies, as opposed to observer bias, because there is a lower likelihood of bias due to the fact there are no programs to have any vested interest in.

### 7.5 Quantitative data analysis

This section presents results from the meta‐analysis and narrative review of the effects of different types of programs on reading outcomes. We present a separate analysis for each of the program types that were evaluated in the primary studies, including teacher training programs, technology in education programs, school feeding and other nutrition programs, school governance programs, programs with an emphasis on teacher practices, and programs with an emphasis on parental involvement.

To synthesize the findings for each intervention type, we first conducted a meta‐analysis for each of the RCTs, followed by a meta‐analysis for each of the nonexperimental studies, and a meta‐analysis that pools the RCTs and nonexperimental studies.

#### 7.5.1 Impact of teacher training programs

Of the included studies, four presented an estimate of the impact of teacher training programs on reading outcomes. Of these studies, we were able to include two studies in our meta‐analysis (Pallante and Kim, 2013; Yoshikawa et al., 2015). We did not include the other two studies because they evaluated the same program in Chile (Gomez Franco et al., 2014; Mendive, Weiland, Yoshikawa, & Snow, 2016) as Yoshikawa et al. (2015) and were rated as having a higher risk of selection bias. We summarize the evaluations that focused on the impact of teacher training in Table 5. This table also summarizes the outcome measures and the evaluation design that were used in the primary study. Despite the small number of studies, we still include a meta‐analysis on the effects of teacher training programs on reading outcomes because both studies are RCTs with a low risk of selection bias in a very similar context.

**Table 5:** Primary studies that focus on the impact of teacher training

Studies	Definition of outcome variable(s)	Evaluation design	Included in meta‐analysis?	Country
Gomez Franco (2014)	Vocabulary acquisition	Cluster RCT	No	Chile
	Reading comprehension			
Mendive et al. (2016)	Language test score	Cluster RCT	No	Chile
	Early literacy outcomes			
Pallante and Kim (2013)	Letter naming	Cluster RCT	Yes	Chile
	Word reading			
	Vocabulary acquisition			
	Phonemic segmentation			
Yoshikawa et al. (2015)	Language test score	Cluster RCT	Yes	Chile
	Early literacy outcomes			

Abbreviation: RCT, randomized controlled trial.

##### 7.5.1.1 Meta‐analysis for RCTs

**Figure 6:**
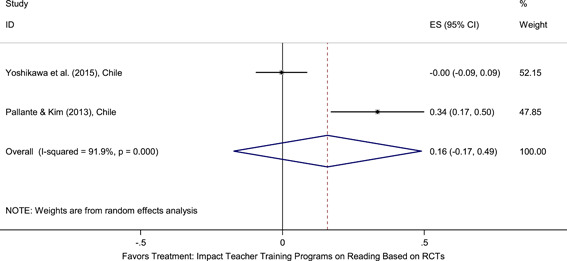
Impact of teacher training programs on reading outcomes. CI, confidence interval; RCT, randomized controlled trial

The results of the meta‐analysis for the RCTs are presented in Figure 6. We found no evidence that, on average, teacher training had a positive effect on reading outcomes (SMD = 0.16, 95% CI = −0.17, 0.48; evidence from two studies). However, Pallante and Kim (2013) found a medium‐sized, positive, and statistically significant effect on the reading outcomes of students in kindergarten and first grade in their evaluation of a teacher training program in Chile that targets PA, alphabetics and phonics, fluency, vocabulary, reading comprehension, and writing. This was a comprehensive teacher training program that also included a focus on coaching and sustained follow‐up. In contrast, Yoshikawa et al. (2015) did not find positive effects of a teacher training program for teachers in prekindergarten classrooms in Chile. They did find positive impacts for emotional and instructional support of teachers, but the results suggested that these behavioral changes did not translate to positive effects on EGL outcomes. However, Mendive et al. (2016) demonstrated that the lack of positive effects on reading outcomes may have resulted from problems in the implementation of the program. It is possible that teacher training programs need to be comprehensive and complemented by coaching and sustained follow‐up in order to have positive impacts on reading outcomes. The coaching and sustained follow‐up could result in improvements in the fidelity of implementation.

At the same time, however, we need to be careful in how we interpret the results because we only encountered two studies, which were both implemented in Chile. The effects of teacher training programs may well be different in a more representative sample of evaluations of teacher training programs. The results of our meta‐analysis may not be externally valid, and it is possible that the results cannot be extrapolated to the rest of the LAC region. We also do not interpret the heterogeneity in the effect sizes because of the small number of studies. We were not able to conduct a stratified meta‐analysis by methodology or risk of bias because of the relatively small number of studies that focused on the impact of teacher training.

#### 7.5.2 Impact technology in education programs

Of the 24 included studies, four estimated the impact of a technology in education program on reading outcomes. We were able to include all of these studies in our meta‐analysis. The evaluations that focused on the impact of technology in education programs are summarized in Table 6.

**Table 6:** Primary studies that focus on the impact of technology in education

Studies	Definition of variable	Evaluation design	Included in meta‐analysis?	Country
Cristia et al. (2012)	Language test score	Cluster RCT	Yes	Peru
Ferrando et al. (2011)	Reading comprehension	Propensity score matching	Yes	Uruguay
Barrera‐Osorio and Linden (2009)	Language test score	Cluster RCT	Yes	Colombia
Beuermann et al. (2015)	Reading practices	Cluster RCT	Yes	Peru

Abbreviation: RCT, randomized controlled trial.

##### 7.5.2.1 Randomized controlled trials

**Figure 7:**
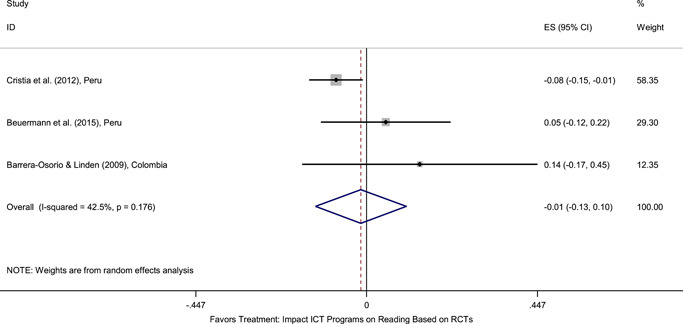
Impact of technology in education programs on reading outcomes on the basis of RCTs. CI, confidence interval; RCT, randomized controlled trial

**Figure 8:**
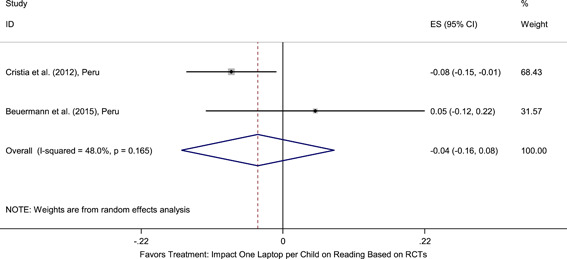
Impact of one laptop per child program on reading outcomes on the basis of RCTs. CI, confidence interval; RCT, randomized controlled trial

Figure 7 includes the results of the meta‐analysis for the RCTs of technology in education programs. We found no evidence to indicate that, on average, technology in education programs had a positive effect on reading outcomes (SMD = −0.01, 95% CI = −0.13, 0.10; evidence from three studies). The results of the one laptop per child program do not appear to be promising. In fact, the findings of Cristia et al. (2012) suggest that the nationwide one laptop per child program had negative effects on EGL outcomes in Peru and may have resulted in adverse effects on the reading habits of children. Beuermann et al. (2015) showed evidence for negative but nonsignificant point estimates in their estimates of the impact of the program on the number of hours that children allocated to reading books in a smaller sample in Lima, Peru. A separate meta‐analysis that focused on the impact of the one laptop per child program (see Figure 8) did not find evidence for statistically significant and negative effects of the program on reading outcomes if the sample was restricted to RCTs (SMD = −0.04, 95% CI = −0.16, 0.08; evidence from two studies). However, we found evidence for negative and statistically significant effects of the one laptop per child program on reading outcomes when we pooled the findings of quasiexperimental studies with the findings of RCTs in one meta‐analysis (SMD = −0.06, 95% CI = −0.11, 0.00; evidence from three studies). It nonetheless remains important to be cautious when interpreting these results because of the small number of studies.

Barrera‐Osorio and Linden (2009) found that a computer distribution program in Colombia had no statistically significant effect on the reading outcomes of third grade students. The authors also do not find any statistically significant effects of the program in their full sample of students (third through ninth grade). Barrera‐Osorio and Linden (2009) also found considerable evidence for challenges in implementing this program. In many cases, teachers did not use the computers in their instruction methods. This may explain why Barrera‐Osorio and Linden (2009) did not find any statistically significant effects of the program.

##### 7.5.2.2 Quasiexperimental studies

**Figure 9:**
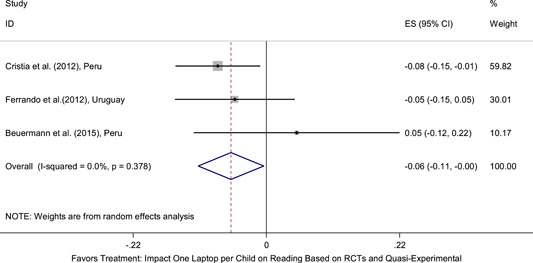
Impact of one laptop per child program on reading outcomes on the basis of RCTs and quasiexperimental studies. CI, confidence interval; RCT, randomized controlled trial

We found one quasiexperimental study that focused on the one laptop per child program in Uruguay. This study did not find evidence for statistically significant and positive or negative effects of this program on reading outcomes, but the point estimate is negative again. Furthermore, we found evidence for negative and statistically significant effects of the one laptop per child program on reading outcomes when we pooled the findings of this study in Uruguay with the findings of the RCTs in Peru in one meta‐analysis (SMD = −0.06, 95% CI = −0.11, 0.00; evidence from three studies). We report these results in Figure 9. It is important to be cautious when interpreting these results because of the medium risk of selection bias of the study in Uruguay. Nonetheless, the results are indicative of evidence that the one laptop per child program may have negative effects on reading outcomes in the LAC region.

Together, the findings regarding the impact of technology in education programs on reading outcomes in the LAC region suggest that technology in education programs do not consistently have positive effects on EGL outcomes and may indeed have negative effects in some cases.

#### 7.5.3 Impact of school feeding and other nutrition programs

Of the 25 included studies, five estimated the impact of a nutrition program on reading outcomes. We were able to include all of these studies in the meta‐analysis. These studies are summarized in Table 7.

**Table 7:** Primary studies that focus on the impact of nutrition programs

Studies	Definition of variable	Evaluation design	Included in meta‐analysis?	Country
Maluccio et al. (2009)	Reading comprehension	Cluster RCT	Yes	Guatemala
Adrogue & Orlicki (2013)	Language test score	Difference‐in‐difference analysis	Yes	Argentina
Ismail, Jarvis, and Borja‐Vega (2014)	Reading test scores	Propensity score matching	Yes	Guyana
	English test scores			
Powell et al. (1998)	Reading comprehension	RCT	Yes	Jamaica
	Spelling			
Simeon et al. (1995)	Arithmetic	RCT	Yes	Jamaica
	Spelling			
	Reading			

Abbreviation: RCT, randomized controlled trial.

##### 7.5.3.1 Randomized controlled trials

**Figure 10:**
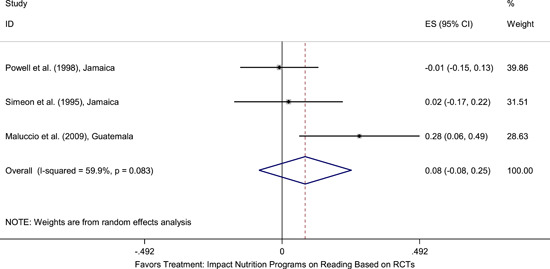
Impact of nutrition programs on reading outcomes in Latin America and the Caribbean region based on RCTs. CI, confidence interval; RCT, randomized controlled trial

We found no evidence that nutrition programs had positive and statistically significant average effects on reading outcomes in the LAC region on the basis of RCTs. Figure 10 shows the results from a meta‐analysis in which we included impact evaluations of deworming and a school breakfast program in Jamaica and an impact evaluation of a program that includes the distribution of supplementary nutritious drinks in Guatemala 25 years after the start of the intervention (SMD = 0.08, 95% CI = 0.08, 0.25; evidence from three studies). The studies in Jamaica do not show evidence for positive effects of deworming and a school breakfast program on EGL outcomes. However, we need to be careful in the interpretation of these results because both studies have a high risk of performance bias. The studies use student‐level RCT designs. As a result, the studies are likely to underestimate the impact of the program because of the risk of spillovers and contamination.

Maluccio et al. (2009) find evidence for positive effects of the distribution of nutritious supplements on reading outcomes in Guatemala. Although this study suffers from a medium risk of selection bias, the results look promising particularly because of the long timeframe of the study. However, the findings may be very context‐specific. Guatemala has the highest rate of malnutrition in the LAC region (Maluccio et al., 2009). Thus, nutrition programs may be particularly effective in this context. This example shows the importance of taking into consideration enabling factors in the analysis of reading outcomes. Programs with a focus on nutrition may be very effective in improving reading outcomes in specific contexts where malnutrition rates are high. We nonetheless need to exercise caution when interpreting this result, because the finding is based on a single study.

##### 7.5.3.2 Quasiexperimental studies

**Figure 11:**
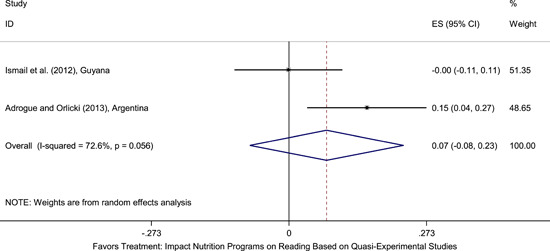
Impact of nutrition programs on reading outcomes in Latin America and the Caribbean region based on quasiexperimental studies. CI, confidence interval

We included two quasiexperimental studies of school feeding programs that estimated impacts on reading outcomes. These studies found no evidence that school feeding programs had positive and statistically significant effects on EGL outcomes in the LAC region (SMD = 0.07, 95% CI = −0.08, 0.23; evidence from two studies). For example, Ismail et al. (2014) found no evidence of positive effects of a school feeding program on EGL outcomes in Guyana. Adrogue and Orlicki (2013) present some evidence that a school feeding program in Argentina had positive effects on EGL. However, these results are not very convincing because they are based on an evaluation design with a high risk of selection bias. Thus, we do not interpret this finding as rigorous evidence of the positive effects of school feeding programs on EGL outcomes in the LAC region. Nevertheless, we present the results of the meta‐analysis in Figure 11.

##### 7.5.3.3 Pooled results

**Figure 12:**
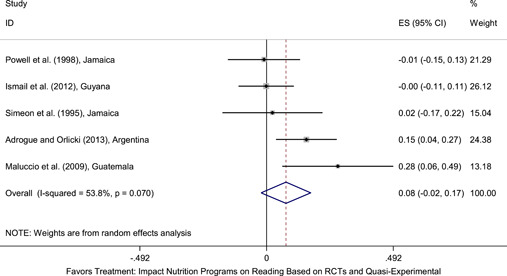
Impact of nutrition programs on reading outcomes in Latin America and the Caribbean region based on RCTs and quasiexperimental studies. CI, confidence interval; RCT, randomized controlled trial

We also present pooled results of the RCTs and quasiexperimental studies because the estimated effect sizes are similar and a metaregression does not show statistically significant differences in effect sizes. We again found no evidence of positive and statistically significant average effects of nutrition programs on EGL outcomes, but the results are close to statistically significant when we pool RCTs and quasiexperimental studies (SMD = 0.08, 95% CI = −0.02, 0.17; evidence from five studies). However, the positive results are driven by the study of Maluccio et al. (2009) in Guatemala and the study with a high risk of selection bias in Argentina. These findings indicate that nutrition programs may be effective in improving EGL outcomes, but only in contexts with high rates of malnutrition, such as Guatemala. The results also show substantial heterogeneity (*Q *= 8.65, *τ* ^2^ = 0.00, *I* ^2^ = 54%), indicating that the results depend on contextual characteristics. We present the results of the pooled meta‐analysis in Figure 12.

**Figure 13:**
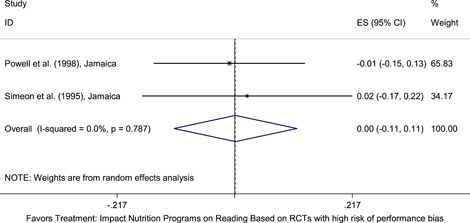
Impact of nutrition programs on reading outcomes in Latin America and the Caribbean region based on randomized controlled trials with a high risk of performance bias. CI, confidence interval; RCT, randomized controlled trial

In any case, we should be cautious when interpreting our results because the effects of several included studies with an emphasis on nutrition on reading outcomes may present underestimates of the impact of these programs because of performance bias. For example, two of the studies in Jamaica are likely to underestimate the impact of nutrition programs on reading outcomes for this reason. We present a separate meta‐analysis for these studies in Figure 13. The results show a difference between beneficiaries and no beneficiaries that is close to zero. This finding could well be explained by bias from spillovers or contamination. In that case, nutrition programs may be a promising approach to improve EGL outcomes but mostly in regions with high rates of malnutrition.

#### 7.5.4 Impact of school governance

Of the 25 included studies, two estimated the impact of a school governance program on reading outcomes. We used a narrative synthesis as opposed to a meta‐analysis for school governance programs because of the small number of rigorous studies that focus on this topic. The evaluations that focus on school governance programs are summarized in Table 8.

**Table 8:** Primary studies that focus on the impact of school governance programs

Studies	Definition of variable	Evaluation design	Country
Bando (2010)	Language test score	OLS regression analysis	Mexico
Lockheed et al. (2010)	Early literacy outcome	Propensity score matching	Jamaica

Abbreviation: OLS, ordinary least squares.

##### 7.5.4.1 Quasiexperimental studies

We included two quasiexperimental studies that focused on school governance and its impact on EGL outcomes. The first study focused on the impact of a cash transfer that is complemented by a matching grant as well as more responsibility for parents in decision making in primary schools in Mexico. Specifically, parents are given information and decision‐making power to spend the matching grant. This process can increase school accountability, which can, in turn, result in improvements in the quality of education and learning outcomes. The second evaluation focused on the impact of a school improvement plan that was accompanied by increases in school inputs for primary schools in Jamaica. These school inputs included teacher training elements, parent education, and school feeding programs, reading materials, and summer courses in math and reading. Essentially, the program resulted in changes in the implementation fidelity of other interventions. However, in contrast to the previously discussed evaluation studies, these activities are the results of changes in school governance as opposed to individual programs. Thus, we consider this study an evaluation of a school governance program and not part of any of the other program categories.

The two quasiexperimental studies did not find evidence that school governance programs had positive effects on EGL outcomes in the LAC region. The matching grant program did not show positive effects on EGL outcomes in Mexico (SMD = −0.05, 95% CI = −0.22, 0.13). The study in Jamaica also did not find evidence that the school improvement program had positive effects on reading outcomes in Grade 4 (we included this study because the students who were in Grade 4 during the endline survey were in early grades during the start of the program), but we were not able to estimate the effect size for the study in Jamaica. The lack of positive impacts could be explained by the small differences in the school inputs between treatment and comparison schools even after the positive effects on school inputs.

However, we should exercise caution when interpreting these results. Both studies suffer from a medium risk of selection bias and are not able to convincingly demonstrate that their identification strategies enable the estimation of causal effects of school governance programs. Hence, the included evaluations of school governance programs do not present convincing evidence on the impact of these programs on EGL outcomes.

#### 7.5.5 Impact of preschool programs

Of the 25 included studies, two estimated the impact of participation in preschool on reading outcomes. We focused on a narrative synthesis as opposed to a meta‐analysis for participation in preschool because of the small number of rigorous studies that focus on this topic. These evaluations are summarized in Table 9.

**Table 9:** Primary studies that focus on the impact of preschool

Studies	Definition of variable	Evaluation design	Country
Campos et al. (2011)	Language test score	Hierarchical regression analysis	Brazil
Felício et al. (2012)	Literacy score	Propensity score matching	Brazil

##### 7.5.5.1 Quasiexperimental studies

We included two quasiexperimental studies that focused on preschool and its impact on EGL outcomes in Brazil. Campos et al. (2011) argue that participation in preschool led to an improvement in language assessment scores for children in six Brazilian state capitals. They used hierarchical multivariate regression analysis to demonstrate the positive effects. We were not able to estimate the effect size for this study. Similarly, Felício et al. (2012) found that participating in early childhood education had positive effects on the literacy scores of children in second grade. They used propensity score matching to identify these positive impacts (SMD = 0.20, 95% CI = 0.06, 0.34).

Although Campos et al. (2011) and Felício et al. (2012) make valid attempts to identify the impact of participation in preschool on EGL outcomes in Brazil, the two studies both suffer from risk of selection bias. We rated the study of Felício et al. (2012) as having a medium risk of selection bias and the study of Campos et al. (2011) as having a high risk of selection bias. Thus, caution should be exercised when interpreting our results. Previous evidence suggests that participation in preschool can have a wide range of positive effects on children in low‐ and middle‐income countries (Martinez, Naudeau, & Pereira, 2012). However, the studies of Felício et al. (2012) and Campos et al. (2011) are likely to suffer from bias due to selection on unobservables. Hence, these studies do not present convincing evidence that participation in preschool leads to improvements in EGL outcomes. It is possible that participation in preschool has these effects in the LAC region, but more rigorous research is needed to demonstrate these effects. For example, preschool may only be effective when the education is of sufficient quality.

#### 7.5.6 Impact of teacher practices programs

Of the 25 included studies, six estimated the impact of the adoption of distinct teacher practices, such as the explicit instruction of new words, shared story book reading, and read‐alouds. We used a narrative synthesis as opposed to a meta‐analysis for teacher practices because the teacher practices that are discussed are very dissimilar. Therefore, we do not expect that a pooled effect size of these teacher practices would present any meaningful information. In addition, we were only able to estimate effect sizes for one study that includes two evaluations (Cardoso‐Martins et al., 2011). The evaluations that focus on teacher practices are summarized in Table 10.

**Table 10:** Primary studies that focus on the impact of teacher practices

Studies	Definition of variable	Evaluation design	Country
Larraín et al. (2012), experiment 1	Vocabulary acquisition	RCT	Chile
Larraín et al. (2012), experiment 2	Vocabulary acquisition	RCT	Chile
Cardoso‐Martins et al. (2011), experiment 1	Letter naming	RCT	Brazil
	Decoding		
Cardoso‐Martins et al. (2011), experiment 2	Letter naming	RCT	Brazil
	Decoding		
Neugebauer and Currie‐Rubin (2009)	Reading comprehension in Spanish and Quechua	RCT	Peru
Vivas (1996), experiment 1	Language comprehension	RCT	Venezuela
	Expressive language		

Abbreviation: RCT, randomized controlled trial.

##### 7.5.6.1 Randomized controlled trials

We included five RCTs that focused on the effects of specific teacher practices on EGL outcomes in the LAC region. These evaluations focused on distinct practices, such as the explicit instruction of new words, complex word elaboration during shared story book reading, and letter name teaching as opposed to only teaching the shapes of letters. The specifics of these tasks enabled researchers to examine how reading outcomes change in great detail. Researchers usually make use of this opportunity by estimating the impact of these practices on various reading constructs, such as letter recognition and vocabulary acquisition. Although the sample sizes for the included studies was small (*n* < 100 in the majority of the studies), researchers nonetheless found statistically significant effects in the majority of the studies. However, these statistically significant effects may suffer from publication bias. Evidence indicates that published studies with small sample sizes could be disproportionally affected by publication bias (Borenstein et al., 2009). In addition, we were only able to estimate effect sizes for two of the studies (Cardoso Martins et al., 2011; Neugebauer & Currie‐Rubin, 2009).

Although the results of the studies may be biased due to publication bias, the included studies on teacher practices present some findings about how specific teacher practices can influence reading outcomes. These findings can serve as hypotheses for larger‐scale research on which teacher practices are most effective in improving EGL outcomes. First, Larraín et al. (2012) presented evidence that word elaboration during shared story book reading has a positive effect on vocabulary acquisition. Larraín et al. (2012) also suggest that using simpler definitions of words is more effective in improving vocabulary acquisition than using complex definitions. In addition, Cardoso‐Martins et al. (2011) found that teaching the names of letters is more effective than merely teaching the shapes of letters (SMD = 0.94, 95% CI = 0.21, 1.68). Training in PA did not show statistically significant effects on letter sound recognition in the same study (SMD = 0.23, 95% CI = −0.65, 1.11), though this is possibly related to the small sample size, because the results were only no longer statistically significant after adjusting for possible small sample bias. Neugebauer and Currie‐Rubin (2009) present some experimental evidence that reading aloud can improve reading outcomes in Peru (SMD = 2.12, 95% CI = 1.11, 3.15), but the study has a high risk of selection‐bias considering that the sample size includes only two treatment and two control classrooms. Finally, Vivas (1996) demonstrates that listening to teachers reading stories aloud results in improvements in language comprehension and expressive language first grade children.

The results of the studies with an emphasis on specific teacher practices should merely be interpreted as interesting hypotheses for larger‐scale quantitative research for two reasons. First, as discussed above, there is some evidence for publication bias, which may invalidate the results of the studies because they are not replicable. Second, each of the included quantitative intervention studies with a focus on specific teacher practices suffers from a medium or high risk of selection bias. Each of these studies had a sample size that was too small to ensure equivalence in observable and unobservable characteristics between the treatment and the control groups. In addition, several of these studies made methodologically inappropriate choices in the design or analysis of the results. For example, Cardoso‐Martins et al. (2011) switched treatment students to the control group because the students self‐selected in the control group. These kinds of choices can result in a considerable risk of selection bias. Thus, we do not recommend that policy makers base their decisions on the findings of small‐scale quantitative intervention studies with a focus on specific teacher practices. However, it would be interesting to test the effectiveness of specific teacher practices on a larger scale.

#### 7.5.7 Impact of parental involvement programs

Of the 25 included studies, three estimated the impact of parental involvement with the aim of improving EGL outcomes, but we were not able to estimate effect sizes for any of the studies on parental involvement. We used a narrative synthesis as opposed to a meta‐analysis for parental involvement because of the small number of studies that focus on this topic. The evaluations that focus on parental involvement are summarized in Table 11.

**Table 11:** Primary studies that focus on the impact of parental involvement

Studies	Definition of variable	Evaluation design	Country
Tapia and Benítez (2013)	Reading practices	RCT	Mexico
Vivas (1996), experiment 2	Language comprehension	RCT	Venezuela
	Expressive language		
Murad and Topping (2000)	Reading practices	RCT	Brazil
	Reading comprehension		
	Reading Fluency		

Abbreviation: RCT, randomized controlled trial.

##### 7.5.7.1 Randomized controlled trials

We included three studies that focused on the effects of programs that involve parents on EGL outcomes. Both of these studies were RCTs with a small sample size and challenges in the implementation of the randomization. Tapia and Benítez (2013) found that teaching mothers about joint reading of stories and puppet play had the potential to improve their literacy practices with their children. In addition, Vivas (1996) presents evidence that listening to stories read aloud by parents results in improvements in language comprehension and expressive language in first‐grade children. Finally, Murad and Topping (2000) found positive effects of paired reading with parents on children's reading comprehension and fluency.

However, similar to the studies with an emphasis on specific teacher practices, it is possible that the studies with a focus on parental involvement suffer from publication bias. The studies show positive and statistically significant results despite being underpowered to demonstrate these effects. In addition, the studies have a high risk of selection‐bias. Thus, although the studies by Tapia and Benítez (2013), Vivas (1996), and Murad and Topping (2000) show interesting hypotheses that need to be tested in larger‐scale studies, we do not recommend that policy makers use these studies to inform their decisions.

### 7.6 Summary of effect sizes

We finalize the quantitative analysis with a focus on interventions with a table that shows a summary of the effect sizes of teacher training, technology in education programs, and nutrition programs on EGL outcomes. We highlight effect sizes based on meta‐analyses that pool RCTs and quasiexperimental studies for broad intervention categories.

The results demonstrate that teacher training, technology in education, and nutrition programs all do not show statistically significant effects on EGL outcomes, but the average impact estimates may hide significant heterogeneity. For example, the results suggest that teacher training may have positive impacts on EGL outcomes when it is combined with teacher coaching. In addition, nutrition programs may have positive effects on EGL outcomes in low‐income countries (specifically Guatemala) where rates of stunting and wasting are high. Finally, technology in education programs could have negative effects on EGL outcomes when they are not combined with a strong focus on pedagogical practices. We need to exercise caution in the interpretation of all these results, however, because of the small number of studies and the relatively high risk of bias of the included studies. Table 12 depicts the results.

**Table 12:** Summary of effect sizes based on meta‐analyses that pool randomized controlled trials and quasiexperimental studies

Programs	Mean effect size and CI
Teacher training	0.16 SMD (−0.17, 0.48)
Technology in education programs	−0.01 SMD (−0.13, 0.10)
Nutrition programs	0.08 SMD (−0.08, 0.25)

Abbreviation: CI, confidence interval; SMD, standardized mean difference.

### 7.7 Publication bias

It is possible that the included studies with a small sample size present a biased overview of the impact of specific teacher practices and parental involvement on EGL outcomes because of publication bias. The unusual high statistical significance in the studies with a smaller sample size shows the potential for publication bias in studies with a focus on teacher practices and parental involvement (Borenstein et al., 2009). However, we did not conduct a formal test of publication bias because of the small number of studies for which we could estimate effect sizes.

**Figure 14:**
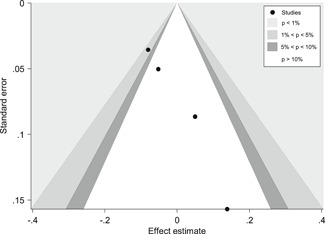
Funnel plot to test for publication bias in impact of ICT programs

**Figure 15:**
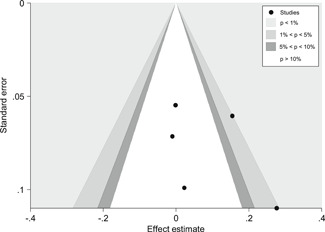
Funnel plot to test for publication bias in impact of nutrition programs

We also find some indications for publication bias in the studies focusing on the impact ICT that we were able to include in our meta‐analyses that include RCTs and quasiexperimental studies, but the evidence does not show indications for publication bias in the studies that focus on nutrition. We were not able to conduct a formal test for publication bias for the studies that focus on teacher training, because we were only able to include two studies in this meta‐analysis. We relied on funnel plots and the Egger test to examine the possibility of publication bias in studies focusing on nutrition and ICT programs. The idea underlying funnel plots is that publication bias is most likely when effect sizes do not follow a normal distribution. We present the funnel plot in Figures 14 and 15. We formally tested for publication bias by applying the Egger test. This test did not indicate evidence for publication bias in the studies that we included in the meta‐analysis for nutrition programs (*β *= −.08, *SE *= 0.20; *p *= .51), but the Egger test did show some evidence for publication bias in the meta‐analyses focusing on ICT programs (*β *= −.15, *SE *= 0.02; *p *= .02). We have to remain careful in interpreting this result, however, because tests for publication bias are only indicative of publication bias. There may be other explanations for the nonnormal distribution. Nonetheless, our results suggest that publication bias may be present in our larger‐scale ICT studies as well.

### 7.8 Qualitative synthesis

In this section, we present the results of studies that were rated as high and medium quality by EGL topic area. Studies that did not clearly identify the research questions or justify the study design were deemed to be low quality and were removed during the final quality review. In addition, studies that did not provide adequate details about data collection and analysis so that the reviewer could understand the decisions that were made were also deemed low quality. Several studies did not make their findings explicit and several did not present sufficient data to justify their findings. In addition, all of the studies deemed low quality did not address the relationship between researcher and participant nor did they address any ethical issues related to the study. Refer to the systematic review phases flowchart in Figure 4. The studies shown in the “Final” phase are those that were deemed to be “medium” or “high” quality and which included 23 quantitative intervention, 61 quantitative nonintervention, six qualitative intervention, and 14 qualitative nonintervention studies. These are the studies that are included in the below analysis highlighting the main findings across the articles within our topic areas.

#### 7.8.1 Assessment

One qualitative nonintervention article focused on literacy assessments from multiple countries (Leal Carretero & Suro Sánchez, 2012). Researchers analyzed 21 different tests with measures of PA that were gathered through a detailed literature search with specific inclusion criteria. Tests had to target Spanish‐speaking preschool children and include specific questions focusing on phonemic awareness. The researchers found 26 unique tasks among the 21 tests that measured PA. Among the 26 tasks, nine were productive tasks such as repeating syllables or constructing words from a sequence of word segments. Nine tasks involved implicit categorization such as identifying the number of syllables in a word or the number of words in a sentence. The remaining eight tasks involved explicit categorization such as categorizing the words with the same syllable or categorizing words with the same ending. The fact that there was so little coherence among the 26 tests and such a wide variety of tasks indicates that there is little consensus as to which tasks most accurately measure PA. In addition, many of these tasks were very prone to errors and often did not even measure PA because of the way that the tasks were worded. Tests did not measure syllable structure, or subsegmental, melodic, metrical, or intonation awareness, all of which could be useful measures of PA. Findings from this review of literacy assessments on PA indicate that:

- PA tests should systematically include tasks that measure students' awareness of syllable structure (i.e., each syllable has a hierarchical organization formed around a core vowel).
- Current testing may be enriched by adding tasks for metrical awareness.
- Tests could be enriched by adding tasks for either intonation awareness or melodic awareness.
- Tests might be enriched by adding tasks for subsegmental awareness since a segment is not indivisible but instead has distinctive sound features.
- Synthesis of correlational studies.

#### 7.8.2 Curriculum

The team found only one qualitative intervention article on curriculum (Roofe, 2014). It focused on the implementation of Jamaica's revised primary curriculum in 2014. Although the article is specific to the Jamaican context and has some gaps in information about the data collection methods, the authors recommended some principles that could be applied to a wide range of contexts. For example, the authors pointed to a need for alignment between pedagogical and assessment practices for new curriculum; a rigorous implementation plan for training teachers and principals who will use the curriculum; a monitoring and evaluation system to hold individuals accountable; and finally, training materials that provide sample lesson plans and examples of how users can adapt curriculum to suit their contextual needs. However, although the curriculum aims to emphasize literacy development as a “key indicator of improved quality education,” the authors determined that parts of the curriculum “disadvantaged students with low ability levels” in literacy development, as well as students from rural areas on topics for writing activities (p. 4). This finding is consistent with the theme we identified elsewhere in qualitative and quantitative studies: that poverty is a strong contextual factor in explaining student learning.

#### 7.8.3 General pedagogical approaches

The team included two qualitative intervention articles and four qualitative nonintervention articles that discussed general pedagogical approaches (i.e., approaches which were not specific reading approaches). Most of the approaches across articles centered on context and environment—that is, how students interact and are involved in the construction of their own learning. For the most part, the articles presented strong methodologies that link their conclusions to the data. Therefore, much of the information in the pedagogical articles could be reliably adapted to fit other contexts based on need.

##### 7.8.3.1 Qualitative intervention

The qualitative intervention article on collaborative learning approaches in Colombia received high ratings on most quality criteria. Gonzalez et al. (2013) examined how the use of collaborative work in the classroom can aid in the development of students' writing skills.

The study observed students using three collaborative learning strategies that could be adapted to other contexts. The first activity entailed students outlining the task, preparing individually assigned parts, and then coming together to revise the whole document with other students. Teachers observed that students allowed group‐level decisions to prevail over their own interests. In the second activity, students played specific roles in the writing process based on their abilities (i.e., writer, idea proposer, leader, compiler, editor). The authors noted that students comprehended “the relevance and importance of their contributions to the initial task,” which enabled students to rely on their peers to support their roles (p. 23). In the third strategy, students worked together on the entire development of the document, which allowed the interactions to be more natural and also allowed students to freely use language to communicate ideas.

The authors conclude that collaborative learning approaches are “an opportunity for students to help each other to construct meaning and knowledge, as they work on tasks that demand analyzing, planning, acting, and reflecting on their work as a tool to measure their capacity to work with others” (p. 24). Specific teacher training materials or more specific information on how to implement these strategies and encourage collaborative work in the development of reading and writing skills would be a useful supplement. Teacher trainers should consider looking into how collaborative work could enhance reading and writing abilities in their contexts as students can support each other in the learning process. Researchers could implement quantitatively oriented studies to understand how this strategy might be effective in other contexts (such as poorer schools).

The second article (Mahurt, 1993) was a case study of a single teacher focusing on the decision‐making process that leads a teacher to change literacy instructional practices. The study examined how this teacher decided to enact changes in their practice from skills‐based to whole language teaching and what that decision‐making process looked like as well as how it played out in the classroom setting. It is difficult to draw conclusions from the study of one teacher in one specific context but this study does highlight the time that behavior change can take which is an important consideration in teacher training interventions.

##### 7.8.3.2 Qualitative nonintervention

One qualitative nonintervention article that discussed pedagogical approaches encouraged reflection and questioning among children about their educational experiences in Brazil (Rosado & Campelo, 2011). The authors argued that considering children's input on the learning process is essential because it contextualizes their reading experience. Understanding children's perspective on learning allows educators to control for those factors that can impact children's perspectives of themselves as learners, impact learning performance, and impact the motivation to learn. Awareness of the importance of young children's views is an issue that can be included in the teacher training process. NGOs can work in this area as well by developing projects that support the social‐emotional aspect of learning, particularly motivation.

A second article (Gómez Nashiki, 2008) also argued for incorporating into the classroom aspects of student experience that students consider important. The article focused on strategies to increase the reading level among Mexican students in study areas by conducting a survey of youth about their reading preferences. The author lays out specific recommendations that came out of the survey—as well as a series of proposals from teachers—including: having students create a personal dictionary, having students make their own book, and to establish a reading club. This methodology is similar to others that advocate involving children in the design of classroom activities to contextualize their experiences. This research could be particularly useful for teacher strategies or as a model for teachers in other contexts to conduct their own student surveys and choose teaching practices based on the results.

The third article (Medina & Costa, 2013), about a study in Puerto Rico, discussed context through looking at “children's curricular engagement with the Spanish television genre of telenovelas in relation to classroom critical literacy and performative inquiry.” Keeping with the theme of involving children in learning, this study was student led and negotiated. The authors argued that such a lens is important because processes are increasingly becoming globalized, and therefore it is critical to understand how these global processes are being embodied at the local level. Through methodologies such as observation and artifact collection, the authors found that “the idea of reading, writing, and producing across communities could also serve as a powerful lens for engaging in the creation of expansive classroom critical literacy pedagogies” (p. 187). However, the analytical frameworks the authors used in their write‐up do not necessarily lend themselves to practical application, especially in contexts where the telenovela is not necessarily prevalent. Nonetheless, the context of globalization and “new ways of reading, interpreting, and producing as children navigate across local global spaces” speaks to the importance of context discussed in the other articles.

The fourth article (Ribeiro & Souza, 2012) discussed the importance of considering context in learning to read and other literacy practices in Brazil. This article was similar to the intervention articles that discussed importance of context in learning pedagogy. The study aimed to understand the impact of certain types of written material on children and found that children recognized maps, medicine labels, newspapers, storybooks, traffic signs, and comic strips with the greatest frequency, indicating that this type of written material speaks to experiences in their lives. However, the authors did not address the practical use of such strategies in the classroom.

This research provides insight into the genres of literature that children commonly recognize. The researchers recommend “considering the processes of literacy in both the pedagogical strategies of early childhood education, and in speech therapy with students who have difficulties and/or disturbances in the acquisition of writing” (translated from Portuguese). This research also provides insight into the forms of the written word that children commonly recognize and how context impacts learning. In addition, the research argues that reading materials and pedagogies should include lived experiences, as children already come to school with a rich knowledge base that can be used to motivate interest in learning to read. This data could contribute to the development of reading materials that target the contexts in which children focus on the written word in their daily lives and to expand on such genres for pedagogy.

##### 7.8.3.3 Summary of general pedagogical approaches

These articles focus primarily on the use of collaborative work, engaging children in decisions about what and how they read and ensuring the contextual relevance of reading materials. More research is needed to draw conclusive findings about the influence of these factors on reading improvement, but these studies suggest that involving students in their own learning and giving them a voice in what and how they learn may have positive outcomes.

#### 7.8.4 Parental and community participation

The team included one qualitative intervention article and three qualitative nonintervention articles on parental and community participation that met the basic inclusion criteria. The articles argue that home and community contexts should be considered in children's literacy experiences.

##### 7.8.4.1 Qualitative intervention

Stein and Rosemberg (2012) discuss how living with extended families in Argentina may contribute to children's literacy development. Particularly, the authors argue that, “it is important to interweave early educational interventions with the funds of knowledge and interactional patterns that characterize children's culture.” In this case, the culture meant that “the literacy situations took place within the framework of the interaction between the child and the diverse and multiple participants that comprise the collaboration networks where children and adults assume different roles.” This theme of considering the importance of a child's context in his or her literacy experience was evidenced throughout articles across all categories in the review.

##### 7.8.4.2 Qualitative nonintervention

The three qualitative nonintervention studies also center on the idea that context and social experience drive a student's literacy experience. Kinkhead‐Clark (2014) studied immigrant kindergarten children in Jamaica using interviews, artifacts, and school and family observations and found that, “literacy serves a unique purpose to the family unit. Their experiences with literacies reflect their cultural identities and the value they place on its role as an agent of change.” Although this study heavily advocates for considering context when forming a student's classroom experience, the authors do not present specific strategies that could potentially be extrapolated.

Volk and de Acosta (2001, 2003) conducted three ethnographic case studies of children in mainland Puerto Rico to understand “syncretism,” or how students draw from the various contexts in which they interact to construct literacy events. The studies addressed communication within particular social cultural contexts, which is important for sensitizing education stakeholders on how dominant instructional narratives practices can drown out the phenomenology of children's experiences in the learning process—that is, the experiences that they bring to school and the content, form, and meaning of their communications.

Their findings indicate that the three children in Puerto Rico were able to reconstruct literacy lessons using stories, texts, and other tools from their own contexts—a finding supported by other literature. The study does not describe some essential elements of the research, such as the justification for the methodology or a discussion of the evidence against the researchers' interpretations. In addition, the case study methodology does not allow for extrapolation of findings to other contexts (which the authors address).

The authors indicate that the study contains lessons for sensitizing preservice teachers to different cultures about which they are unfamiliar in teacher education, including through observations of “students literacy learning in homes and communities” (p. 40) and a discussion on how school literacies are often privileged while others are dismissed—including in teachers' own biases. Finally, the authors also recommend that teachers learn how to “co‐construct syncretic literacy with children” (p. 40) and how to add to school‐centered approaches by consulting families to help construct specific goals for their children appropriate to their skill levels and context.

##### 7.8.4.3 Summary of parental and community participation

The studies on parental and community participation highlight two key themes, the importance of context and the home environment. These studies all point out that children are a product of their environments and that they come to school not as blank slates but having already learned a great deal from interactions in their home and community. These experiences then drive their later literacy experiences and frame how they view reading and writing. More research is needed to verify these findings and to shed light on the specific mechanisms by which particular home and community experiences prior to schooling can set a child up for later reading success.

#### 7.8.5 Reading in bilingual/multilingual contexts

Two qualitative articles focused on learning to read in bilingual/multilingual contexts. One qualitative intervention article (Neugebauer & Currie‐Rubin, 2009) focused on using the read‐aloud technique to develop Spanish vocabulary and comprehension skills in native Quechua speakers in Peru. One qualitative nonintervention article from Colombia (Guevara and Ordoñez, 2012) discussed reading in bilingual/multilingual contexts, with a focus on learning English in a dominantly Spanish speaking context.

##### 7.8.5.1 Study 1

Neugebauer and Currie‐Rubin (2009) conducted a mixed‐methods study with first‐grade indigenous Quechua speakers in Calca, Peru. There were two control and two intervention classes with a total of 26 and 29 students, respectively. While control classes continued business as usual, researchers trained intervention teachers in seven specific read‐aloud techniques. Both groups of teachers were given a set of three books on which they were asked to focus their teaching during the normal 30‐min class period five times a week for 3 weeks. Students in the experimental group scored 30 more correct items on the vocabulary assessment than their peers in the control group after only 1 month of the intervention. These data seem to support the effectiveness of read‐alouds and the specific read‐aloud techniques for promoting vocabulary acquisition in second language learners.

Much of the research on the importance of read‐alouds thus far has focused on learners of English as a second language in the United States. This research emphasizes providing definitions and contextual information about vocabulary and “actively involving students in word learning through talking about, comparing, analyzing and using the target words” (August, Carlo, Dressler, & Snow, 2005, p. 54). The study of Neugebauer and Currie‐Rubin (2009) appears to be one of the few of its kind focusing on the topic of read‐alouds for second language learners in the LAC context. In addition, the researchers argue that read‐alouds are particularly effective as a pedagogical strategy for indigenous learners who come from a culture where oral traditions are strong as read‐alouds combine oral discussion with written narratives. However, this study suffers from a high risk of selection‐bias because of the small sample size. Thus, we should be cautious in interpreting these results.

##### 7.8.5.2 Study 2

Guevara and Ordoñez (2012) conducted a qualitative study designed to evaluate a newly developed kindergarten curriculum focused on incorporating authentic communication experiences in order to improve language learning in a bilingual education program in Colombia. The new curriculum focused on building connections between students' first language (Spanish) and English, finding authentic ways for students to practice oral English, as well as promoting interaction and cooperation between students. In order to determine the perception of teachers about the relationship between the curriculum and children's attitudes toward English and learning of English, researchers analyzed four teacher interviews and four classroom observations over the period of a year in addition to two classroom recordings done by the teachers. Researchers found that children:

- Developed positive attitudes toward the foreign language class
- Showed increased motivation and interest to use the foreign language (English)
- Participated more in class

Teachers reported that students showed great improvements in oral vocabulary because of the focus on expressive vocabulary through authentic performances as opposed to the previous focus on written language and receptive skills. Study data showed that students produced a lot of language orally and learned to communicate in different daily situations using accurate structures and vocabulary.

The study presents an interesting case for incorporating authentic ways for children to practice foreign language skills (particularly in contexts where there is not a lot of exposure to the language outside of the academic context). Teachers most commonly incorporated games, role plays, songs, and stories and engaged the children in selecting topics and ideas that would be most relevant to them which, in turn, led to improved student attitudes and increased motivation and participation. The focus on oral English enabled students to “advance in their Spanish literacy process before a different reading system was introduced” (Guevara & Ordoñez, 2012, pp. 16–17). This finding is supported by current research on learning multiple literacies in multilingual contexts indicating that making use of students' knowledge of their first language is key to developing literacy in a second or additional language (Cummins, 1979; Koda, 2008; Verhoeven, 1994). However, we need to be careful when interpreting this finding because we only found one study that supports this finding in our review.

##### 7.8.5.3 Summary of reading in bilingual/multilingual contexts

Both of the high‐quality articles on reading in multilingual contexts share some common themes. Both articles: (a) recognize the importance of using and building on the first language in the development of literacy in the second language; (b) focus on building oral vocabulary in the second language to support reading comprehension; and (c) focus on connecting language learning to real life “authentic” experiences and building on what students know and the context they are familiar with in their daily life.

#### 7.8.6 Reading skills

Only two of the 20 qualitative articles specifically focused on reading skills. Both were nonintervention research articles. One focused on comprehension and the second focused on deaf children's construction of writing.

One study aimed to identify the comprehension difficulties faced by 4‐ and 5‐year‐old children from low‐income populations during story reading at kindergarten, in Buenos Aires, Argentina (Manrique & Borzone, 2010). Researchers analyzed the teacher–student interactions during 26 story‐reading settings in nine different kindergarten classrooms and identified three main types of difficulties children faced when trying to comprehend a text that was read to them: (a) illustration‐level difficulties, (b) text‐level difficulties, and (c) teacher–student interactions. Illustration‐level difficulties often occurred when there was a disconnect between the pictures and the text being read, when pictures did not accurately represent the text, or when the pictures contained too much detail and therefore became distracting from the story. Text‐level difficulties arose when a text included complex or abstract vocabulary that had not been adequately explained to the children, metaphors, or narrative structure. Teacher–student interactions that led to comprehension difficulties occurred when teachers focused only on explicit aspects of the text such as asking students “what colour was the umbrella” or “what was the boy's name,” which caused students to focus on those very specific details as opposed to helping them get a better overall picture of what was happening in the story. In addition, researchers found that when teachers did not express the emotions elicited by a story, children experienced a disconnect with the text.

The findings from this research indicate that for very young learners, there are specific text and picture factors as well as teacher interaction factors that can affect their comprehension of stories being read aloud to them. Specifically, the findings show the importance of (a) coherence between the illustrations and text of a story and a need for illustrations that are simple and clearly representative of the text, (b) vocabulary that is understood by the students (or which is clearly explained in the context of the story) and the avoidance of metaphors and narrative structures, and (c) the ability of students to focus on the meaning of the story through more implicit questioning as well as embodying the emotion of the text. However, more research is needed on this theme because we only found one study that focuses on specific text and picture factors and the relationship between teachers and students.

The second study by Massone and Baez (2009) set out to explore the way in which deaf children acquire written language by categorizing deaf children's ways of interpreting illustrated texts and determining the compatibility of the various processes through which hearing and deaf children learn written Spanish. The sample for this study included 15 deaf children from kindergarten through second grade attending special schools in the cities of Rosario and San Nicolás in Argentina. The children in the study signed several Argentinian Sign Language varieties and had been poorly trained in oral Spanish and not systematically taught to read and write. The researchers carried out individual interviews with participants using nine cards, each containing an image and a string of written words. Participants were then asked to make hypotheses about the connection between the image and the text, and to identify what meaning they ascribe to the text and image.

Initial findings from this study show that deaf children initially go through the same developmental progression as hearing children whereby they at first they are unable to distinguish between text and pictures. Toward the end of the progression, however, hearing children see the “graphic marks” or symbols and see these text segments as equivalent to spoken Spanish. Deaf children, on the other hand, translate the components of the written text into sign language. This study has implications for the teaching of literacy to deaf children and the ways in which that might differ from teaching literacy to hearing children based on their different language paradigms. However, more research is needed as a sample of 15 students in one country is not enough to generalize these findings.

#### 7.8.7 Teaching practices for reading

There were three qualitative nonintervention articles that reported on teaching practices for reading.

##### 7.8.7.1 Study 1 (Webster, 2009)

In this study, the researcher worked with a single teacher and her class of 30 Grade 1 students in a rural primary school in Jamaica to determine the relationship between teacher read‐alouds of informational texts and students' science learning (as revealed through vocabulary).

The study found that first graders used their own realities to make connections with informational text—that is, they draw on their background knowledge and experience to enhance their understanding of the text. A second finding is that directed look‐backs—where the students and teacher go back through the pages of the story to find information—can enable students to gather important facts about the topic of the book and to internalize this technique as a useful literacy strategy. Finally, teacher read‐alouds are associated with student content knowledge and expand student vocabulary about the story topic. The results of this research suggest that before, during, and postreading activities led by the teacher may contribute to the success of read‐alouds in developing students' vocabulary and comprehension skills. However, the study design does not allow for making causal claims about the impact of read‐alouds.

##### 7.8.7.2 Study 2 (Jiménez et al., 2003)

This study examined the language and literacy practices in two Mexican schools over a period of approximately 6 months in two preschool and two Grade 4 classrooms. Researchers conducted 34 classroom observations, interviews with teachers and school principals, and document analysis. In addition to identifying the literacy practices used by students and teachers, researchers sought to determine the ways in which spoken language, reading, and writing were viewed and regulated.

Researchers found that students were given considerable freedom in terms of their spoken language as evidenced by the high noise level in the classrooms and students interjecting while the teacher was talking and asking questions and talking openly with their classmates without any censure from the teacher. This freedom of oral expression contrasts with the emphasis on correct form in students' written work as evidenced by the focus on proper spelling, good handwriting, and general neatness. Reading seemed to fall in the middle depending on whether students were reading silently or aloud. When students read aloud, they were subjected to much more control by teachers as to their pronunciation and inflection and it was clear that their oral reading was expected to be fluent and flawless. However, when students were allowed time to read as they pleased, this was completely unregulated by teachers, and students could be seen reading silently, reading in groups, and informally discussing the text and illustrations.

It is difficult to extrapolate the findings of this study as the purpose was primarily to identify existing literacy practices in a specific location. Studying the regulation of different literacy practices by teachers could be a necessary first step in implementing changes to teaching practices in order to determine how literacy is currently taught as well as whether the emphasis is on different aspects of the literacy process.

##### 7.8.7.3 Study 3 (Diuk, 2007)

The aim of this study was to analyze the reading and spelling acquisition process of two first grade girls in Buenos Aires, Argentina. Reading tests were given to both girls at the beginning of the year focusing on skills such as the recognition of rhymes, initial sounds of words, letter knowledge, and the reading and writing of words. Researchers administered another reading test at the end of the 1st year (35 weeks of class) to see what changes had occurred in the girls' literacy skills. The girls were asked to self‐report on strategies they used during the reading and writing of words. As in previous studies, this study found that the girls both relied on logographic strategies in the initial stages of literacy learning but slowly developed more analytical strategies. The authors suggested that poor reading levels of children in marginalized contexts may be the consequence of not providing them with adequate instructions on metaphonological strategies and explicit and systematic phonics. However, with a sample size of only two children, this study cannot credibly make these claims but only suggest this as a possible avenue for future research.

##### 7.8.7.4 Summary of teaching practices for reading

These three studies although focused on teaching practices for reading focused on very different aspects of reading and, therefore, cannot be summarized as a whole. In addition, each of these studies included very small samples and thus results are not generalizable to the larger population.

#### 7.8.8 Teacher training

We included one qualitative intervention article that related to teacher training (Warrican et al., 2008) which discussed challenges exemplary teachers in the Caribbean faced in promoting literacy among students using a model shown to be effective in promoting literacy in students. Although the article does not provide an in‐depth description of the program elements, the authors state that teachers receive training in a wide variety of teaching methods that contribute to their understanding of literacy development (e.g., PA, word recognition, and fluency) as well as differentiated instruction, student‐centered activities, and the use of action research.

The mentoring, training, and the collaboration that is fostered through working together on problems and finding solutions, result in a validation of the teachers that leaves them feeling cared for and special. Despite the often difficult circumstances under which they find themselves, these teachers are thus unlikely to experience the isolation that others in equally challenging situations experiences (p. 28).

More generally, the training may have allowed the teachers “to acquire knowledge and skills that brought about noticeable changes in some classrooms;” however, more explicit linkages from specific project elements to specific outcomes would help to determine which elements are a priority and why. As with the articles on parental and community participation and reading materials, the teacher training article advocates encouraging teachers to create a highly contextual literacy environment for students.

#### 7.8.9 Synthesis of quantitative nonintervention studies

Multiple themes emerged from the corpus of quantitative nonintervention studies. These included preschool programs; preliteracy/emergent literacy; individual differences in reading skills, poverty, disability, and assessment validation. Although some themes were interrelated, others were multidimensional, cutting across different themes. For example, one study measured PA but also examined quality of the preschool program (Pino & Bravo, 2005). Another study investigated the factors that were associated with student reading ability and found that school‐level factors (e.g., teacher quality and student abilities) predicted 40% of students' academic performance, while the authors reported that home factors (e.g., poverty) account for more variance in school performance (Ramírez et al. 2000). Refer to Table D4 in Appendix D for quality ratings for all quantitative nonintervention studies.

##### 7.8.9.1 Preschool

In the sample, 17 of the 61 studies focused on the overarching theme of preschool programs including the importance of preschool (seven studies) and the quality of preschool programs (10 studies) (Pino and Bravo, 2005). Studies featuring the importance of preschool ranged from those finding a correlation between literacy and other measures of cognitive development (comparing cognitive) and more years of preschool related to better academic outcomes (Benítez, Vargas, Hernández, Sánchez, & García, 2007; Castro et al., 2002; Oliveira, 1996). The studies with an emphasis on the quality of preschool included studies related to programming and pedagogical practices (Bravo, Villalón, & Orellana, 2002; Pino & Bravo, 2005) to type of school as measured by rigor of preschool program (Gómez‐Pérez, Sierra, Jiménez, & Méndez, 2011). Studies described characteristics of preschools in low socioeconomic areas (Silva et al., 2013), including teacher quality and materials used (Oyarce & Mujica, 2001), and teacher quality and parent education levels (Fuller et al., 1999).

##### 7.8.9.2 Preliteracy/emergent literacy

Several studies focused on preliteracy skills and the importance of early exposure to print (Guardia, 2003; Kessler, Pollo, Treiman, & Cardoso‐Martins, 2013) and oral language development (Páez et al., 2007) to reading acquisition. This finding is supported by other studies that linked oral language to reading and writing ability (Correa & Dockrell, 2007) and to the writing ability as a product of the sociocultural background of the student (Ribeiro & Souza, 2012). These findings suggest that students' reading and writing abilities are directly related to the level of oral language they have at school entry and the linguistic influences they have had before entering school. From these studies we find that the quality of the preschool program, the quality of the teachers, and the materials used are all associated with student achievement.

##### 7.8.9.3 Reading skills

Of the 61 studies, 22 studies involved a measure of one or more reading skills (e.g., PA, phonics, decoding, comprehension, vocabulary). Of these, 10 studies focused on some element of phonics and the alphabetic principle, including letter‐sound correspondence rules, letter recognition, and word level reading. Study findings support the idea that students with better letter recognition skills can read better (De Abreu & Cardoso‐Martins, 1998; Guardia, 2003; Medeiros et al., 2011). Taken together, these studies found that explicit teaching of letter‐sound correspondence is associated with children's decoding skills (i.e., the connection between sounds and symbols). An additional nine of the 22 reading skills studies found a strong correlation between PA and reading ability (Bravo et al., 2002; Plana & Fumagalli, 2013). Several studies found that teaching PA and phonics is associated with student decoding skills (de Manrique & Signorini, 1994; Reynoso‐Alcántara et al., 2010). One study from Chile found that rapid letter naming and PA were the strongest predictors of reading ability even for children from low socioeconomic homes who had less exposure to print at home (Guardia, 2003).

Another study from Chile found that, although some students with strong PA skills become strong readers, some do not because other factors interact with reading such as the instructional methodology and student motivation (Muñoz, 2002). A third study from Chile found that PA, phonics, reading, and writing are all significantly correlated, supporting the belief that these skills may be interrelated (Villalon & San Francisco, 2001). The last four studies of reading skills centered on decoding and comprehension. Three of these studies investigated finding a relationship between fluency and comprehension (Abadzi et al., 2005; Kudo & Bazan, 2009) while one found a relationship between numerical fluency and reading fluency (Reigosa‐Crespo et al., 2013). All these studies are correlational and cannot be interpreted as causal evidence.

Although the studies consistently provided evidence for significant associations between phonemic awareness and early word reading skills, one study suggested that phonemic awareness‐focused instruction may not be as useful for Spanish‐instructed children as a teaching approach, as compared with English‐instructed children (Goldenberg et al., 2014). When tested on phonemic awareness, Mexican students performed worse than students in the United States, although both groups were instructed in Spanish. The researcher suggests that this is a product of strong phonemic awareness instruction in the United States, after controlling for various other factors including parental education. Interestingly, children in the United States performed better on Spanish phonemic awareness, even though they were only provided phonemic awareness training in English, providing strong support for cross‐linguistic transfer. Despite this advantage in phonemic awareness, however, the Mexican children outperformed the other students in later and repeated measures of reading, suggesting that phonemic awareness may not be as necessary for sustained teaching when learning a transparent orthography such as Spanish (Goldenberg et al., 2014).

Multiple researchers stated that there is a zone of proximal development for students to benefit from PA and early exposure to print to learn to read efficiently (Bravo et al., 2002; Guardia, 2003).

The findings indicate that teaching phonemic awareness, phonics, fluency, and comprehension is associated with reading ability, but it is unclear whether this relationship is causal, and for how long such teaching is likely to impact reading outcomes. Thus, there may be a positive effect of teaching these abilities on reading comprehension, but there are several confounding factors that could bias the relationship. Neither the quantitative intervention nor the quantitative nonintervention studies are able to provide conclusive evidence on the effects of teaching phonemic awareness, phonics, fluency, and comprehension on reading ability. This is an important gap in the literature on EGL in the LAC region.

##### 7.8.9.4 Poverty

Of the 61 studies, six present an association between poverty and associated factors and the ability to read. One study (Guardia, 2003) from Chile found that young children have a natural disposition for development of psycholinguistic and cognitive abilities that support reading acquisition, but these children need a print‐rich environment to benefit from being read to by parents. The authors suggest that there is a “zone of proximal” development for reading acquisition enhanced by explicit and systematic instruction in PA and, in particular, rapid letter naming that supports early reading ability. Children from impoverished homes are less likely to have either of these present in their homes. Similarly, another study from Chile (Bizama, Gutiérrez, & Sáez, 2011) found that poverty is adversely related to children's academic performance in reading, highlighting the educational inequalities that poverty creates. Two studies investigated the effect of child labor on reading achievement. Students who work more hours have the lowest student achievement (Cervini, 2015) and those who get paid to work tend to have worse academic outcomes than those paid in kind (Torrecilla & Carrasco, 2014). One study from Guatemala focused on the predictive effects of child nutrition on growth and cognitive achievement as well as later adult outcomes (e.g., wages for men, family formation, reproduction, and poverty; Hoddinott et al., 2013). Taken together, these studies demonstrate the apparently long‐lasting associations of poverty and school achievement and later life choices, especially through the relationships they have with access to educational resources.

In all, these studies indicate that poverty and reading ability are negatively correlated, which is supported by some of the quantitative intervention research. Both the quantitative intervention research and the quantitative nonintervention research suggest that poverty and associated factors, such as nutrition and child labor, are negatively associated with EGL outcomes. However, the evidence is less clear on the direction of these effects. Although poverty and reading ability are negatively correlated, the quantitative intervention studies only find evidence for a positive effect of nutrition programs in countries where the incidence of stunting and wasting is very high. In other contexts it remains unclear whether confounding factors bias the relationship between poverty and EGL outcomes.

##### 7.8.9.5 Disability

Three of the 61 studies in the sample investigated reading ability in students with disabilities. One study from Brazil investigated reading ability in children with hyperlexia and found that these students showed a discrepancy between word decoding and reading comprehension and that these traits are also found in preschool‐aged students (Cardoso‐Martins, & Da Silva, 2010). Another study from Brazil compared the differences between how deaf children interpret illustrated text and construct writing to that of hearing children and found differences in the two groups. Bandini et al. (2006) studied how children who are deaf learn to read and found that the students who signed followed the alphabetic principle and used a pattern similar to nondeaf children.

##### 7.8.9.6 Assessment validation

Of the 61 studies, nine studies involved a form of assessment validation. For example, two studies (Athayde, Giacomoni, Zanon, & Stein, 2014; Dias et al. 2006) assessed the Teste do Desempenho Escolar (TDE) instrument that is widely used in Brazil. Study findings differed, with one study finding that discrimination power of the writing subtest could not distinguish between students of similar grades (e.g., 3/4 and 5/6; Athayde et al., 2014) and another finding only differences between fifth‐ and sixth‐grade results (Dias et al. 2006). Similarly, Athayde et al. (2014) found that the TDE test could only discriminate between scores of students in Grades 1–3 but not 4–6. These results indicate that the TDE test may be best when administered on the early grades (e.g., 1–3). Another study measured the predictive validity of the ABC test (Salazar, Amon, & Ortiz de Urdiales, 1996) and found that the test, although widely used, does not predict future reading ability in oral reading fluency or comprehension.

Several other assessments were also validated, with the TECOLESI test demonstrating strong correlations between PA and memory with reading ability (Capovilla, Capovilla, & Suiter, 2004) The SAL test, a computer‐based video game, also correlated with reading ability and was also described as able to reveal cognitive processing deficits in children (Reigosa‐Crespo et al., 2013).

Taken together, this set of studies on assessment validation provide a basis for thinking about how we define and assess reading outcomes in further research on EGL in the LAC region.

## 8 DISCUSSION

### 8.1 Summary of main results

This systematic review synthesized the evidence on what works to improve EGL in LAC. We also synthesized qualitative and mixed‐methods evidence to increase our understanding of the experiences and perspectives of various key stakeholders on how to improve EGL outcomes in the LAC region. Importantly, however, the evidence‐base on what works to improve EGL outcomes in the LAC region is relatively weak. We only found a small number of studies that can establish causality, and the majority of these studies have a medium or high risk of bias.

We conducted meta‐analyses on the effects of teacher training, school feeding and nutrition, and technology in education programs on EGL outcomes and a quantitative narrative synthesis on the effects of school governance, preschool, teacher practices, and parental involvement. In this narrative synthesis, we also examined the possible complementarities between teacher training and teacher coaching, the possibility of heterogeneous effects of nutrition programs in countries with low and high rates of stunting and wasting, and the separate effects of the one‐laptop‐per‐child program and other technology in education programs.

#### 8.1.1 Impact of teacher training programs

On average, we did not find statistically significant effects of teacher training on EGL outcomes, but the results suggest that teacher training programs could become more effective when they are combined with coaching. We must take care in interpreting these findings, however, because the results are only based on studies in Chile. Teacher training programs could have different effects in low‐ or middle‐income countries.

The quantitative nonintervention studies show that the quality of preschool is positively associated with EGL outcomes. Triangulating this result with the quantitative findings on the impact of teacher training suggests that teacher training combined with sustained coaching could possibly positively affect EGL outcomes through its influence on the quality of preschool.

Qualitative evidence further suggests that exemplary teachers possess a caring attitude toward their students that contributes to teachers' promotion of literacy and can potentially improve student performance (Warrican et al., 2008). These articles suggest that shifting teachers' practices and school ideologies can potentially contribute to improving education systems. However, more rigorous mixed‐methods research is needed to determine the causal mechanisms underlying these relationships. We need to exercise caution in the interpretation of the results, because the findings are only based on a small number of studies in a diverse set of contexts.

#### 8.1.2 Impact of school feeding and other nutrition programs

On average, we did not find statistically significant effects of school feeding and other nutrition programs on EGL outcomes, but we found some indications that nutrition programs may have positive effects on EGL outcomes in contexts where stunting and wasting are high, such as Guatemala. This evidence is consistent with Snilstveit et al. (2012) who show that school feeding programs can positively influence learning outcomes in low‐ and middle‐income countries. We need to exercise caution in the interpretation of the results, however, because the results are only based on a few studies, including only one study from a low‐income country (Maluccio et al., 2009). More mixed‐methods research will be needed to determine the effects of school feeding and other nutrition programs in the LAC region, particularly in low‐income countries. In addition, the effects of several included studies with an emphasis on nutrition on reading outcomes may present underestimates of the impact of these programs because of performance bias. For example, two of the studies in Jamaica are likely to underestimate the impact of nutrition programs on reading outcomes for this reason (Powell et al., 1998; Simeon et al. 1995). It will also be important to examine the potential of additional effects of nutrition programs on EGL outcomes following increases in enrollment in future reviews. For example, Snilstveit et al. (2012) show that school feeding programs have positive effects on enrollment, which can result in further improvements in EGL outcomes if the quality of education is sufficient.

Both the quantitative intervention research and the quantitative nonintervention research suggest that poverty and associated factors, such as malnutrition and child labor, are negatively associated with EGL outcomes. However, the evidence is less clear on the direction of these effects. Although poverty and reading ability are negatively correlated, the quantitative intervention studies only find evidence for a positive effect of nutrition programs in countries where the incidence of stunting and wasting is high (Maluccio et al., 2009). In other contexts, it remains unclear whether confounding factors bias the relationship between poverty and EGL outcomes.

The quantitative findings are consistent with the qualitative evidence suggesting that education programs need to be tailored to the local contexts to maximize the effectiveness of EGL programs. The evidence indicates that experiential learning or considering children's inputs in the learning process may contribute to the tailoring of education programs to the local context. In addition, extended families and social networks can also contribute to stimulating EGL outcomes. Importantly, however, we can only derive more conclusive evidence about these potential mechanisms when the number of rigorous mixed‐methods studies increases.

#### 8.1.3 Impact of technology in education programs

On average, we did not find evidence for statistically significant effects of technology in education programs on EGL outcomes. In fact, the results show some evidence for negative effects of the distribution of laptops on EGL outcomes (Cristia et al., 2012; Ferrando et al., 2011), though computer distribution programs did not show negative effects in Colombia (Barrera‐Osorio & Linden, 2009).

Qualitative evidence shows that the use of ICT may contribute to social learning if it is used for computer‐aided instruction, but our evidence also indicates that the distribution of laptops may have adverse effects if this effort is not complemented with additional interventions or programs. It is possible that computer‐aided instruction contributes to social learning, while the individualized nature of learning through using laptops may have contributed to the adverse effects. However, more rigorous mixed‐methods research is needed to assess whether ICT programs are indeed associated with reductions in social learning.

#### 8.1.4 Impact of other education programs

For the effects of preschools, school governance, specific teacher practices, and parental involvement, we only found quantitative intervention evidence with a medium or high risk of bias. These programs could potentially positively affect EGL outcomes. However, the quantitative evidence for the effectiveness of these programs in the LAC region is weak.

The four types of research suggested that most programs and implementation techniques that aim to impact EGL focus on developing PA and using read‐alouds. Both qualitative and quantitative intervention research focused on read‐aloud interventions. In Jamaica, findings suggested that read‐alouds with informational texts can help children make connections with their own realities and increase their content knowledge and expand their vocabulary (Webster, 2009). There were also indications that read‐alouds were used successfully in bilingual settings to support vocabulary acquisition in the second language (Neugebauer & Currie‐Rubin, 2009). However, the quantitative intervention research indicates that studies with an emphasis on read‐alouds have a high risk of selection bias. Furthermore, there are indications for publication bias in studies that focus on read‐alouds. Thus, we may have an incomplete picture of the influence of read‐aloud strategies on EGL outcomes. Again, more rigorous mixed‐methods research is needed to determine the effects of read‐alouds on EGL outcomes.

Quantitative nonintervention studies and qualitative intervention studies also provide evidence for a positive association between teaching phonemics, fluency, and reading comprehension. However, it is unclear whether the relationship is causal. Quantitative intervention studies do not present rigorous evidence for the positive effects of these trainings on EGL comprehension. Nonetheless, the quantitative nonintervention research suggests some interesting hypotheses on what types of programs may be effective in improving reading comprehension, which could be tested in future rigorous mixed‐methods research.

#### 8.1.5 Lack of focus on reading comprehension

There was a clear lack of studies focusing on reading comprehension. This is challenging given the fact that comprehension is the ultimate goal of reading and is something that students in the LAC region struggle to master as evidenced by scores on national reading assessments. One qualitative article focused on comprehension in very young learners and indicated that specific text and picture factors as well as teacher interaction factors affect student comprehension of stories being read aloud to them. Only three of the quantitative nonintervention studies centered on comprehension and its relationship to fluency but most studies only discussed comprehension at the word level. The quantitative intervention research on comprehension was also quite sparse. Vivas (1996) indicated that listening to stories read aloud by parents could potentially result in improvements in language comprehension and Murad and Topping (2000) found some indications for positive effects of paired reading with parents on children's reading comprehension and fluency. However, both studies have a high risk of selection‐bias, indicating that we need to exercise a lot of caution in interpreting these results.

### 8.2 Overall completeness and applicability of evidence

Overall, we only found a small number of studies that can make credible claims about the impact of development programs on EGL outcomes. The majority of the included studies suffer from either a medium or high risk of selection bias or a medium or high risk of performance bias. Furthermore, we found indications for publication bias in the studies that focus on the effects of teacher practices and parental involvement on EGL outcomes in the LAC region. These findings suggest that policy makers and other key stakeholders currently do not have access to sufficient rigorous evidence for informing their policy decisions.

In contrast to a traditional systematic review that includes only experimental and quasiexperimental quantitative research, we included all types of quantitative research as well as qualitative studies. As such, it is important to note that the included quantitative nonintervention studies do not present causal evidence on what works to improve EGL outcomes. However, these studies present some interesting hypotheses on how programs may need to be implemented to improve EGL.

### 8.3 Quality of the evidence

The accuracy of the findings from this systematic review depends on the quality of the primary studies on which the review relied. We found that both the quantitative and qualitative studies suffered from substantial limitations with respect to their quality. The results showed indications that studies with a high risk of selection‐bias or a small sample size could present upward‐biased estimates of the impact of preschool, teacher practices, and parental involvement. For this reason, we were able to present a credible meta‐analysis for only a small number of studies, and even these studies sometimes faced substantial risks of bias. We were also unable to show strong evidence of heterogeneous effects in a large sample of studies, possibly because of a lack of statistical power.

We also found evidence that the effects of teacher practices, parental involvement, and ICT programs on EGL outcomes may potentially suffer from publication bias. Our results revealed that effect sizes were higher than plausible in studies with too small sample sizes. In addition, we found some indications for publication bias in studies that focused on the effects of ICT programs on EGL outcomes.

This review is both limited and strengthened by the broad scale of the research question that guides the study. The review aims to capture every piece of research in the LAC region on EGL. Although the large scale of this research question made it difficult to search for and summarize all of the existing literature, it also enabled us to investigate larger questions within EGL.

Finally, this review uses risk of bias assessments for different research types to determine the validity and reliability of the research on EGL in the LAC region. This inclusion of different risk of bias assessments for different research types is an important strength of this review. It allows donors and policy makers to determine the quality of EGL research. Currently, the ability of policy makers to implement evidence‐based policy is compromised by the difficulties they experience in determining the quality of research. The use of risk of bias assessments enables us to assess the potential biases in the included research, which can help policy makers in determining which research findings to use and which ones to ignore.

### 8.4 Limitations and potential biases in the review process

The limitations of the review are specific to the type of research we included. We were unable to triangulate all research findings because of the relatively small number of studies eligible for the meta‐analyses when we had to rely on subsamples. In addition, the included quantitative nonintervention studies do not present causal evidence on what works to improve EGL outcomes.

#### 8.4.1 Limitations of the quantitative data analysis

##### 8.4.1.1 Lack of specific information for early grade readers

Many of the studies do not differentiate between programs that had an effect on EGL outcomes versus programs that had an effect on reading outcomes for other grades. As a result, we were not always able to make this distinction. Thus, we had to assume that the effects are homogeneous when interpreting our findings, even when this was unlikely.

##### 8.4.1.2 Publication bias

The results of our analysis may be vulnerable to publication bias. As discussed in previous sections, some of the smaller studies present effect sizes that may be overestimates, which could be an indication of publication bias (Borenstein et al., 2009). In addition, the Egger test showed some indications for publication bias in impact estimates of the ICT programs.

##### 8.4.1.3 Sample sizes

A large percentage of the papers faces limitations because of a relatively small sample size. This raises concerns because of statistical power, but also because small sample sizes may limit the ability of RCTs and quasiexperimental studies to create equivalence in observable and unobservable characteristics.

##### 8.4.1.4 Lack of cost data

Only a small percentage of the papers reports data on cost‐effectiveness. This raises some issues about the ability of the papers to provide recommendations about the scale‐up of programs that aim to improve EGL outcomes.

##### 8.4.1.5 Small number of studies

The meta‐analyses were only based on a small number of studies. As a result, the meta‐analyses may suffer from a limited statistical power to detect small but meaningful impacts of the programs. In addition, we did not have the statistical power to conduct statistical analyses using metaregressions. As a result, we had to rely on a narrative quantitative synthesis to explain differences in effect sizes across contexts and differences in effect sizes between studies that use different methods. It was also challenging to assess heterogeneity in the results.

#### 8.4.2 Limitations of the qualitative data analysis

##### 8.4.2.1 Missing information

Although the authors conducted a thorough quality assessment of each study, concerns remain that many of the qualitative studies lacked descriptions of important methodological processes. For example, although the data analysis of a study might have appeared rigorous judged by the results presented, some aspects of the research design were weak in most studies—as discussed in the quality review section. As a result, the conclusions from many qualitative studies are not reliable.

In addition, as is the case in all qualitative studies, qualitative analysis is not sufficient to determine effects on outcomes. This limitation is especially the case in the present review given the lack of reliability of the methods and the lack of the specification of outcomes up front.

### 8.5 Agreements and disagreements with other studies or reviews

On average, the review found no evidence for statistically significant effects of teacher training, school feeding and other nutrition, and technology in education programs on EGL outcomes in LAC. The narrative synthesis suggested, however, that teacher training could possibly have positive effects on EGL outcomes when it is combined with teacher coaching. In addition, the evidence indicates that nutrition programs could possibly have positive effects on EGL outcomes in low‐income countries with high rates of stunting and wasting (such as Guatemala). Furthermore, the one‐laptop‐per‐child program may have negative effects on EGL outcomes.

Although we need to exercise caution in interpreting these findings because of the small number of studies, these findings nonetheless appear to be largely in line with the recent systematic review on what works to improve education outcomes in low‐ and middle‐income countries of Snilstveit et al. (2012). They found that structured pedagogical interventions may be the among the effective approaches to improve learning outcomes in low‐ and middle‐income countries. This is consistent with our findings that teacher training is only effective in improving EGL outcomes when it is combined with teacher coaching. The finding is also consistent with our result that technology in education programs may have at best no effects unless they are combined with a focus on pedagogical practices. In line with our study, Snilstveit et al. (2012) also do not find evidence for statistically significant effects of the one‐laptop‐per‐child program. These results are consistent with the results of a meta‐analysis showing that technology in education programs are not effective when not accompanied by parent or student training (McEwan, 2015). However, neither Snilstveit et al. (2012) nor McEwan (2015) find evidence for negative effects of the one‐laptop‐per‐child program on EGL outcomes.

The impacts of school feeding and other nutrition programs on EGL outcomes are less positive than the impact of school feeding programs reported in Snilstveit et al. (2012). They find positive and statistically significant effects of school feeding on school attendance and learning outcomes in low‐ and middle‐income countries. The positive effects on school attendance may have resulted in additional effects on learning outcomes. However, we did not find statistically significant effects of nutrition programs on learning outcomes. The results of our review suggest that nutrition programs may have positive effects on EGL outcomes in the LAC region, but only in countries with high rates of stunting and wasting. However, this result is only based on one study, indicating that more research is needed on this relationship.

## 9 AUTHORS' CONCLUSIONS

### 9.1 Implications for practice and policy

Our review highlights several important implications for practice and policy related to the rollout, design, and potential impact of education programs that aim to improve EGL outcomes in the LAC region. First, our quantitative evidence suggests that teacher training, nutrition, and technology in education programs on average do not show positive effects on EGL outcomes in the LAC region. However, the quantitative narrative synthesis suggests several factors that could enable positive impacts of these programs on EGL outcomes. These factors include combining teacher training with teacher coaching, and targeting school feeding and other nutrition programs to low‐income countries with high rates of stunting and wasting. However, more research is needed for each of these factors. The current evidence‐base is not sufficient to derive strong conclusions about how combining teacher training with teacher coaching, and targeting school feeding and other nutrition programs to low‐income countries with high rates of stunting and wasting could positively affect EGL outcomes.

Second, the systematic review identified some promising opportunities for improving the design and implementation of education programs that aim to improve EGL outcomes. We found evidence for a strong correlation between PA and reading ability suggesting the need to teach PA skills early on. Studies focused on the importance of PA and phonics to help students become strong decoders. However, more research is required to establish a causal relationship between PA and reading ability.

Third, the review suggests that more resources may need to be focused on enhancing the quality of preschools through well implemented teacher training. The findings of this review suggest that such teacher training could enhance reading outcomes if the training is complemented with sustained teacher coaching. Again, however, the current evidence‐base is too small to derive strong conclusions about this relationship.

Fourth, ministries of education in low‐income countries with high rates of stunting and wasting could consider investing in programs to improve the nutrition outcomes of students in order to improve EGL outcomes. These efforts are less likely to be effective in middle‐ or high‐income countries. Again, it will remain important to build a stronger evidence‐base on this relationship; the current evidence‐base on the link between nutrition programs and EGL outcomes in the LAC region is weak.

### 9.2 Implications for research

This review has several implications for future research. First, our analysis shows the importance of ensuring that administrative data on language assessments include more than just one reading construct and differentiate between those constructs. More comprehensive administrative data will enable researchers to assess the effects of development programs on more than one EGL construct. Such an approach will enable researchers to examine the mechanisms of change in EGL outcomes at a larger scale.

Second, the medium risk of bias we found for quantitative intervention research points to a need for further investments in studies on the long‐term impacts of preschool and early childhood education strategies to determine the effectiveness of these programs.

Third, the potential of publication bias suggests a need to document ongoing research. For example, we need to ensure that when programs or interventions are not successful, the results are published and not just ignored. Unsuccessful interventions are equally important to learn from as the successful ones and if these are never publicly shared, then decision makers and practitioners alike are losing out on an important resource.

Fourth, it will be important to ensure that large‐scale research efforts use more than one reading construct. This will enable research to examine the effects of teacher practices and read alouds at a larger scale (with larger sample sizes) with lower likelihood of publication bias.

Fifth, we found major evidence‐gaps with respect to research on students with disabilities and research on prewriting and writing development. This indicates a need for more funding for research and programming that particularly tailors content to students with disabilities and research on prewriting and writing development.

Finally, the limited number of rigorous impact evaluations shows the importance of conducting more rigorous research that allows for examining the causal effects of education programs that aim to improve EGL outcomes. These studies include both experimental and quasiexperimental studies with a sufficient sample size. In addition, the studies need to be supplemented with qualitative research.

## ROLES AND RESPONSIBILITIES

- Content: R. S., T. D. H., and A. C.
- Systematic review methods: R. S., T. D. H., and A. C.
- Statistical analysis: T. D. H.
- Information retrieval: R. S. and A. C.

## SOURCES OF SUPPORT

USAID generously provided funding for this systematic review

## CONFLICT OF INTERESTS

The authors declare that there are no conflict of interests.

## PLANS FOR UPDATING THE REVIEW

We anticipate updating the review at the end of the project in 2020.

## Footnotes

1 After an initial review of a subset of citations, we refined our key indicators as needed to make them more explicit and relevant to the types of evidence we found during the search.

## APPENDIX A SEARCH STRATEGY

**Science Direct—3,748 search results**

“Read*” OR Literacy AND “primary school*” OR “primary grade*” OR {grades 1 through 3} OR {grades 1 to 3} OR {grades 1–3} OR {first through third} OR {Grade 1} OR {first grade*} OR {grade 2} OR {second grade*} OR {grade 3} OR “third grade*” OR “early grade*” OR elementary OR “kindergarten*” OR “pre‐school*” OR “preschool*” OR “pre‐kindergarten*” OR “prekindergarten*” OR preK OR “pre‐K” OR {early childhood} AND “Latin America*” OR Caribbean OR “South America*” OR {Antigua and Barbuda} OR Argentina OR Aruba OR Bahamas OR Barbados OR Belize OR Bermuda OR “Bolivia*” OR “Brazil*” OR “British Virgin Islands” OR “Cayman Islands” OR “Chile*” OR “Colombia*” OR “Costa Rica*” OR “Cuba*” OR Curacao OR “Dominica*” OR “Dominican Republic” OR “Ecuador*” OR “El Salvador*” OR “French Guiana*” OR “Grenada*” OR Guadeloupe OR “Guatemala*” OR “Guyana*” OR “Haiti*” OR Honduras OR “Jamaica*” OR Martinique OR Mexico OR Mont Serrat OR “Netherlands Antilles” OR “Nicaragua*” OR “Panama*” OR “Paraguay*” OR “Peru*” OR “Puerto Rico” OR “Saint Barthelemy” OR “Saint Kitts and Nevis” OR “Saint Lucia*” OR “Saint‐Martin” OR {Saint Vincent and the Grenadines} OR “Sint Maarten” OR Suriname OR {Trinidad and Tobago} OR {Turks and Caicos} OR Uruguay OR {Virgin Islands} OR Venezuela

According to the rules of Science Direct, a phrase must be enclosed in {} to ensure that the phrase is exact, and includes stop words. We enclosed only those phrases with stop words. Date range: 1990–2016

2. Removed all asterisks and added parentheses between the three components to ensure proper order of operations.

(“Read” OR Literacy) AND (“primary school” OR “primary grade” OR {grades 1 through 3} OR {grades 1 to 3} OR {grades 1–3} OR {first through third} OR {Grade 1} OR {first grade} OR {grade 2} OR {second grade} OR {grade 3} OR “third grade” OR “early grade” OR elementary OR “kindergarten” OR “pre‐school” OR “preschool” OR “pre‐kindergarten” OR “prekindergarten” OR preK OR “pre‐K” OR {early childhood}) AND (“Latin America” OR Caribbean OR “South America” OR {Antigua and Barbuda} OR Argentina OR Aruba OR Bahamas OR Barbados OR Belize OR Bermuda OR “Bolivia” OR “Brazil” OR “British Virgin Islands” OR “Cayman Islands” OR “Chile” OR “Colombia” OR “Costa Rica” OR “Cuba” OR Curacao OR “Dominica” OR “Dominican Republic” OR “Ecuador” OR “El Salvador” OR “French Guiana” OR “Grenada” OR Guadeloupe OR “Guatemala” OR “Guyana” OR “Haiti” OR Honduras OR “Jamaica” OR Martinique OR Mexico OR Mont Serrat OR “Netherlands Antilles” OR “Nicaragua” OR “Panama” OR “Paraguay” OR “Peru” OR “Puerto Rico” OR “Saint Barthelemy” OR {Saint Kitts and Nevis} OR “Saint Lucia” OR “Saint‐Martin” OR {Saint Vincent and the Grenadines} OR “Sint Maarten” OR Suriname OR {Trinidad and Tobago} OR {Turks and Caicos} OR Uruguay OR {Virgin Islands} OR Venezuela)

3. It was not immediately clear that the relevant articles yielded on the first search were also present in the second search, so we selected a few relevant articles from the first set of results to look for within the second set of results. We found these same articles within the second set of results, so the smaller number of results also include the relevant articles yielded from the first entry.

**Results: 2,053**

**SAGE**

“early grade” AND literacy (all fields)

OR “early grade” AND reading (all fields)

OR childhood AND reading (all fields)

OR childhood AND literacy (all fields)

AND South America OR Latin America (all fields)

OR Caribbean OR Central America (all fields)

From Jan 1990 through Jan 2016

**Table:**

**Method 1: Manually selected disciplines (4,680 results)**	
Education	
Ethnic Studies	
Family Studies	
Gender Studies	
Group Studies	
Language and Linguistics	
Regional Studies	
Research Methods and Evaluation	
Special Education	
**Method 2: Manually selected Sage journals included (964 results)**	
American Educational Research Journal	
Australian Journal of Education	
Child Language Teaching and Therapy	
Childhood: A journal of global child research	
Contemporary Education Dialogue	
Contemporary Issues in Early Childhood	
Education and Urban Society	
Education, Citizenship, and Social Justice	
Educational Administration Quarterly: The Journal of Leadership for Effective and Equitable Organizations	
Educational Evaluations and Policy Analysis	
Educational Horizons	
Educational Policy: An Interdisciplinary Journal of Policy and Practice	
Educational Researcher	
European Educational Research Journal	
Exceptional Children	
Gifted Children Quarterly	
Gifted Child Today	
Gifted Education International	
Global Studies of Childhood	
International Journal of Christianity and Education	
Journal for the Education of the Gifted	
Journal of Early Childhood Literacy	
Journal of Early Childhood Research	
Journal of Education for Sustainable Development	
Journal of Educational and Behavioral Statistics	
Journal of Experiential Education	
Journal of Literacy Research	
Journal of Planning Education and Research	
Journal of Research in International Education	
The Journal of Special Education	
Journal of Studies in International Education	
Journal of Transformative Education	
Language and Linguistics	
Language and Literature	
Language and Speech	
Language Teaching Research	
Management in Education	
Power and Education	
Remedial and Special Education	
Research in Comparative and International Education	
Review of Educational Research	
Review of Research in Education	
Sociology of Education	
Teacher Education and Special Education: The Journal of the Teacher Education Division of the Council for Exceptional Children	
TEACHING Exceptional Children	
Theory and Research in Education	
Topics in Early Childhood Special Education	
Urban Education	
Young	
Young Exceptional Children	
Youth and Society	

**Taylor & Francis—3,442 results**

(“early childhood” OR “early grade” AND Read OR Literacy) AND (“Latin America” OR Caribbean OR “South America” OR “Central America”)

From Jan 1990 through Jan 2016

**Subject Areas**

Education

Language and Literature

Note: We purposely excluded other subject areas such as “Latin American Studies” because they yielded irrelevant results.

Updated search terms as follows:

(“early childhood” OR “early grade”) AND (Read* OR Literacy) AND (“Latin America” OR Caribbean OR “South America” OR “Central America”)

Added date parameters, but did not need to add additional subject area limitations

**1,258 results**

**JSTOR**

Original search string was too long to accept. The number of characters is limited across seven fields.

By entering the search terms manually, as follows, we got over a million results:

read* OR literacy

AND “early grade”

OR “early child”

AND “Latin America*”

OR Caribbean

OR “South America*”

Within the search engine, the logic structure was depicted as follows:

((((((Read* OR Literacy) AND (“early grade*”)) OR (“early child*”)) AND (Latin America*)) OR (Caribbean)) OR (South America*))

We changed this structure according to the Boolean logic structure, which is as follows:

(Read* OR Literacy) AND ((“early grade*”) OR (“early child*”)) AND ((Latin America*) OR (Caribbean) OR (South America*)) **Results: 2,801**

I removed the parentheses and quotation marks to determine relevancy and number of articles and found that quotation marks are necessary to keep phrases together.

(Read* OR Literacy) AND ((“early grade*”) OR (“early child*”)) AND ((“Latin America*”) OR (Caribbean) OR (“South America*”)) **Results: 258**

(Read* OR Literacy) AND (“early grade*” OR “early child*”) AND (“Latin America*” OR Caribbean OR “South America*”) **Results: 258**

(Read* OR Literacy) AND ((early grade*) OR (early child*)) AND ((Latin America*) OR (Caribbean) OR (South America*)) **Results: 588,645**

Parentheses are good for ordering, while quotation marks are good for phrases, even with the * included for variation.

We added the term “primary grade” to include more relevant results

(Read* OR Literacy) AND (“early grade*” OR “early child*” OR “primary grade”) AND (Latin America* OR Caribbean OR South America*)

**Results: 3,652**

I removed all asterisks from phrases:

(Read* OR Literacy) AND (“early grade” OR “early child” OR “primary grade”) AND (“Latin America” OR Caribbean OR “South America”)

Some relevant results, but mostly not – eliminated some relevant results in previous search

**Results: 336**

**Table:**

**EBSCO**
**Databases searched:**
ERIC
Academic Search Premier
Education Source
PsycINFO
CINAHL
Psychology and Behavioral Sciences Collection
Collection
SocINDEX with Full Text
EconLit

The original search string was used in the format provided.

Read* OR Literacy

AND

“primary school*” OR “primary grade*” OR “grades 1 through 3” OR “grades 1 to 3” OR “grades 1–3” OR “first through third” OR “Grade 1” OR “first grade*” OR “grade 2” OR “second grade*” OR “grade 3” OR “third grade*” OR “early grade*” OR elementary OR kindergarten* OR pre‐school* OR preschool* OR pre‐kindergarten* OR prekindergarten* OR preK OR pre‐K OR “early childhood”

AND

“Latin America*” OR Caribbean OR “South America*” OR Antigua* and Barbuda OR Argentina OR Aruba OR Bahamas OR Barbados OR Belize OR Bermuda OR Bolivia* OR Brazil* OR “British Virgin Islands” OR “Cayman Islands” OR Chile* OR Colombia* OR “Costa Rica*” OR Cuba* OR Curacao OR Dominica* OR “Dominican Republic” OR Ecuador* OR “El Salvador*” OR “French Guiana*” OR Grenada* OR Guadeloupe OR Guatemala* OR Guyana* OR Haiti* OR Honduras OR Jamaica* OR Martinique OR Mexico OR Mont Serrat OR “Netherlands Antilles” OR Nicaragua* OR Panama* OR Paraguay* OR Peru* OR “Puerto Rico” OR “Saint Barthelemy” OR “Saint Kitts and Nevis” OR “Saint Lucia*” OR “Saint‐Martin” OR “Saint Vincent and the Grenadines” OR “Sint Maarten” OR Suriname OR Trinidad and Tobago OR “Turks and Caicos” OR Uruguay OR “Virgin Islands” OR Venezuela

From 1990 to 2015

**Results: 2,779**

Modified research results have removed asterisks that are within quotes, and is written as follows:

Read* OR Literacy

AND

“primary school” OR “primary grade” OR “grades 1 through 3” OR “grades 1 to 3” OR “grades 1–3” OR “first through third” OR “Grade 1” OR “first grade” OR “grade 2” OR “second grade” OR “grade 3” OR “third grade” OR “early grade” OR elementary OR kindergarten* OR pre‐school* OR preschool* OR pre‐kindergarten* OR prekindergarten* OR preK OR pre‐K OR “early childhood”

AND

“Latin America” OR Caribbean OR “South America” OR Antigua* and Barbuda OR Argentina OR Aruba OR Bahamas OR Barbados OR Belize OR Bermuda OR Bolivia* OR Brazil* OR “British Virgin Islands” OR “Cayman Islands” OR Chile* OR Colombia* OR “Costa Rica” OR Cuba* OR Curacao OR Dominica* OR “Dominican Republic” OR Ecuador* OR “El Salvador” OR “French Guiana” OR Grenada* OR Guadeloupe OR Guatemala* OR Guyana* OR Haiti* OR Honduras OR Jamaica* OR Martinique OR Mexico OR Mont Serrat OR “Netherlands Antilles” OR Nicaragua* OR Panama* OR Paraguay* OR Peru* OR “Puerto Rico” OR “Saint Barthelemy” OR “Saint Kitts and Nevis” OR “Saint Lucia” OR “Saint‐Martin” OR “Saint Vincent and the Grenadines” OR “Sint Maarten” OR Suriname OR Trinidad and Tobago OR “Turks and Caicos” OR Uruguay OR “Virgin Islands” OR Venezuela

**Results: 2,612**

**Cochrane**

This is a medical database that is part of Wiley Online journal. See Wiley for explanation.

**Wiley**

Tried entering the original string, response said, “search terms should be more than 1 characters long”

Tried entering into the smaller search engine, but the database could not handle computing the command

Entered:

(“Read*” OR Literacy) AND (“primary school*” OR “primary grade*” OR “grades 1 through 3” OR “grades 1 to 3” OR “grades 1–3” OR “first through third” OR “Grade 1” OR “first grade*” OR “grade 2” OR “second grade*” OR “grade 3” OR “third grade*” OR “early grade*” OR elementary OR “kindergarten*” OR “preschool*” OR “prekindergarten*” OR preK OR “early childhood”) AND (“Latin America*” OR Caribbean OR “South America*” OR “Central America*”)

**Results: 2,580,083**

A mix of relevant and irrelevant results.

We tried again by entering the same string but selected “full text” for the fields. Excessive and irrelevant results. We tried “abstract” with excessive and irrelevant results.

We tried a new string:

(“Read*” OR Literacy) AND (“primary school*” OR “primary grade*” OR “early grade*” OR elementary OR “kindergarten*” OR “preschool*” OR “prekindergarten*” OR preK OR “early childhood”) AND (“Latin America*” OR Caribbean OR “South America*” OR “Central America*”)

**Results: 12,962**

Irrelevant results.

To weed out irrelevant results, we tried adding NOT psychology* NOT disease*

(“Read*” OR Literacy) AND (“primary school*” OR “primary grade*” OR “early grade*” OR elementary OR “kindergarten*” OR “preschool*” OR “prekindergarten*” OR preK OR “early childhood”) AND (“Latin America*” OR Caribbean OR “South America*” OR “Central America*”) NOT psycholog* NOT disease*

**Results: 3,540**

These articles seem relevant.

We removed quotation marks on one‐word entries, and asterisks from phrases. We also added an asterisk *before* kindergarten to account for prekindergarten.

(Read* OR Literacy) AND (“primary school” OR “primary grade” OR “early grade” OR elementary OR *kindergarten* OR preschool* OR preK OR “early childhood”) AND (“Latin America” OR Caribbean OR “South America” OR “Central America”) NOT psycholog* NOT disease*

**Results: 2,390**

Checked to see if the same relevant articles that appeared in entry from step #6 appeared for the entry from step #7, confirmed availability.

**Table:**

**ProQuest**
Signed up for a free trial and was limited to six journals, selected the following journals:
Australian Education Index
CBCA Education
ERIC
Linguistics and Language Behavior Abstracts
Proquest Learning: Literature
Proquest Education Journals

Got confirmation and notification that they will email me more information about this free trial in a few days

No information as of 7/20

**The Campbell Library**

Entered the string as follows:

(Read* OR Literacy)

AND

(“primary school*” OR “primary grade*” OR “grades 1 through 3” OR “grades 1 to 3” OR “grades 1–3” OR “first through third” OR “Grade 1” OR first grade* OR “grade 2” OR second grade* OR “grade 3” OR third grade* OR early grade* OR elementary OR kindergarten* OR pre‐school* OR preschool* OR pre‐kindergarten* OR prekindergarten* OR preK OR pre‐K OR “early childhood”)

AND

(Latin America* OR Caribbean OR South America* OR Antigua* and Barbuda OR Argentina OR Aruba OR Bahamas OR Barbados OR Belize OR Bermuda OR Bolivia* OR Brazil* OR “British Virgin Islands” OR “Cayman Islands” OR Chile* OR Colombia* OR Costa Rica* OR Cuba* OR Curacao OR Dominica* OR “Dominican Republic” OR Ecuador* OR El Salvador* OR French Guiana* OR Grenada* OR Guadeloupe OR Guatemala* OR Guyana* OR Haiti* OR Honduras OR Jamaica* OR Martinique OR Mexico OR Mont Serrat OR “Netherlands Antilles” OR Nicaragua* OR Panama* OR Paraguay* OR Peru* OR “Puerto Rico” OR “Saint Barthelemy” OR “Saint Kitts and Nevis” OR Saint Lucia* OR “Saint‐Martin” OR “Saint Vincent and the Grenadines” OR “Sint Maarten” OR Suriname OR Trinidad and Tobago OR “Turks and Caicos” OR Uruguay OR “Virgin Islands” OR Venezuela)

We modified the string to enclose all the countries with “and” in their names

(Read* OR Literacy)

AND

(“primary school*” OR “primary grade*” OR “grades 1 through 3” OR “grades 1 to 3” OR “grades 1–3” OR “first through third” OR “Grade 1” OR first grade* OR “grade 2” OR second grade* OR “grade 3” OR third grade* OR early grade* OR elementary OR kindergarten* OR pre‐school* OR preschool* OR pre‐kindergarten* OR prekindergarten* OR preK OR pre‐K OR “early childhood”)

AND

(Latin America* OR Caribbean OR South America* OR Antigua* and Barbuda OR Argentina OR Aruba OR Bahamas OR Barbados OR Belize OR Bermuda OR Bolivia* OR Brazil* OR “British Virgin Islands” OR “Cayman Islands” OR Chile* OR Colombia* OR “Costa Rica*” OR Cuba* OR Curacao OR Dominica* OR “Dominican Republic” OR Ecuador* OR “El Salvador*” OR “French Guiana*” OR Grenada* OR Guadeloupe OR Guatemala* OR Guyana* OR Haiti* OR Honduras OR Jamaica* OR Martinique OR Mexico OR Mont Serrat OR “Netherlands Antilles” OR Nicaragua* OR Panama* OR Paraguay* OR Peru* OR “Puerto Rico” OR “Saint Barthelemy” OR “Saint Kitts and Nevis” OR “Saint Lucia*” OR “Saint‐Martin” OR “Saint Vincent and the Grenadines” OR “Sint Maarten” OR Suriname OR “Trinidad and Tobago” OR “Turks and Caicos” OR Uruguay OR “Virgin Islands” OR Venezuela)

Since the earliest publication goes back to 2003, we did not need to make additional date adjustments. Results look relevant. **Results: 217**

Updated entries to remove all asterisks within quotes

(Read* OR Literacy)

AND

(“primary school” OR “primary grade” OR “grades 1 through 3” OR “grades 1 to 3” OR “grades 1–3” OR “first through third” OR “Grade 1” OR first grade* OR “grade 2” OR second grade* OR “grade 3” OR third grade* OR early grade* OR elementary OR kindergarten* OR pre‐school* OR preschool* OR pre‐kindergarten* OR prekindergarten* OR preK OR pre‐K OR “early childhood”)

AND

(“Latin America” OR Caribbean OR “South America” OR Antigua* and Barbuda OR Argentina OR Aruba OR Bahamas OR Barbados OR Belize OR Bermuda OR Bolivia* OR Brazil* OR “British Virgin Islands” OR “Cayman Islands” OR Chile* OR Colombia* OR “Costa Rica” OR Cuba* OR Curacao OR Dominica* OR “Dominican Republic” OR Ecuador* OR “El Salvador” OR “French Guiana” OR Grenada* OR Guadeloupe OR Guatemala* OR Guyana* OR Haiti* OR Honduras OR Jamaica* OR Martinique OR Mexico OR Mont Serrat OR “Netherlands Antilles” OR Nicaragua* OR Panama* OR Paraguay* OR Peru* OR “Puerto Rico” OR “Saint Barthelemy” OR “Saint Kitts and Nevis” OR “Saint Lucia” OR “Saint‐Martin” OR “Saint Vincent and the Grenadines” OR “Sint Maarten” OR Suriname OR “Trinidad and Tobago” OR “Turks and Caicos” OR Uruguay OR “Virgin Islands” OR Venezuela)

No hits! We backtracked, and step 2 also yielded no hits! We updated the quotation marks to reflect Unicode, and yielded eight hits. Then we again removed all asterisks within quotation marks, as follows. This also yielded eight hits.

(Read* OR Literacy)

AND

(“primary school” OR “primary grade” OR “grades 1 through 3” OR “grades 1 to 3” OR “grades 1–3” OR “first through third” OR “Grade 1” OR first grade* OR “grade 2” OR second grade* OR “grade 3” OR third grade* OR early grade* OR elementary OR kindergarten* OR pre‐school* OR preschool* OR pre‐kindergarten* OR prekindergarten* OR preK OR pre‐K OR “early childhood”)

AND

(“Latin America” OR Caribbean OR “South America” OR Antigua* and Barbuda OR Argentina OR Aruba OR Bahamas OR Barbados OR Belize OR Bermuda OR Bolivia* OR Brazil* OR “British Virgin Islands” OR “Cayman Islands” OR Chile* OR Colombia* OR “Costa Rica” OR Cuba* OR Curacao OR Dominica* OR “Dominican Republic” OR Ecuador* OR “El Salvador” OR “French Guiana” OR Grenada* OR Guadeloupe OR Guatemala* OR Guyana* OR Haiti* OR Honduras OR Jamaica* OR Martinique OR Mexico OR Mont Serrat OR “Netherlands Antilles” OR Nicaragua* OR Panama* OR Paraguay* OR Peru* OR “Puerto Rico” OR “Saint Barthelemy” OR “Saint Kitts and Nevis” OR “Saint Lucia” OR “Saint‐Martin” OR “Saint Vincent and the Grenadines” OR “Sint Maarten” OR Suriname OR “Trinidad and Tobago” OR “Turks and Caicos” OR Uruguay OR “Virgin Islands” OR Venezuela)

Search “help” only says, to use an asterisk to search for multiple characters after a search strings, so we removed all quotes, which yielded no hits. Then we entered it as follows (enclosing phrases in quotes, except those with asterisks):

Read* OR Literacy

AND

“primary school” OR “primary grade” OR “grades 1 through 3” OR “grades 1 to 3” OR “grades 1–3” OR “first through third” OR “Grade 1” OR first grade* OR “grade 2” OR second grade* OR “grade 3” OR third grade* OR early grade* OR elementary OR kindergarten* OR pre‐school* OR preschool* OR *kindergarten* OR preK OR pre‐K OR “early childhood”

AND

Latin America* OR Caribbean OR South America* OR “Antigua and Barbuda” OR Argentina OR Aruba OR Bahamas OR Barbados OR Belize OR Bermuda OR Bolivia* OR Brazil* OR “British Virgin Islands” OR “Cayman Islands” OR Chile* OR Colombia* OR “Costa Rica” OR Cuba* OR Curacao OR Dominica* OR “Dominican Republic” OR Ecuador* OR “El Salvador” OR “French Guiana” OR Grenada* OR Guadeloupe OR Guatemala* OR Guyana* OR Haiti* OR Honduras OR Jamaica* OR Martinique OR Mexico OR “Mont Serrat” OR “Netherlands Antilles” OR Nicaragua* OR Panama* OR Paraguay* OR Peru* OR “Puerto Rico” OR “Saint Barthelemy” OR “Saint Kitts and Nevis” OR “Saint Lucia” OR “Saint‐Martin” OR “Saint Vincent and the Grenadines” OR “Sint Maarten” OR Suriname OR “Trinidad and Tobago” OR “Turks and Caicos” OR Uruguay OR “Virgin Islands” OR Venezuela

**Results: 189**

**Dissertation Abstracts**

This is part of Proquest. Due to limited access to Proquest via free trial subscriptions, we cannot access this.

**Directory of Open Access Journals (DOAJ)**

Entered original search string, yielded zero results

Eliminated the countries, yielded zero results

Entered (“read*” OR literacy) AND (“early grade” OR childhood) AND (“South America” OR “Latin America” OR “Central America” OR Caribbean), yielded 93 results, mixed results

Tried filtering, but it eliminated some relevant results

Does not allow for date restrictions, but all the articles are recent

**Results: 94**

Modified the entry to be as follows (removed quotes from “read”):

(read* OR literacy) AND (“early grade” OR childhood) AND (“South America” OR “Latin America” OR “Central America” OR Caribbean)

Did not make a difference in search results. Both entries (#3 and #6) work, and yield the same results.

**Directory of Open Access Books (DOAB)**

Entered original search string into “simple search,” yielded 104 irrelevant results

Entered the same search string into “advanced search,” yielded 10 irrelevant results

Entered modified search string into “advanced search” as follows:

(Read* OR all:Literacy) AND (“primary all:school*” OR all:“primary OR all:”grades OR all:“grades OR all:”grades OR all:“first OR all:”Grade OR all:first all:grade* OR all:“grade OR all:second all:grade* OR all:”grade OR all:third all:grade* OR all:early all:grade* OR all:elementary OR all:kindergarten* OR all:pre‐school* OR all:preschool* OR all:pre‐kindergarten* OR all:prekindergarten* OR all:preK OR all:pre‐K OR all:“early childhood” all:)

AND

(all:(“South all:America” OR all:“Latin OR all:”Central OR all:Caribbean))

Yielded 17 results that were irrelevant. It appears this database does not have any relevant results.

**3ie**

Cannot fit original search string into search engine

Modified search string and got one irrelevant result:

(“Read*” OR Literacy) AND (“primary school*” OR “primary grade*” OR “early grade”) AND (“South America*” OR “Latin America*” OR “Central America*” OR Caribbean)

Modified search string and got 3 irrelevant results:

(Read OR Literacy) AND (primary school OR primary grade OR early grade) AND (South America OR Latin America OR Central America OR Caribbean)

Modified search string and got six results, only one of which was relevant:

(Read OR Literacy) AND (primary school OR primary grade OR early grade OR childhood) AND (South America OR Latin America OR Central America OR Caribbean)

Link to relevant article: http://www.scielo.br/scielo.php?pid=S0101‐41612012000100004&script=sci_arttext

To further ensure the accuracy of these results, we experimented and tried entering string #2, but with the quotes removed from “read.” We also got irrelevant results.

**International Bibliography of the Social Sciences**

See Proquest

**British Library for Development Studies**

Entered the results as follows, with no results

(Read* OR Literacy)

AND

(“primary school*” OR “primary grade*” OR “grades 1 through 3” OR “grades 1 to 3” OR “grades 1–3” OR “first through third” OR “Grade 1” OR first grade* OR “grade 2” OR second grade* OR “grade 3” OR third grade* OR early grade* OR elementary OR kindergarten* OR pre‐school* OR preschool* OR pre‐kindergarten* OR prekindergarten* OR preK OR pre‐K OR “early childhood”)

AND

(“Latin America*” OR Caribbean OR “South America*” OR Antigua* and Barbuda OR Argentina OR Aruba OR Bahamas OR Barbados OR Belize OR Bermuda OR Bolivia* OR Brazil* OR “British Virgin Islands” OR “Cayman Islands” OR Chile* OR Colombia* OR “Costa Rica*” OR Cuba* OR Curacao OR Dominica* OR “Dominican Republic” OR Ecuador* OR “El Salvador*” OR “French Guiana*” OR Grenada* OR Guadeloupe OR Guatemala* OR Guyana* OR Haiti* OR Honduras OR Jamaica* OR Martinique OR Mexico OR Mont Serrat OR “Netherlands Antilles” OR Nicaragua* OR Panama* OR Paraguay* OR Peru* OR “Puerto Rico” OR “Saint Barthelemy” OR “Saint Kitts and Nevis” OR “Saint Lucia*” OR “Saint‐Martin” OR “Saint Vincent and the Grenadines” OR “Sint Maarten” OR Suriname OR “Trinidad and Tobago” OR “Turks and Caicos” OR Uruguay OR “Virgin Islands” OR Venezuela)

Cut down thread, and entered the following with no results:

(“Read*” OR Literacy) AND (“primary school*” OR “primary grade*” OR “early grade”) AND (“South America*” OR “Latin America*” OR “Central America*” OR Caribbean)

Entered “early childhood reading” with no results

Entered “EGL” with no results

Entered “child literacy” with 39 irrelevant results

This journal has no relevant results.

Based on not finding results for #3–5, we will not try removing asterisks

**Table:**

**Education International**
This search engine allows you to choose a region, so we chose Latin America, which yielded 189 results. We unchecked the following for types of resources, which reduced the results to 48:
News
Events
Urgent Action Appeals
I unchecked the following for subject matter, got 18 results that were not relevant:
About EI
Trade & Education
Higher Education & Research
HIV/AIDS
Human & Trade Union Rights
Professional Ethics
Sexual Orientation
Health and Safety in Schools
Solidarity Fund
Migrant Rights
Racism and Xenophobia
Economic Crisis
Congress 7
I selected all options again, and tried:
Entering “reading” and “literacy” but with no results
Entering “early” with 2 irrelevant results
I chose another region, North America‐Caribbean, 375 irrelevant results
Entered “reading” and “literacy,” the latter yielding 5 irrelevant results
Entered “early” with 8 irrelevant results
I didn't find anything useful here. Zero relevant results.

**Google Scholar**

Couldn't enter original search string due to character limit

Entered with 311,000 results:

(“Read*” OR Literacy) AND (“primary school*” OR “primary grade*” OR “early grade”) AND (“South America*” OR “Latin America*” OR “Central America*” OR Caribbean)

Added *date parameters* and got 17,800 results

Removed quotation marks on all except “early grade” and got 18,800 results, mixed relevance

(Read* OR Literacy) AND (primary school* OR primary grade* OR “early grade”) AND (South America* OR Latin America* OR Central America* OR Caribbean)

Modified results as follows, for **20,800** results:

(“EGL” OR “EGL” OR (“early childhood” AND (reading OR literacy)) AND (Latin America OR South America OR Central America OR Caribbean)

The results seem relevant, even after skipping several pages of results.

Tried adding “NOT” to make results more relevant

(“EGL” OR “EGL” OR (“early childhood” AND (reading OR literacy)) AND (Latin America OR South America OR Central America OR Caribbean) NOT mathematics

I did not any further edits to this search engine because the team decided not to use this search engine.

**HAPI**

This search engine required a subscription that we do not have.

**LANIC**

Original search string did not yield any results.

Removed all numbered grade references, did not yield any results either.

Removed all country references, since this is a database on Latin America. No results.

Entered results as follows:

(Read OR Literacy) AND (“primary school” OR “primary grade” OR “early grade” OR elementary OR kindergarten OR preschool OR “early childhood”)

Mixed results, **results: 208**

Added asterisks as follows:

(Read* OR Literacy) AND (“primary school” OR “primary grade” OR “early grade” OR elementary OR kindergarten* OR preschool* OR “early childhood”)

No results. If we leave asterisk only on “read,” it yields **187 results**. These results are mixed. It seems that the articles are among the results because a teacher provides a narrative of what they do: “We teach high school students who read English on a primary‐grade level” for an article entitled “Animals of Ecuador and Virginia.”

(Read* OR Literacy) AND (“primary school” OR “primary grade” OR “early grade” OR elementary OR kindergarten OR preschool OR “early childhood”)

Removed quotation marks, got 162 results, but less relevant.

**DEC**

This is a Google powered engine, and just as we could not enter the original search string in Google, we cannot do so here either. So we borrowed from my first string in Google, but modified to remove asterisks from phrases, and quotation marks from single words.

(Read* OR Literacy) AND (“primary school” OR “primary grade” OR “early grade”) AND (“South America” OR “Latin America” OR “Central America” OR Caribbean)

**Results: 3910**

Within the date parameters, there are **2,231 results**. However, the results must be filtered in categories. For dates, the results are filtered by decade: 1990–1999 (1,028 results), 2000–2009 (860 results), and 2010 or later (343 results)

**WorldCat**

Entered original search string, with date parameters of 1990–2016, into one field:

(“Read” OR Literacy) AND (“primary school” OR “primary grade” OR “grades 1 through 3” OR “grades 1 to 3” OR “grades 1–3” OR “first through third” OR “Grade 1” OR “first grade” OR “grade 2” OR “second grade” OR “grade 3” OR “third grade” OR “early grade” OR elementary OR “kindergarten” OR “pre‐school” OR “preschool” OR “pre‐kindergarten” OR “prekindergarten” OR preK OR “pre‐K” OR “early childhood”) AND (“Latin America” OR Caribbean OR “South America” OR “Antigua and Barbuda” OR Argentina OR Aruba OR Bahamas OR Barbados OR Belize OR Bermuda OR “Bolivia” OR “Brazil” OR “British Virgin Islands” OR “Cayman Islands” OR “Chile” OR “Colombia” OR “Costa Rica” OR “Cuba” OR Curacao OR “Dominica” OR “Dominican Republic” OR “Ecuador” OR “El Salvador” OR “French Guiana” OR “Grenada” OR Guadeloupe OR “Guatemala” OR “Guyana” OR “Haiti” OR Honduras OR “Jamaica” OR Martinique OR Mexico OR Mont Serrat OR “Netherlands Antilles” OR “Nicaragua” OR “Panama” OR “Paraguay” OR “Peru” OR “Puerto Rico” OR “Saint Barthelemy” OR “Saint Kitts and Nevis” OR “Saint Lucia” OR “Saint‐Martin” OR “Saint Vincent and the Grenadines” OR “Sint Maarten” OR Suriname OR “Trinidad and Tobago” OR “Turks and Caicos” OR Uruguay OR “Virgin Islands” OR Venezuela)

Yielded system error, so we divided the string by three:

(“Read” OR Literacy)

AND

(“primary school” OR “primary grade” OR “grades 1 through 3” OR “grades 1 to 3” OR “grades 1–3” OR “first through third” OR “Grade 1” OR “first grade” OR “grade 2” OR “second grade” OR “grade 3” OR “third grade” OR “early grade” OR elementary OR “kindergarten” OR “pre‐school” OR “preschool” OR “pre‐kindergarten” OR “prekindergarten” OR preK OR “pre‐K” OR “early childhood”)

AND

(“Latin America” OR Caribbean OR “South America” OR “Antigua and Barbuda” OR Argentina OR Aruba OR Bahamas OR Barbados OR Belize OR Bermuda OR “Bolivia” OR “Brazil” OR “British Virgin Islands” OR “Cayman Islands” OR “Chile” OR “Colombia” OR “Costa Rica” OR “Cuba” OR Curacao OR “Dominica” OR “Dominican Republic” OR “Ecuador” OR “El Salvador” OR “French Guiana” OR “Grenada” OR Guadeloupe OR “Guatemala” OR “Guyana” OR “Haiti” OR Honduras OR “Jamaica” OR Martinique OR Mexico OR Mont Serrat OR “Netherlands Antilles” OR “Nicaragua” OR “Panama” OR “Paraguay” OR “Peru” OR “Puerto Rico” OR “Saint Barthelemy” OR “Saint Kitts and Nevis” OR “Saint Lucia” OR “Saint‐Martin” OR “Saint Vincent and the Grenadines” OR “Sint Maarten” OR Suriname OR “Trinidad and Tobago” OR “Turks and Caicos” OR Uruguay OR “Virgin Islands” OR Venezuela)

System error. We removed the parentheses, got 64,126 hits. Even though the “help” section talks about using parentheses to create more precise searches, we get error responses (http://www.oclc.org/support/help/navpatron/ApplicationHelp.htm).

Added date parameters for 1990–2016, got 38,300 hits. After looking through the results, we did not find relevant results.

Modified search string to remove all the “grade 1” “grade 2” and “grade 3” references, got the same number of results

Removed all references to grade (i.e., kindergarten, preschool), still got the same number of results.

Removed all country references, to focus on regional, and got 11,213 results:

Read* OR Literacy

AND

“primary school” OR “primary grade” OR “early grade” OR “early childhood”

AND

“Latin America” OR Caribbean OR “South America” OR “Central America”

Tried further filtering topics by clicking on the topic of “Education” and got 875 results, but the results included titles clearly irrelevant to the subject, that is, “Examining the impacts of dynamic downscaling method and vegetation biophysical processes on the South American regional climate simulation” and some that were tangentially relevant, “The experiences of African, Caribbean and south Asian women in initial teacher education”

Tried entering data into the fields, one by one, got 15,559 irrelevant results:

kw:Read* OR kw:Literacy AND kw:primary school OR kw:primary grade OR kw:early grade OR kw:elementary OR kw:*kindergarten* OR kw:preschool* OR kw:prek OR kw:early childhood AND kw:Latin America* OR kw:Central America* OR kw:South America* OR kw:Caribbean AND yr:1990..2016

Filtered by education, got 1,408 results with irrelevant results, for example: “The determinants of remittances: Latin America and the Caribbean, 1982–2001” and “Taxonomy of larval blennioidei of Belize, Central America.”

If we go through the results, we find a few potentially relevant results (although it is not immediately clear upon reading the title).

Backtracked to step 2 (without using parentheses) and removed quotations from single words such as “Venezuela” and “Brazil,” and still got irrelevant results (3,542 results).

Filtered out by “Education,” irrelevant results (194 results). Removed the following fields: “Individual Institutions,” “Higher Education,” and “Individual Institutions—America—Except U.S.” got 37 results.

Backtracked again to step 2 (without using parentheses), and tried the same entry WITH quotations, and repeated step 12, with mixed results.

Backtracked to step 7, removed all quotation marks, and filtered according to step 12 (98 results). Even though the results are related to Education, they are not specifically related to EGL.

Backtracked again to step 1, and removed parentheses and quotation marks on one‐word entries. Date parameters set to 1990–2016.

Entered as follows:

Read* OR Literacy

AND

“primary school” OR “primary grade” OR “grades 1 through 3” OR “grades 1 to 3” OR “grades 1–3” OR “first through third” OR “Grade 1” OR “first grade” OR “grade 2” OR “second grade” OR “grade 3” OR “third grade” OR “early grade” OR elementary OR kindergarten OR “pre‐school” OR “preschool” OR “pre‐kindergarten” OR “prekindergarten” OR preK OR “pre‐K” OR “early childhood”

AND

“Latin America” OR Caribbean OR “South America” OR “Antigua and Barbuda” OR Argentina OR Aruba OR Bahamas OR Barbados OR Belize OR Bermuda OR Bolivia OR Brazil OR “British Virgin Islands” OR “Cayman Islands” OR Chile OR Colombia OR “Costa Rica” OR Cuba OR Curacao OR Dominica OR “Dominican Republic” OR Ecuador OR “El Salvador” OR “French Guiana” OR Grenada OR Guadeloupe OR Guatemala OR Guyana OR Haiti OR Honduras OR Jamaica OR Martinique OR Mexico OR Mont Serrat OR “Netherlands Antilles” OR Nicaragua OR Panama OR Paraguay OR Peru OR “Puerto Rico” OR “Saint Barthelemy” OR “Saint Kitts and Nevis” OR “Saint Lucia” OR “Saint‐Martin” OR “Saint Vincent and the Grenadines” OR “Sint Maarten” OR Suriname OR “Trinidad and Tobago” OR “Turks and Caicos” OR Uruguay OR “Virgin Islands” OR Venezuela

Got 38,312 results, and added parentheses in the search box as follows:

(kw:Read* OR Literacy) AND (kw:”primary school” OR “primary grade” OR “grades 1 through 3” OR “grades 1 to 3” OR “grades 1–3” OR “first through third” OR “Grade 1” OR “first grade” OR “grade 2” OR “second grade” OR “grade 3” OR “third grade” OR “early grade” OR elementary OR kindergarten OR “pre‐school” OR “preschool” OR “pre‐kindergarten” OR “prekindergarten” OR preK OR “pre‐K” OR “early childhood”) AND (kw:”Latin America” OR Caribbean OR “South America” OR “Antigua and Barbuda” OR Argentina OR Aruba OR Bahamas OR Barbados OR Belize OR Bermuda OR Bolivia OR Brazil OR “British Virgin Islands” OR “Cayman Islands” OR Chile OR Colombia OR “Costa Rica” OR Cuba OR Curacao OR Dominica OR “Dominican Republic” OR Ecuador OR “El Salvador” OR “French Guiana” OR Grenada OR Guadeloupe OR Guatemala OR Guyana OR Haiti OR Honduras OR Jamaica OR Martinique OR Mexico OR Mont Serrat OR “Netherlands Antilles” OR Nicaragua OR Panama OR Paraguay OR Peru OR “Puerto Rico” OR “Saint Barthelemy” OR “Saint Kitts and Nevis” OR “Saint Lucia” OR “Saint‐Martin' OR “Saint Vincent and the Grenadines” OR “Sint Maarten” OR Suriname OR “Trinidad and Tobago” OR “Turks and Caicos” OR Uruguay OR “Virgin Islands” OR Venezuela) AND yr:1990..2016

Hit search again, and got 102 results, relevant.

## APPENDIX B DATA EXTRACTION FORM

**Table:**

Effect Size Extraction/Coding
Study ID (sid):
Need to Contact Authors (authors):
Coders initials (coderid):
Date coded (date):
Country (cntry):
Region in the world (region):
Intervention type (inter):
Grade at start of the intervention (grade_st):
Grade at time of impact estimate (grade_imp):
Age of children at the start of the intervention (age_st):
Age of children at time of impact estimate (grade_imp):
Methodology (method):
Outcome measure (outcat): (1) reading comprehension (2) letter naming (3) letter sounds (4) time spent on reading (5) vocabulary (6)phoneme segmentation (7) letter‐naming fluency (8) word reading (9) new word learning (10) fluency reading time together (11) comprehension (12) literacy scores (13) reading (14) spelling (15) English (16) letter word identification (17) early writing (18) language
Outcome name (outname):
With covariates (_covar):
Effect size type (estype): (1) Standardized mean difference (2) other
Other name (oth_name):
Direction of effect (esdir): (1) effect favors treatment (2) effect favors comparison (3) effect favors neither (4) cannot tell
Effect is statistically significant (essig)?: (1) yes (2) no (3) cannot tell
Treatment students sample size (trtss):
Comparison students sample size (compss):
Total students sample size (totals):
Treatment cluster sample size (trtss_clus):
Comparison cluster sample size (compss_clus):
Total cluster sample size (totals_clus):
*For continuous measures:*
Treatment group mean (txmean):
Comparison group mean (compmean):
Are means reported above adjusted? (meanadj): (1) yes (2) no
Treatment group standard deviation (txsd):
Comparison group standard deviation (compsd):
Treatment group standard error (txse):
Comparison group standard error (compse):
Mean difference (mdiff):
Standard error mean difference (semdiff):
Standard error in regression (seregress):
Standard error in matching (sematching):
*t*‐value regression or single difference (est)
Pooled standard deviation (psd):
Standardized mean difference (smd):
Small sample size adjusted standardized mean difference (ssmd):
Standard error Standardized mean difference (se_smd):
*t*‐value standardized mean difference (est_smd)
Treatment time (trt_time):
Source: Wilson et al. (2014)size bias by relying on Equation

## APPENDIX C DESCRIPTION OF THE INTERVENTIONS

**Table C1:** Summary of quantitative intervention studies

Study title	Authors (year)	Location researcher	Implementer	Context	Outcomes	Sample	Study design	Intervention/program	Analysis
Lectura compartida de cuentos y aprendizaje de vocabulario en edad preescolar: un estudio de eficacia	Larraín et al. (2012), experiment 1	Chile	Academic	Santiago, Chile	Vocabulary acquisition	112 children from three public kindergartens	RCT	Shared book reading without word elaboration	*T* test
Lectura compartida de cuentos y aprendizaje de vocabulario en edad preescolar: un estudio de eficacia	Larraín et al. (2012), experiment 2	Chile	Academic	Santiago, Chile	Vocabulary acquisition	62 children from three public kindergartens	RCT	Shared book reading with word elaboration	*T* test
Desarollo de Habilidades Conductuales Maternas Para Promover la Alfabetacion Inicial en Niños Prescolares	Tapia and Benítez (2013)	Mexico	Academic	Mexico	Literacy practices in mothers	20 women with limited literacy practices whose preschool children showed low levels in preacademic and linguistic skills	RCT	Training for mothers to conduct activities and strategies to promote language and preacademic skills related to early literacy in preschool children using joint reading of stories and puppet play	ANOVA
The effects of early childhood education on literacy scores using data from a new Brazilian assessment tool	Felício et al. (2012)	Brazil	Ministry of Education	Brazil	Literacy scores	1,986 second grade students	Quasiexperimental	Enrollment in preschool	Propensity score matching
Exploring teachers' read‐aloud practices TRAP as predictors of children's language skills: The case CLSTC of low income chilean preschool classrooms	Gomez Franco (2014)	Chile	Ministry of Education	Chile	Teachers' speech characteristics	913 students across 47 preschools	RCT	Teacher training program for teachers in preschool	ANOVA
					Teachers' read‐aloud strategies				
The impact of improving nutrition during early childhood on education among guatemalan adults	Maluccio et al. (2009)	United State	Institute of Nutrition of Central America and Panama	Guatemala	Grades completed	1,471 adults from one of four villages	RCT	Provision of nutrient dense drink to children	OLS regression analysis
					Reading comprehension				
					Nonverbal cognitive				
Parents as reading tutors for first graders in Brazil	Murad & Topping (2000)	United State	Academic	Brazil	Reading comprehension	48 students from a single school	RCT	Training for parents to participate in paired reading with their children	Bivariate comparison
					Reading fluency				
The effect of a multicomponent literacy instruction model (MLIM) on literacy growth (LG) for kindergartners (K) and first‐grade students (FGS) in Chile	Pallante & Kim (2013)	Chile	Collaborative Language and Literacy Instruction Project	Chile	Letter naming	312 kindergartners; 305 first graders	Cluster RCT	Teacher training during five workshops for teachers complemented with teaching resources	Difference‐in‐difference regression analysis
					Word reading				
					Vocabulary				
					Phonemic segmentation fluency				
Letter names and phonological awareness help children to learn letter—Sound relations	Cardoso‐Martins et al. (2011), experiment 1	Brazil	Academic	Brazil	Letter sound learning	32 children/20 children	RCT	Phonological training for children about the shapes and names of letters	ANOVA
					Decoding				
Letter names and phonological awareness help children to learn letter–Sound relations	Cardoso‐Martins et al. (2011), experiment 2	Brazil	Academic	Brazil	Letter sound learning	32 children/20 children	RCT	Phonological training for children about the shapes and names of letters with extra emphasis on beginning and middle sound letters	ANOVA
					Decoding				
Treatment of *Trichuris trichiura* infections improves growth, spelling scores and school attendance in some children	Simeon et al. (1995)	United States	Academic	Jamaica	Spelling Reading	407 children	RCT	Deworming program	OLS regression analysis
Do in‐school feeding programs have impact on academic performance and dropouts? The case of public Argentine schools	Adrogue & Orlicki (2013)	United States	Ministry of Education	Argentina	Language test score	4,466 schools	Difference‐in‐differenced	School feeding program	OLS regression analysis
Technology and child development: evidence from the one laptop per child program	Cristia et al. (2012)	Peru	Ministry of Education	Peru	Language test scores	4,111 students	RCT	Provision of one laptop per child	OLS regression analysis
Nutrition and education: A randomized trial EART of the effects of breakfast in rural primary school children	Powell et al. (1998)	United States	Academic	Jamaica	Spelling Reading	814 students	RCT	Provision of nutritious breakfast by schools	OLS
Learning with the XOs: The impact of the Ceibal plan	Ferrando et al. (2011)	Uruguay	Ministry of Education	Uruguay	Reading	1,365 students	Difference‐in‐differenced	Provision of one laptop per child	Propensity score matching
Guyana's hinterland community‐based school feeding programme	Ismail et al. (2014)	United State	Ministry of Education	Guyana	English language test scores	1,038 treatment and 1,938 control students	Quasiexperimental	Community‐based school feeding program	Propensity score matching
					Reading test scores				
The contribution of quality early childhood education and its impacts on the beginning of fundamental education	Campos et al. (2011)	Brazil	Ministry of Education	Brazil	National literacy exam test scores	605 treatment and 157 comparison students	Nonexperimental	Preschool	Hierarchical regression analysis
School improvement plans and student learning in Jamaica	Lockheed et al. (2006)	United States	Ministry of Education	Jamaica	National literacy and language school‐level performance data	71 treatment and 67 comparison schools	Quasiexperimental	The program provided support to schools on the basis of needs identified through the preparation of a School Development Plan	Propensity score matching
Opening the black box: Intervention fidelity in a randomized trial of a preschool teacher professional development program	Mendive et al. (2016)	Chile	Ministry of Education	Chile	Child language and literacy	1,033 treatment students and 843 control students	RCT	Teacher training program	OLS regression analysis
Effects of story reading on language	Vivas (1996), experiment 1	Venezuela	Academia	Venezuela	Language comprehension and expressive language	72 treatment and 84 control students	RCT	Systematic, story‐reading‐aloud program at school	ANOVA
Effects of story reading on language	Vivas (1996), experiment 2	Venezuela	Academia	Venezuela	Language comprehension and expressive language	72 treatment students and 66 control students	RCT	Systematic, story‐reading‐aloud program at home	ANOVA
The use and misuse of computers in education: Evidence from a randomized experiment in Colombia	Barrera‐Osorio and Linden (2009)	United States	Ministry of Communication	Colombia	Language test scores	4,327 treatment students and 3,889 control students	RCT	Provision of computers for instruction	OLS regression analysis
Experimental impacts of a teacher professional development program in Chile on preschool classroom quality and child outcomes	Yoshikawa et al. (2015)	United States	Ministry of Education	Chile	Child language and literacy	1,033 treatment students and 843 control students	RCT	Teacher training program	OLS regression analysis
Home computers and child outcomes: Short‐term impacts from a randomized experiment in Peru	Beuermann et al. (2015)	United States	Ministry of Education	Peru	Time spent reading	Total sample of 2,817	Difference‐in‐differenced	Provision of educational laptop	OLS
					Language Test Scores				
Read‐Alouds in Calca, Peru: A bilingual indigenous context	Neugebauer and Currie‐Rubin (2009)	United States	Academia	Peru	Vocabulary acquisition	29 treatment students and 26 control students in two treatment classrooms and two control classrooms	RCT	Read‐aloud program	OLS
					Reading comprehension				
The effect of school based management on parent behavior and the quality of education in Mexico	Bando (2010)	United States	Ministry of Education	Mexico	Parent donations to school	250 *telesecundarias*, or secondary schools that rely on television instruction	RCT	School based management—transfer funds directly to school and parents	OLS regression analysis
					Education quality				

Abbreviations: ANOVA, analysis of variance; OLS, ordinary least squares; RCT, randomized controlled trial.

**Table C2:** Summary of quantitative nonintervention studies

Study title	Authors (year)	Location researcher	Context	Outcomes	Sample	Study design	Analysis
Three instruments for assessing the abilities of learning to read and write	Oliveira (1996)	Brazil	Araraquara, Brazil	Reading and writing abilities	140 first graders	Diagnostic Test of Preschool Skills; Readiness Test for Literacy; Instrument for Assessing the Basic Repertoire for Literacy	Kuder‐Richardson test
La Memoria Visual Como Predictor del Aprendizaje de la Lectura	Pino & Bravo (2005)	Chile	Santiago, Chile	Visual perception, visual memory, and visual‐orthographic recognition of words as predictors of early grade reading	105 first graders	ELEA Evaluation Instrument; Initial Literacy Test; King's Complex Figure; Interamerican Literacy Test	Factorial analysis, regression
A computer system for reading assessment in Spanish‐speaking school‐age children	Reigosa‐Crespo et al. (1994)	Havana, Cuba	Cuba	Phonological awareness and Word reading	120 children ages 6–11	Automated system that uses videogames to assess rhymes, alliteration, quickly naming figures, and reading regular, irregular, and pseudowords	ANOVA, correlational, factorial regressions
Traditional Assessment or Invisible Assessment Using Games? New Frontiers in Cognitive Assessment	Rosas et al. (2015)	Chile	Santiago, Chile	Phonological awareness	337 children in kindergarten through grade 3	Quasiexperimental pilot of a so‐called “invisible evaluation” and traditional tests	Mixed‐design analysis of variance
Tests used to predict reading acquisition in Guatemala city: Predictive validity and new analysis of the ABC	Salazar et al. (1996)	Guatemala	Guatemala City, Guatemala	Validity of reading predictor tests	185 beginning readers	ABC reading ability test	Correlational, simple, and multiple regression
Early narrative skills in Chilean preschool: Questions scaffold the production of coherent narratives	Silva et al. (2014)	Chile and United Kingdom	Chile	Narrative construction	60 kindergarteners	Students asked to talk about pictures in different contexts	*T* test
Assessment and evaluation of phonological awareness, concepts of print, and early reading and writing in young chilean children: A comparison with international results	Villalon and San Francisco (2001)	Chile and United States	Santiago, Chile	Phonological awareness, print concepts, and reading and writing	58 kindergarteners 57 first graders	Test of Early Literacy	Correlational analysis
Habilidades de lectura en primer grado en alumnos de estrato sociocultural bajo	Guevara et al. (2008)	Mexico	Mexico	Reading skills	165 preschool students	Precurrent Skills for Reading Assessment (PSRA)	ANOVA
Metalinguistic awareness and literacy: A study carried out among first elementary grade children	Barrera & Maluf (2003)	Brazil	Sao Paulo, Brazil	Phonological, lexical, and syntactic awareness	65 first grade students	Researcher‐adapted tests for phonological awareness; lexical awareness; syntactic awareness; reading and writing	Spearman correlations and Wilcoxon test
Predictors of reading and writing achievement in first graders	Corral Verdugo et al. (2000)	Mexico	Sonora, Mexico	Reading and writing	288 first grade students	76 items based on morphological and functional criterion	Multiple regression
Phonological awareness and emergent reading in children who enter first elementary grade	Bravo et al. (2002)	Chile	Santiago, Chile	Phonological awareness	399 first grade students	Predictive Processes for Literacy; Initial Literacy Test	Multiple regression
Cognitive skills in children: Comparing good readers and poor readers	Capovilla et al. (2004)	Brazil	Sao Paulo, Brazil	Cognitive skills	90 preschoolers and first graders	Binary classification of silent reading test (TeCoLeSi); other reading subskills such as manipulating spoken word sounds; vocabulary test; international dyslexia test	ANCOVA; Raven Matrices test
Cognitive skills that predict competencies in reading and spelling.	Capovilla et al. (2004)	Brazil	Sao Paulo, Brazil	Reading and writing competencies	54 kindergarten and first graders	Binary classification of silent reading test (TeCoLeSi); phonological awareness tests; Peabody pictures tests; vocabulary from figures; international dyslexia test	Regression analysis
The development of spelling skills in the preschool years: Questions about the syllabic stage	Cardoso‐Martins and Fulanete Correa (2008)	Brazil	Brazil	Spelling development	20 preschool children	Researcher‐developed tests	Correlation across two halves
Oral language and reading abilities of first‐grade Peruvian children: Associations with child and family factors	Castro et al. (2002)	United States	Lima, Peru	Picture vocabulary, verbal analogies, letter‐word identification, and reading comprehension	137 first graders	Parent interviews; Picture Vocabulary and Verbal Analogies; Letter‐Word Identification; and Reading Comprehension	ANOVA, multivariate multiple regression
Trabajo infantil y logro escolar en América Latina ‐los datos del SERCE	Cervini (2015)	Argentina	Multiple Countries	Student performance in math and reading	83,159 primary school students in 16 countries and Mexican state of Nuevo León	Segundo Estudio Regional Comparativo y	Bivariate multilevel models
						Explicativo (SERCE) data	
Unconventional word segmentation in Brazilian children's early text production	Correa and Dockrell (2007)	United Kingdom and Brazil	Rio de Janeiro, Brazil	Awareness of morphemes, reading accuracy tests, nonverbal ability, verbal ability, vocabulary, working memory	76 children	A series of tasks; Weschsler Intelligence Scale for Children‐III	Kolmogorov‐Smirov test
					Years 1–3		
The impact of preschool programs in the achievement of first graders in elementary public schools of Lima	Cueto and Díaz (2013)	Peru	Lima, Peru	Preschool programs' impact on student achievement	304 students in public schools who were first graders in 1997	Surveys on schools and students	Probit regression
Alphabetic access route in beginning reading acquisition in Portuguese: The role of letter‐name knowledge	De Abreu and Cardoso‐Martins (1998)	Brazil	Brazil	Reading acquisition	48 preschoolers	Researcher developed tests on reading words, alphabet knowledge, phonological awareness, and word‐learning tasks	ANOVA
Phonological awareness, spelling and reading abilities in Spanish‐speaking children	de Manrique & Signorini (1994)	Argentina	Buenos Aires, Argentina	Reading and spelling	64 first graders	Researcher developed tests on phoneme segmentation; spelling; and word reading	T test
Cognitive processes involved in children's word reading: Relations with reading comprehension and reading time	Salles and Parente (2002)	Brazil	Brazil	Reading comprehension	76 third graders	Researcher developed tests for reading isolated words; reading comprehension; and reading speed	Cluster analysis, ANOVA
Achievement performance assessment of elementary school students in Vitória, Espírito Santo, Brazil	Dias et al. (2006)	Brazil	Brazil	Academic performance in reading, writing, and arithmetic	172 students in grades 2–5	School Achievement Test	ANOVA
Phonological awareness and phonological memory tasks are predictor factors of reading and writing abilities	Favila et al. (1999)	Mexico	Mexico	Phonological awareness, reading and writing	29 first graders	Articulation inventory; phonological processing test; test to analyze reading and writing	Linear regression; multiple regression
Applications of cloze procedure to reading assessment in special circumstances of literacy development	Francis (1999)	United States	Mexico	Effectiveness of the cloze test as a means of assessing reading in an indigenous language	Third and fifth grade bilingual students	Cloze test	Percentage score based on a scale rating
How important is teaching phonemic awareness to children learning to read in Spanish?	Goldenberg et al. (2014)	United States and Mexico	United States and Mexico	Phonemic awareness	189 Mexican students	Test of Phonological Processes; Woodcock Language Proficiency Battery‐Revised; Classroom observations	Regression analyses and ANOVA
					280 U.S. students receiving Spanish instruction		
					102 U.S. students receiving English instruction		
					Grades 2–5		
Evaluación de los procesos cognitivos de la lectura a través del SICOLE‐R‐Primaria en niños que cursan la educación primaria: un estudio transversal	Gómez‐Pérez et al. (2011)	Mexico	Guadalajara, Mexico	IQ, lexical processing, syntactic processing	685 students from grades 2–6	SICOLE‐R software	ANOVA, MANOVA
The reality of the acquisition of oral and written language in a sample of schools in the southern part of Santiago	Oyarce and Mujica (2001)	Chile	Santiago, Chile	Reading and writing	111 first graders	Reading Comprehension Test of Progressive Linguistic Complexity	Analysis not described
Dual language and literacy development of Spanish‐speaking preschool children	Páez et al. (2007)	United States	Massachusetts, Maryland, and Puerto Rico	Oral language and early literacy	319 bilingual children in Massachusetts and Maryland and 144 monolingual children in Puerto Rico in preschool	Woodcock Language Proficiency Battery and researcher‐developed phonological awareness tasks	ANOVA
How to raise children's early literacy? The influence of family, teacher, and classroom in northeast Brazil	Fuller et al. (1999)	Brazil	Brazil	Early literacy skills	94 schools, 140 teachers, and 1,925 children	Early Literacy Exam	MANOVA
Evidências de validade do subteste de leitura do teste de desempenho escolar	Athayde et al. (2014)	Brazil	Brazil	Reading subtest accuracy	1,831 students from grades 1–6	TDE	Factor analysis; ANOVA
Teste do Desempenho Escolar: evidências de validade do subteste de escrita	Giacomoni et al. (2015)	Brazil	Brazil	Writing subtest accuracy	1,831 students from grades 1–6	TDE	Factor analysis; ANOVA
Measuring metacognitive strategies using the reading awareness scale ESCOLA	Jiménez et al. (2009)	Spain	Spain and Argentina	Metacognition	375 Spanish and 309 Argentine students aged 8–13	Reading comprehension tests; Metacognitive Awareness of Reading Strategies Inventory (MARSI) ESCOLA (Reading Awareness Scale)	Cronbach's *α*
Habilidades prelectoras de estudiantes de preescolar en la región caribe colombiana	Silva et al. (2013)	Colombia, United States	Colombia	Prereading skills	350 preschool students	Nepsy II test; auditory listening scale	Descriptive statistics
The emergence of sentence reading as stemming from teaching their constituent parts	Medeiros et al. (2011)	Brazil	Brazil	Emergence of reading sentences	17 children ages 6–8	A computer program that tested children on responses to the relationship between words, numerals, and color names	Percentage counts
Reading acquisition through an errorless discrimination procedure (exclusion): A replication with preschoolers	Melchiori et al. (2012)	Brazil	Brazil	Reading acquisition	Three preschoolers	Match written words to dictated words	Percentage scores based on correct answers
Relationship Between Emergent Literacy Skills and Reading, From Kindergarten to First Grade	Guardia (2003)	Chile	Chile	Phonological awareness, knowledge of letters, rapid naming and reading	100 children in kindergarten, then in first grade	Subtests of phonological awareness	Factorial analysis
Adult consequences of growth failure in early childhood	Hoddinott et al. (2013)	Multiple	Guatemala	Adult human capital, marriage, fertility, health, and economic outcomes	1,338 Guatemalan adults (aged 25–42) who were studied as children in 1969–1977	Longitudinal study	Instrumental variable regression
Elementary school students as authors of a description: Stages in the learning of writing and linguistic‐discursive styles	Iparraguirre (2014)	Argentina	Patagonia, Argentina	Writing descriptive text	32 third graders and 31 seventh graders	Students asked to develop a handwritten description of a familiar topic: where they live	Correspondence Analysis and Ascendant Hierarchical Cluster Analysis
The role of morphology in reading acquisition in Spanish	Jaichenco and Wilson (2013)	Argentina and Canada	Buenos Aires, Argentina	Reading acquisition	Three groups of 20 children in grades 2–4	Researcher developed lexical decision test	ANOVA, Tukey test
Impact of impairment on children with special needs at school entry: Comparison of school readiness outcomes in Canada, Australia, and Mexico	Janus (2011)	Canada	Canada, Australia, Mexico	Kindergarten‐level school readiness	Kindergarten‐level special needs students: 183,710 children in Canada	Early Development Instrument	Descriptive statistics
					1,582 children in Australia		
					168,400 children in Mexico		
Frequency analyses of prephonological spellings as predictors of success in conventional spelling	Kessler et al. (2013)	Brazil and United States	Belo Horizonte, Brazil	Spelling development	76 children, first year of preschool	Spelling tasks; Wechsler Intelligence Scale for Children‐III	Kendall correlation test
Predictors of reading skills for kindergartners and first grade students in Spanish: A longitudinal study	Kim and Pallante (2012)	United States	Chile	Reading comprehension skills	305 kindergarten and first grade students	Longitudinal study; Woodcock Language Proficiency Battery; researcher adapted test items	Multilevel models
Measuring beginner reading skills: An empirical evaluation of alternative instruments and their potential use for policymaking and accountability in Peru	Kudo and Bazan (2009)	Peru	Junin, Peru	Reading performance	475 third graders in Peru	Oral fluency test; Ministry of Education's written comprehension test	Multiple regressions
Evaluación de la conciencia fonológica en párvulos de nivel transición 2y escolares de primer año básico, pertenecientes a escuelas de sectores vulnerables de la provincia de Concepción, Chile	Bizama et al. (2011)	Chile	Concepción, Chile	Phonological awareness	43 preschool and 42 first grade students	Linguistic Segmentation Test	*T* test, Wilcoxon test
Comparing cognitive performance in illiterate and literate children	Matute et al. (2012)	Mexico, United States	Guadalajara and Tijuana, Mexico	School‐related cognitive abilities	21 illiterate and 22 literate Mexican children aged 6–13	Soft Neurological Signs Evaluation	ANOVA
Habilidades psicolingüístieas al ingreso y egreso del jardín de niños	Moneda et al. (2009)	Mexico	Mexico	Reading and writing abilities	60 first and third graders	Illinois Test of Psycholinguistic Abilities	Percentage scores
Learning to read and phonological awareness: A psycholinguistic approach to early reading instruction	Muñoz (2002)	Chile	Macul, Chile	Phonological awareness	116 children in 1st year of primary school	Researcher developed phonological awareness and literacy tasks	*T* test
Monitoring basic skills acquisition through rapid learning assessments: A case study from Peru	Abadzi et al. (2005)	Multiple	Peru	Reading speed and comprehension	245 first and second graders	Children read a modified passage from a grade 1 textbook	Percentages and reading speed at words per minute
Literacy abilities of preschool deaf children remergent literacy	Bandini et al. (2006)	Brazil	Brazil	Emergent literacy	Seven bilateral deaf children, aged 6 and 7	Emergent Literacy Scale	Histograms
Linguistic skills of children from a low socioeconomic level at the beginning of elementary education	Guevara et al. (2007)	Mexico	Mexico	Linguistic skills	262 first grade students	Academic Performance Inventory test (IDEA)	ANOVA
Cognitive and language correlates of hyperlexia: Evidence from children with autism spectrum disorders	Cardoso‐Martins, and Da Silva (2010)	Brazil	Brazil	Vocabulary, nonverbal intelligence, and reading and spelling ability	Study 1: 18 school age children	Researcher developed tests; Peabody Picture Vocabulary Test; Weschsler Intelligence Scale for Children	Kruskal‐Wallis and Mann‐Whitney tests
					Study 2: 23 children		
Habilidades y conocimientos constitutivos de la alfabetización temprana: semejanzas y diferencias según el entorno social y las oportunidades educativas	Plana and Fumagalli (2013)	Argentina	Buenos Aires, Argentina	Literacy pattern of learning	56 kindergarten students in three groups	11 tests to assess literacy patterns of learning; some researcher developed and others borrowed from other studies	Variance analysis, Bonferroni posthoc test
La separación entre palabras en la escritura de niños que inician la escolaridad primaria	Querejeta et al. (2013)	Argentina	Mexico and Argentina	Reading, writing, and vocabulary tests	30 Argentinian and Mexican children	Spontaneous writing and dictated sentences and texts; Reading and Writing in Spanish; Wechsler Intelligence Scale for Children‐III	Descriptive statistics, Mann‐Whitney *U* Test
The connection between syntactic awareness and reading: Evidence from Portuguese‐speaking children taught by a phonic method	Rego (1997)	Brazil	Brazil	Reading acquisition	48 children aged 5–7	Researcher adapted tests to measure reading ability; IQ tests	Multiple regression
Numerical capacities as domain‐specific predictors beyond early mathematics learning: A longitudinal study	Reigosa‐Crespo et al. (2013)	Havana, Cuba	Havana City, Cuba	Decoding fluency, reading comprehension	49 children in grades 3 and 4	Ravens Colored Progressive Matrices; Basic numerical battery	*T* test; ANOVA
Phonological and lexical processing in normal primary school readers.	Reynoso‐Alcántara et al. (2010)	Mexico	Mexico City, Mexico	Phonological and lexical processing	194 children aged 7–13 in grades 2, 4, and 6	Researcher developed tests with tasks related to rhyming and nonrhyming words and pseudowords	ANOVA, Tukey test
Cognitive ability of preschool, primary and secondary school children in Costa Rica	Rindermann et al. (2014)	Germany, Austria, and Dominica	Pérez Zeledón, Costa Rica	Cognitive ability measurements	80 preschoolers	Cognitive ability measures for mental speed, fluid intelligence, and crystallized intelligence	Weighted averages and g‐factors
					73 fourth graders		
					60 secondary school students in Costa Rica		
			Styria, Austria				
					51 preschoolers		
					71 fourth graders		
					50 secondary schoolers in Austria		
Relaciones diferenciales entre experiencias de alfabetización y habilidades de alfabetización emergente	Salazar‐Reyes and Vega‐Pérez (2013)	Mexico	Mexico	Receptive and expressive vocabulary, phonological awareness, printed text concepts	34 families with children in 1st year of preschool	Oral language test of Navarra; Printed text concepts; questionnaire on parents' experiences with children at home	Descriptive statistics
Learning about the letter name subset of the vocabulary: Evidence from United States and Brazilian preschoolers	Treiman et al. (2006)	United States	Detroit, United States and Belo Horizonte, Brazil	Identify alphabet letters by name	318 United States and 369 Brazilian preschoolers	Children asked to name uppercase letters	ANOVA
Differential item functioning and educational risk factors in Guatemalan reading assessment	Morales (2013)	Guatemala, Netherlands, and South Africa	Guatemala	Differential Item Functions	20,553 third grade students' test results	Spanish national reading tests	Chi square, Rasch, and logistic regression
Consecuencias del trabajo infantil en el desempeño escolar	Torrecilla and Carrasco (2014)	Spain and Chile	Latin America	Student academic performance	Third and sixth grade students in Latin America	Segundo Estudio Regional Comparativo y	Multilevel models
						Explicativo (SERCE) data	

Abbreviations: ANCOVA, analysis of covariance; ANOVA, analysis of variance; MANOVA, multivariate analysis of variance; TDE, test of student performance.

**Table C3:** Summary of qualitative intervention studies

Study title	Authors (year)	Location researcher	Context	Outcomes	Sample	Study design	Intervention/program	Analysis
Read‐alouds in Calca, Peru: A bilingual indigenous context	Neugebauer and Currie‐Rubin (2009)	United States	Calca, Peru	Children's literacy acquisition	55 first graders in two intervention and two control classrooms	Pretest posttest mixed methods; classroom observations	Two intervention classrooms received instruction from teachers trained in read‐aloud approaches, while the other two were “business as usual”	Ordinary least squares; classroom observations
Teacher in transition: A case study of the change process from skills‐based to whole language teaching. Research in progress	Mahurt (1993)	United States	U.S. Virgin Islands	Pedagogical practices	A teacher who was interested in changing their pedagogical approach	Case study; teacher kept a log and a reflective journal; researcher conducted interviews and classroom observations	Observing a teacher change their teaching methodology from a traditional approach to a new approach	Thematic analysis: identified themes and phrases that support the themes
One size fits all: Perceptions of the revised primary curriculum at grades one to three in Jamaica	Roofe (2014)	United States	Jamaica	Student learning outcomes	11 teachers who teach grades 1–3	Key informant interviews and focus group discussions	Revised primary curriculum, designed to help students make connections between subject area and society to achieve quality learning outcomes	Thematic analysis: transcribed and coded data, identified themes
Redes de colaboración en situaciones de alfabetización familiar con niños pequeños. Un estudio en poblaciones urbano marginadas de argentina	Stein and Rosemberg (2012)	Argentina	Argentina	Early literacy acquisition	30 children in their home context	Conducted an average of 6.5 observations in each home; a total of 124 recordings of children's literacy interactions with parents	Hosted meetings with families in 11 community kindergartens or schools on topics related to linguistic development; provided a book series to families	Used the “Constant Comparative Method” to generate categories that help analyze the interactions
The role of collaborative work in the development of elementary students' writing skills	González et al. (2013)	Colombia	Ibagué, Colombia	Writing skills	536 students in a private bilingual school	Action research—observe, collect and analyze data; act on evidence and implement strategies	Stage 1: teachers collected information about most common characteristics students have when writing English	Thematic analysis: analyzed findings into four categories of observation
							Stage 2: teachers selected three strategies that they believed helped improve students' writing	
Exemplary teaching in the Caribbean: Experiences from early literacy classrooms	Warrican et al. (2008)	Barbados and Jamaica	Multiple Caribbean countries	Teachers' ability to teach literacy	21 teachers who teach grades 1–3	Teachers asked to write about their experiences	Teacher trainer provides training for reading specialists, who then train classroom teachers and monitor program implementation at school and classroom levels. Teachers also encouraged to use action research to identify problems and corresponding solutions	Researchers identified trends and patterns of behavior

**Table C4:** Summary of qualitative nonintervention studies

Study title	Authors (year)	Location researcher	Context	Outcomes	Sample	Study design	Analysis
La comprensión de cuentos como resolución de problemas en niños de 5 años de sectores urbano‐marginales	Manrique and Borzone (2010)	Argentina	Buenos Aires, Argentina	Text comprehension	Kindergarten students in nine classrooms	Observation in nine classrooms over 3 days; collected data through audio and video clips	Comparative constant method: analyze teacher–student interactions in 26 story‐reading settings
Latino media and critical literacy pedagogies: Children's scripting of Telenovelas discourses	Medina and Costa (2013)	United States (including Puerto Rico)	Puerto Rico	Critical literacy	A third grade classroom in Puerto Rico	Ethnographic research: participant observation and collecting student artifacts	Used analytical frameworks to analyze data
La práctica docente y el fomento de la lectura en colima	Gómez Nashiki (2008)	Mexico	Colima, Mexico	Teaching methods	161 students, 72 teachers, three principals, and 52 parents	Ethnographic research: interviews, observations, field notes	Thematic analysis: teacher identify, context, teacher practices, classroom, strategies, and promoting literacy
Efeitos do(s) letramento(s) na constituição social do sujeito: considerações fonoaudiológicas	Ribeiro and Souza (2012)	Brazil	Sao Paulo, Brazil	To improve teaching and speech therapy	69 third grade students	Researcher‐developed protocols to determine children's ability to recognize written genres, interviews	Mapped students' literacy rates depending on levels and types of literacy reflected in literature
Educação escolar: a vez e a voz das crianças	Rosado and Campelo (2011)	Brazil	Brazil	Children's participation in pedagogical approaches	20 elementary school children	Interviews, questionnaires, observations	lIlustrative examples
Reinventing texts and contexts: Syncretic literacy events in young Puerto Rican children's homes	Volk & De Acosta	United States	Puerto Rico	Literacy practices	Three kindergarteners	Ethnographic research: taped conversations, field notes, written texts	Thematic analysis: Identified and analyzed underlying themes in transcribed materials
Exploring the literature of fact	Webster (2009)	Jamaica	Jamaica	Students' academic performance in science	30 first grade students	Ethnographic research: field notes, interviews, students' artifacts	Thematic analysis: four themes identified and used to guide the paper
‘Many differing ladders, many ways to climb…’: Literacy events in the bilingual classroom, homes, and community of three Puerto Rican kindergartners	Volk and de Acosta (2001)	United States	Puerto Rico	Literacy development	Three kindergarteners	Ethnographic research: participant observation, interviews, and audio recordings	Data was analyzed as literacy events, which were then looked at in their larger context and analyzed using codes
Teaching English to very young learners through authentic communicative performances	Guevara and Ordoñez (2012)	Colombia	Colombia	Pedagogical strategies (communicative aperformances)	Four teachers' classrooms	Teacher interviews, classroom observations, class recordings	Discourse analysis, triangulated findings from interviews and classroom observations
El Aprendizaje Inicial de la Lectura y la Escritura de Palabras en Español: Un Estudio de Caso	Diuk (2007)	Argentina	Buenos Aires, Argentina	Reading and spelling acquisition	Two girls, aged 5 and 6	Case study, conducted various tests on their phonological awareness	Compared and tracked girls' phonological awareness over time
Freedom and form: The language and literacy practices of two Mexican schools	Jiménez et al. (2003)	United States and Mexico	Mexico	Written and spoken language	Two primary schools (one with 400 students, the other with 100 students)	Qualitative study using ethnographic techniques: observations, interviews, attending meetings, reviewing extant data	Thematic analysis: Identified themes from observations of multiple teachers
Family, culture, literacy and the kindergarten classroom: Perspectives of an immigrant teacher	Kinkhead‐Clark (2014)	Jamaica	Jamaica	Children's literacy development	Three students	Case study: interviews, artifacts, and observations	Thematic analysis: analyzed data according to three themes
Las tareas de conciencia fonológica en preescolar	Leal Carretero & Suro Sánchez (2012)	Mexico	Multiple countries	Improved phonological awareness tests	21 phonological awareness tests for preschool children	Systematic review	Analyzed tests based on linguistic criteria
Deaf children's construction of writing	Massone and Baez (2009)	Argentina	Argentina	Literacy acquisition	15 deaf children, grades K‐3	Interviews children on nine cards that had an image and string of written words	Percentages of response types

## APPENDIX D RISK OF BIAS TOOLS AND RESULTS

**Table D1:** Quantitative intervention risk of bias assessment tool and risk of bias assessment for included quantitative intervention studies

Code description	Code	Comment
Study ID	Last name of author, year	Open answer
Justification of use	Study design and methodology	Open answer
*Ask these questions for all quantitative studies*		
Did the outcome measure include some measure of reading or a reading subskill (e.g., fluency, phonological awareness, language, decoding, letter knowledge, comprehensions etc.)?	Yes	Comment: open answer
	No	
	Unclear	
	Not applicable	
If the study did not include a measurement of reading or a reading subskill, is literacy measured in a different manner?		
Does the study show baseline reading/literacy abilities for beneficiaries and nonbeneficiaries?		
If reading/literacy scores are not available at baseline, does the study show characteristics of beneficiaries and nonbeneficiaries that are not likely to be affected by the intervention?		
Are the mean values or the distributions of the covariates at baseline statistically different for beneficiaries and nonbeneficiaries (*p* < .05)		
If there are statistically significant differences between beneficiaries and nonbeneficiaries are these differences controlled for using covariate analysis in the impact evaluation?		
If baseline characteristics are not available, does the study qualitatively assess why beneficiaries are likely/unlikely to be a random draw of the population at baseline?		
*Confounding and selection bias (ask questions for all quantitative studies)*		
Does the study use a comparison/control group of students/households without access to the program?	Yes	Comment: open answer
	No	
	Unclear	
	Not applicable	
Does the study use a comparison/control group of students/households with access to the program but that did not choose to participate in the program?		
Does the study include data at baseline and endline (before and after the intervention)?		
Are the data on covariates collected at the baseline?		
Is difference in differences estimation used?		
If the study is quasiexperimental and uses difference‐in‐difference estimation do the authors assess the parallel trends assumption?		
If the study does not use difference in difference, does the study control for baseline values of the outcome of interest		
If the study does not use difference in difference and does not control for baseline values of the outcome variable, does the study control for other covariates at baseline		
If the study does not use difference in differences estimation, is there any assessment of likely risk of bias from time invariant characteristics driving both participation and outcome?		
If the study does not use difference in difference estimation but does assess likely risk of bias from time invariant characteristics, are these time invariant characteristics likely to bias the impact estimates		
Does the study report the table with the results of the outcome equation (including covariates)? Where full results of the outcome equation are not reported, is it clear which covariates have been used?		
Are all relevant observable covariates (confounding variables) included in the outcome equation which might explain outcomes, if estimation does not use a statistical technique to control for selection bias (RCT, PSM, or covariate matching, IV or switching regression)? This might, for example, include control for ability, and/or social capital		
*Attrition (ask questions for all quantitative studies)*		
For studies including baseline data, does the study report attrition (drop‐out) from the study?	Yes	Comment: open answer
	No	
	Unclear	
	Not applicable	
Is the attrition rate below 10%?		
Does the study assess whether drop‐outs are random draws from the sample (e.g., by examining correlation with determinants of outcomes, in both treatment comparison group)?		
*Spillovers and contamination (ask questions for all quantitative studies)*		
Spillovers: are comparisons sufficiently isolated from the intervention (e.g., participants and nonparticipants are sufficiently geographically or socially separated) or are spillovers estimated by comparing nonbeneficiaries with access to the intervention to nonbeneficiaries without access to the intervention and/or through social network analysis?	Yes	Comment: open answer
	No	
	Unclear	
	Not applicable	
Spillovers; if spillovers are not estimated, is the study likely to bias the impact of the program?		
Contamination: does the study assess whether the control group receives the intervention?		
Contamination: if the control group receives the intervention but for a shorter amount of time does the study assess the likelihood that the control group has received equal benefits as the treatment group		
Contamination: if the control group receives the intervention have they received the intervention sufficiently long to argue that they have benefited from the intervention		
Contamination: does the study describe and control for other interventions which might explain changes in outcomes?		
*Other threats to validity (ask questions for all quantitative studies)*		
Does the evidence suggest analysis reporting biases are a serious concern? Analysis reporting biases include failure to report important treatment effects (possibly relating to intermediate outcomes), or justification for (uncommon) estimation methods, especially multivariate analysis for outcomes equations	1 = Yes	Comment: open answer
	2 = No	
	9 = Unclear	
	99 = Not applicable	
Are there concerns about baseline data collected retrospectively		
Are there concerns about courtesy bias from outcomes collected through self‐reporting?		
*Construct Validity (ask questions for all quantitative studies)*		
Were reading outcomes measured in the majority of the appropriate languages?	1 = Yes	Comment: open answer
	2 = No	
	9 = Unclear	
Does the study describe the implementation of the program in sufficient detail?		
	99 = Not applicable	
Was the unit of allocation and the unit of analysis the same?		
Do all students targeted by the study take the reading test/answer the survey questions?		
Does the study take into consideration potential implementation failures		
Does the study use a proper theory of change, logframe and/or other proper conceptual or theoretical framework?		
Does the study analyze the outcome measures put forward in the theory of change or logframe?		
*External Validity (ask questions for all quantitative studies)*		
Do the authors clearly distinguish between the intention‐to‐treat effect and the treatment effect on the treated?	1 = Yes	Comment: open answer
	2 = No	
	9 = Unclear	
	99 = Not applicable	
Do the authors highlight the intention‐to‐treat effect?		
*Hawthorne and John Hendry Effects (ask questions for all quantitative studies)*		
Do the authors argue convincingly that it is not likely that being monitored influences the behavior of the beneficiaries and nonbeneficiaries in different ways?	1 = Yes	Comment: open answer
	2 = No	
	9 = Unclear	
	99 = Not applicable	
*Confidence Intervals (ask questions for all quantitative studies)*		
Does the study account for lack of independence between observations within assignment clusters if the outcome variables are clustered?	1 = Yes	Comment: open answer
	2 = No	
	9 = Unclear	
Is the sample size likely to be sufficient to find significant effects of the intervention?	99 = Not applicable	
Do the authors control for heteroskedasticity and/or use robust standard errors?		
*Ask questions below only for studies that apply randomization*		
Does the study apply randomized assignment?	1 = Yes	Comment: open answer
	2 = No	
	9 = Unclear	
	99 = Not applicable	
Does the study use a unit of allocation with a sufficiently large sample size to ensure equivalence between the treatment and the control group?		
*Ask questions below only for studies that apply regression discontinuity designs*		
Is the allocation of the program based on a predetermined continuity on a continuous variable and blinded to the beneficiaries or if not blinded, individuals cannot reasonably affect the assignment variable in response to knowledge of the participation rule?	1 = Yes	Comment: open answer
	2 = No	
	9 = Unclear	
	99 = Not applicable	
Is the sample size immediately at both sides of the cut‐off point sufficiently large to equate groups on average?		
Is the mean of the covariates of individuals immediately at both sides of the cut‐off point statistically significantly different for beneficiaries and nonbeneficiaries?		
If there are statistically significant differences between beneficiaries and nonbeneficiaries are these differences controlled for using covariate analysis?		
*Ask questions below only for studies that apply matching*		
*Quality of matching (PSM, covariate matching)*		
Are beneficiaries and nonbeneficiaries matched on all relevant characteristics?	1 = Yes	Comment: open answer
	2 = No	
	9 = Unclear	
	99 = Not applicable	
Does the study report the results of the matching function (e.g., for PSM the logit function)?		
Does the study report the matching method?		
Does the study exclude observations outside the common support?		
Does the study use variables at follow‐up that can be affected by the intervention in the matching equation?		
Are matches found for the majority of participants (>90%)?		
If ≥10% of participants failed to be matched, is sensitivity analysis used to re‐estimate results using different matching methods?		
For nearest‐neighbor PSM, does the study report the mean or distribution of the propensity scores in the treatment and control groups after matching?		
For nearest‐neighbor PSM, are propensity scores similar, based on tests for statistical differences at the means or other quantiles of the distribution)?		
Does the study report the mean or distribution for the covariates of the treatment and control groups after matching?		
Are these characteristics similar, based on tests for statistically significant differences (*p* > .5)?		
*Sensitivity analysis (only for studies that apply PSM)*		
For PSM, where propensity score distributions and/or covariates of the treatment and control groups are not reported, or they are reported but there are differences in means or distributions of the covariates or propensity scores (usually only applicable to methods which do not exclude treatment observations such as nearest neighbor), is robustness assessed using an additional matching technique?	1 = Yes	Comment: open answer
	2 = No	
	9 = Unclear	
	99 = Not applicable	
Is sensitivity to hidden bias assessed statistically, for example, using the Rosenbaum bounds test?		
*Ask questions below only for studies that apply instrumental variable estimation*		
*Quality of IV, two‐steps endogenous switching regression approach*		
Does the study describe clearly the instrumental variable(s)/identifier used?	1 = Yes	Comment: open answer
	2 = No	
	9 = Unclear	
	99 = Not applicable	
Are the results of the participation equation reported?		
Are the instruments jointly significant at the level of F ≥ 10? If an *F* test is not reported, does the author report and assess whether the *R* ^2^ of the instrumenting equation is large enough for appropriate identification (*R* ^2^ > 0.5?)		
Are the instruments individually significant (*p* ≤ .05)?		
For IV, If more than one instrument is used in the procedure, does the study include and report an overidentifying test (*p *≤ .05 is required to reject the null hypothesis)?		
Does the study qualitatively assess the exogeneity of the instrument/identifier (both externality as well as why the variable should not enter by itself in the outcome equation)?		
*Ask questions below only for studies with censored outcome variables*		
Do the authors use appropriate methods (e.g., Heckman selection models, tobit models, duration models) to account for the censoring of the data?	1 = Yes	Comment: open answer
	2 = No	
	9 = Unclear	
	99 = Not applicable	
For Heckman models; is there is a variable that is statistically significant in the first stage of the selection equation and excluded from the second stage		
*Overall assessment*		
Assessment Selection Bias	Low risk of bias	Comment: open answer
	Medium risk of bias	
	High risk of bias	
	Unclear risk of bias	
Assessment Spillovers and Contamination Bias		
Assessment Outcome and Analysis Reporting Bias		
Assessment Other biases		

Abbreviation: RCT, randomized controlled trial.

**Table D2:** Risk of bias results for included quantitative intervention studies

	Selection bias and confounding	Performance bias: assessment, spillovers, and contamination	Outcome and analysis reporting biases	Other biases
Adrogue and Orlicki (2013)	*High risk of selection bias and confounding*	*Medium risk of performance Bias*	*High risk of outcome and analysis reporting bias*	*Low risk of other biases*
	This study uses a nonexperimental difference‐in‐difference design to determine the effect of the program on school‐level EGL outcomes. We rated the study as high risk of selection bias because the study considers outcome variables 4 months after the start of the study as baseline values. However, these values may well have been affected by the program at that time. In addition, it is unclear whether the comparison group was similar to the beneficiary schools at the time of the start of the intervention	The authors do not account for the possibility of crossover effects. Students in the comparison schools may have switched to treatment schools because of the school feeding program. This behavioral change may result in spillovers to the comparison group	The authors use an unusual difference‐in‐difference approach in which outcome measures after the start of the intervention are used as baseline values. This approach can result in considerable bias	There is no evidence for significant other risks of bias
Bando (2010)	*Medium risk of selection bias and confounding*	*Low risk of performance bias*	*Low risk of outcome and analysis reporting bias*	*Low risk of other biases*
	This study uses a regression analysis that includes school and state‐year fixed effects to determine the effect of a school governance program on EGL outcomes. Although this method does not fully account for the risk of selection bias, the risk of selection bias is only medium	The analysis compares beneficiary schools with comparison schools that appear to be sufficiently isolated from the beneficiary schools	There are no significant outcome and analysis reporting biases. The study uses a number of robustness checks to assess the validity of the results	There is no evidence for significant other risks of bias
Barrera‐Osorio and Linden (2009)	*Low risk of selection bias and confounding*	*Low risk of performance bias*	*Low risk of outcome and analysis reporting bias*	*Low risk of other biases*
	This study uses a cluster‐randomized controlled trial to determine the impact of the distribution of computers on EGL outcomes. Although attrition was high, the authors were able to credibly account for this in the analysis. Thus the risk of selection bias and confounding was low	The analysis compares beneficiary schools with comparison schools that appear to be sufficiently isolated from the beneficiary schools to prevent performance bias	There are no significant outcome and analysis reporting biases	There is no evidence for significant other risks of bias
Beuermann et al. (2015)	*Low risk of selection bias*	*Low risk of performance bias*	*Low risk of outcome and analysis reporting bias*	*Low risk of other biases*
	The study uses a cluster‐randomized controlled trial to determine the impact of the distribution of laptops to children on EGL outcomes. There are no major concerns about selection bias	The study uses a credible social network analysis to determine the spillover effects of the program	There are no significant outcome and analysis reporting biases	There is no evidence for significant other risks of bias
Campos et al. (2011)	*High risk of selection bias and confounding*	*Low risk of performance bias*	*High risk of outcome and analysis reporting bias*	*Medium risk of other biases*
	The study uses hierarchical regression analysis to determine the impact of participation in preschool on EGL outcomes. This methodology is not a well‐established method to account for selection bias	The analysis compares beneficiary schools with comparison schools that appear to be sufficiently isolated from the beneficiary schools	The study only reports statistically significant effects in the tables. However, the narrative suggests that not all results were statistically significant. This is an indication for outcome and analysis reporting bias	The study does not account for clustering in the estimation of the standard errors
Cardoso‐Martins et al. (2011), experiment 1	*Medium risk of selection bias*	*High risk of performance bias*	*Low risk of outcome and analysis reporting bias*	*Low risk of other biases*
	The study uses a randomized controlled trial to determine the impact of the program on EGL outcomes. However, the sample only consisted of 32 students. This sample size is not sufficient to ensure equivalence in observable and unobservable characteristics	The study used randomization at the student‐level within the same school. There is thus a lot of interaction between beneficiary and control students. This interaction creates a major risk of performance bias	There are no significant outcome and analysis reporting biases	There is no evidence for significant other risks of bias
Cardoso‐Martins et al. (2011), experiment 2	*Medium risk of selection bias*	*High risk of performance bias*	*Low risk of outcome and analysis reporting bias*	*Low risk of other biases*
	The study uses a randomized controlled trial to determine the impact of the program on EGL outcomes. However, the sample only consisted of 20 students. This sample size is not sufficient to ensure equivalence in observable and unobservable characteristics	The study used randomization at the student‐level within the same school. There is thus a lot of interaction between beneficiary and control students. This interaction creates a major risk of performance bias	There are no significant outcome and analysis reporting biases	There is no evidence for significant other risks of bias
Cristia et al. (2012)	*Low risk of selection bias*	*Low risk of performance bias*	*Low risk of outcome and analysis reporting bias*	*Low risk of other biases*
	The study uses a cluster‐randomized controlled trial to determine the impact of the distribution of laptops to children on EGL outcomes. There are no major concerns about selection bias	The analysis compares beneficiary schools with control schools that appear to be sufficiently isolated from the beneficiary schools	There are no significant outcome and analysis reporting biases	There is no evidence for significant other risks of bias
Felício et al. (2012)	*Medium risk of selection bias*	*Medium risk of performance bias*	*High risk of outcome and analysis reporting bias*	*Low risk of other biases*
	The study uses a propensity score matching design to assess the effects of participation in preschool on EGL outcomes. This design enables the researchers to correct for selection‐bias from observable characteristics. However, the selection bias is still medium because the methodology does not allow the researchers to account for unobservable characteristics from self‐selection into preschool	The study compares beneficiaries with nonbeneficiaries in the same municipality. Thus, there is a risk of interaction between beneficiary and comparison students, which we interpret as a medium risk of performance bias	The authors fail to report statistically insignificant effects. However, the narrative indicates that the results are not statistically significant in all specifications. This discrepancy in reporting indicates a high risk of outcome and analysis reporting bias	There is no evidence for significant other risks of bias
Ferrando et al. (2011)	*Medium risk of selection bias*	*Medium risk of performance bias*	*Medium risk of outcome and analysis reporting bias*	*Medium risk of other biases*
	The study uses a propensity score matching design to assess the effects of the distribution of laptops to children on EGL outcomes. This design enables the researchers to correct for selection‐bias from observable characteristics. However, the selection bias is still medium because the methodology does not allow the researchers to account for unobservable characteristics	The analysis compares beneficiary schools with comparison schools that appear to be sufficiently isolated from the beneficiary schools	The study only uses a subset of available control variables for the propensity score matching. It is unclear why the authors do not include the other potential control variables. This approach may be an indication for outcome and analysis reporting bias	The study does not account for clustering in the estimation of the standard errors
Gomez Franco (2014)	*High risk of selection bias*	*Low risk of performance bias*	*High risk of outcome and analysis reporting bias*	*High risk of other biases*
	The study uses a cluster‐randomized controlled trial to determine the impact of the program on EGL outcomes. However, the study analyses data for beneficiaries that comply with the instructions during the training. This nonrandom sample significantly increases the risk of selection bias. In addition, the authors use several potentially endogenous characteristics as control variables	The analysis compares beneficiary schools with control schools that appear to be sufficiently isolated from the beneficiary schools	The study uses several potentially endogenous characteristics in the estimation of the impact of the program. This approach is an indication for outcome and analysis reporting bias	The study does not account for clustering in the estimation of the standard errors
Ismail et al. (2014)	*Medium risk of selection bias*	*Medium risk of performance bias*	*Low risk of outcome and analysis reporting bias*	*Low risk of other biases*
	The study uses a propensity score matching design to assess the effects of a school feeding program on EGL outcomes. This design enables the researchers to correct for selection‐bias from observable characteristics. However, the selection bias is still medium because the methodology does not allow the researchers to account for unobservable characteristics	The analysis suggests that comparison schools may not be sufficiently isolated from the beneficiary schools. Thus, there is a medium risk of performance bias	There are no significant outcome and analysis reporting biases	There is no evidence for significant other risks of bias
Larraín et al. (2012), study 1	*High risk of selection bias*	*Medium risk of performance bias*	*Low outcome and analysis reporting bias*	*Low risk of other biases*
	The study randomly assigns two classrooms to the treatment group and two classrooms to the control group. This sample size is too small to ensure equivalence in observable and unobservable characteristics. In addition, the authors do not present evidence for equivalence in observable characteristics. Balance tables are not reported	The program is randomly assigned as the classroom level within the same school. Thus, there may be interaction between beneficiary students and control students, which may result in spillovers	There are no significant outcome and analysis reporting biases	There is no evidence for significant other risks of bias
Larraín et al. (2012), study 2	*High risk of selection bias*	*Medium risk of performance bias*	*Low risk of outcome and analysis reporting bias*	*Low risk of other biases*
	The study randomly assigns two classrooms to the treatment group and two classrooms to the control group. This sample size is too small to ensure equivalence in observable and unobservable characteristics. In addition, the authors do not present evidence for equivalence in observable characteristics. Balance tables are not reported	The program is randomly assigned as the classroom level within the same school. Thus, there may be interaction between beneficiary students and control students, which may result in spillovers	There are no significant outcome and analysis reporting biases	There is no evidence for significant other risks of bias
Lockheed et al. (2010)	*Medium risk of selection bias*	*High risk of performance bias*	*Low risk of outcome and analysis reporting bias*	*Medium risk of other biases*
	The study uses a propensity score matching design to determine the impact of a school governance program on EGL outcomes. This methodology enables the researchers to control for observable characteristics. However, the risk of selection bias remains medium because the design does not allow for controlling for unobservable characteristics	The study reports that the comparison schools also often received the program but does not account for this in the analysis	There are no significant outcome and analysis reporting biases	The study does not account for clustering in the estimation of the standard errors
Maluccio et al. (2009)	*Medium risk of selection bias*	*Low risk of performance bias*	*Low risk of outcome and analysis reporting bias*	*Low risk of other biases*
	The study uses a cluster‐randomized controlled to determine the impact of a nutrition program on EGL outcomes. However, the sample size is very small and does not ensure equivalence in observable characteristics between treatment and control villages. The authors account for this concern by showing descriptive statistics, but there is nonetheless a medium risk of selection bias	The study uses village‐level randomization to determine the impact of the program on EGL outcomes. The villages appear to be sufficiently isolated, which limits the potential for bias from spillovers or contamination	There are no significant outcome and analysis reporting biases	There is no evidence for significant other risks of bias
Mendive et al. (2016)	*High risk of selection bias*	*Low risk of performance bias*	*High risk of outcome and analysis reporting bias*	*Medium risk of other biases*
	The study uses hierarchical least squares regression analysis to determine the effect of the compliance with a teacher training program on EGL outcomes. Compliance is determined by self‐selection. The use of regression analysis does not enable controlling for this self‐selection. Thus, the risk of selection‐bias is high	The program is randomly assigned as the school‐level. This approach limits the interaction between beneficiary and control students, which reduces the risk of bias from spillovers or contamination	The authors use arbitrary thresholds for determining whether the program was implemented with sufficient adherence and dosage. In addition, the authors use OLS regression analysis as opposed to instrumental variable regression analysis. The authors should have used the randomization as an instrument for compliance in order to appropriately estimate the impact of compliance with the program	The use of videos to measure teacher behavior could have resulted in Hawthorne effects, which could bias the impact of the program
Neugebauer and Currie‐Rubin (2009)	*Medium risk of selection‐bias*	*High risk of performance bias*	*Medium risk of outcome and analysis reporting bias*	*Medium risk of other biases*
	The study uses random assignment, but the sample size is insufficient to ensure equivalence in observable characteristics between treatment and control students	The program uses random assignment at the individual level, which increases the risk of spillovers and contamination considerably	The authors only report results for one outcome variable but collected data for several other outcome variables. This is an indication of outcome and analysis reporting bias	The reporting of the results suggests that the study may be biased due to Hawthorne effects
Pallante and Kim (2013)	*Low risk of selection bias*	*Medium risk of performance bias*	*Low risk of outcome and analysis reporting bias*	*Low risk of other biases*
	The study uses a cluster‐randomized controlled trial to determine the impact of the program on EGL outcomes. The authors also present evidence for balance in observable characteristics across treatment and control conditions. Thus, the risk of selection bias is low	The study used random assignment at the classroom level. Thus, there may have been interaction between beneficiary and control students. This interaction results in a medium risk of bias from spillovers or contamination	There are no significant outcome and analysis reporting biases	There is no evidence for significant other risks of bias
Powell et al. (1998)	*Low risk of selection bias*	*High risk of performance bias*	*High risk of outcome and analysis reporting bias*	*Low risk of other biases*
	The study uses student‐level randomization to determine the impact of the program on EGL outcomes. The study also shows evidence for balance in observable characteristics. Thus, we consider this study as low risk of selection bias	The study compares beneficiary and control students within the same classroom. This approach significantly increases the risk of spillovers and contamination	The study reports statistically significant effects. However, our effect size calculations suggest that the results are not statistically significant	There is no evidence for significant other risks of bias
Simeon et al. (1995)	*Low risk of selection bias*	*High risk of performance bias*	*Low risk of outcome and analysis reporting bias*	*Low risk of other biases*
	The study uses student‐level randomization to determine the impact of the program on EGL outcomes. The study also shows evidence for balance in observable characteristics. Thus, we consider this study as low risk of selection bias	The study compares beneficiary and control students within the same classroom. This approach significantly increases the risk of spillovers and contamination	There are no significant outcome and analysis reporting biases	There is no evidence for significant other risks of bias
Tapia and Benítez (2013)	*High risk of selection bias*	*High risk of performance bias*	*Medium risk of outcome and analysis reporting bias*	*Medium risk of other biases*
	The study uses random assignment with a sample of 10 treatment and 10 control students. This sample size is way too low to ensure equivalence in observable characteristics between treatment and control students	The study compares beneficiary and control students within the same school. This approach significantly increases the risk of spillovers and contamination	The study reports statistically significant effects with a very small sample size. However, the authors do not report the results of the outcome equation. Instead, the results are presented through graphs. Thus, we consider this study as medium risk of outcome and analysis reporting bias	The study suggests that researchers were heavily involved in the data collection, which may have resulted in Hawthorne effects
Murad and Topping (2000)	*High risk of selection bias*	*High risk of performance bias*	*Medium risk of outcome and analysis reporting bias*	*Medium risk of other biases*
	The study uses random assignment but the sample size is too small to ensure equivalence in observable characteristics. In addition, treatment students were switched to the control group because they could not comply with the intervention. Together, these constraints result in a high risk of selection bias	The study compares beneficiary and control students within the same school. This approach significantly increases the risk of spillovers and contamination	The authors exclude outliers from their analysis for unclear reasons. The exclusion of these outliers may have affected the statistical significance of the impact estimates	The study suggests that researchers were heavily involved in the data collection, which may have resulted in Hawthorne effects
Vivas (1996), experiment 1	*Medium risk of selection bias*	*Low risk of performance bias*	*Medium risk of outcome and analysis reporting bias*	*Low risk of other biases*
	The study uses random assignment but the sample size is too small to ensure equivalence in observable characteristics	The program is randomly assigned as the school‐level. This approach limits the interaction between beneficiary and control students, which reduces the risk of bias from spillovers or contamination	The study estimates the impact of the program by comparing the median value of EGL outcomes between beneficiary and control students. This approach is unusual and may be an indication for outcome and analysis reporting bias	There is no evidence for significant other risks of bias
Vivas (1996), experiment 2	*Medium risk of selection bias*	*Low risk of performance bias*	*Medium risk of outcome and analysis reporting bias*	*Low risk of other biases*
	The study uses random assignment but the sample size is too small to ensure equivalence in observable characteristics	The program is randomly assigned as the school‐level. This approach limits the interaction between beneficiary and control students, which reduces the risk of bias from spillovers or contamination	The study estimates the impact of the program by comparing the median value of EGL outcomes between beneficiary and control students. This approach is unusual and may be an indication for outcome and analysis reporting bias	There is no evidence for significant other risks of bias
Yoshikawa et al. (2015)	*Low risk of selection bias*	*Low risk of performance bias*	*Low risk of outcome and analysis reporting bias*	*Medium risk of other biases*
	The study uses a cluster‐randomized controlled trial to determine the impact of the program on EGL outcomes. The authors also present evidence for balance in observable characteristics across treatment and control conditions. Thus, the risk of selection bias is low	The program is randomly assigned as the school‐level. This approach limits the interaction between beneficiary and control students, which reduces the risk of bias from spillovers or contamination	There are no significant outcome and analysis reporting biases	The use of videos to measure teacher behavior could have resulted in Hawthorne effects, which could bias the impact of the program

Abbreviations: EGL, early grade literacy; OLS, ordinary least squares.

**Table D3:** Quantitative nonintervention quality review protocol

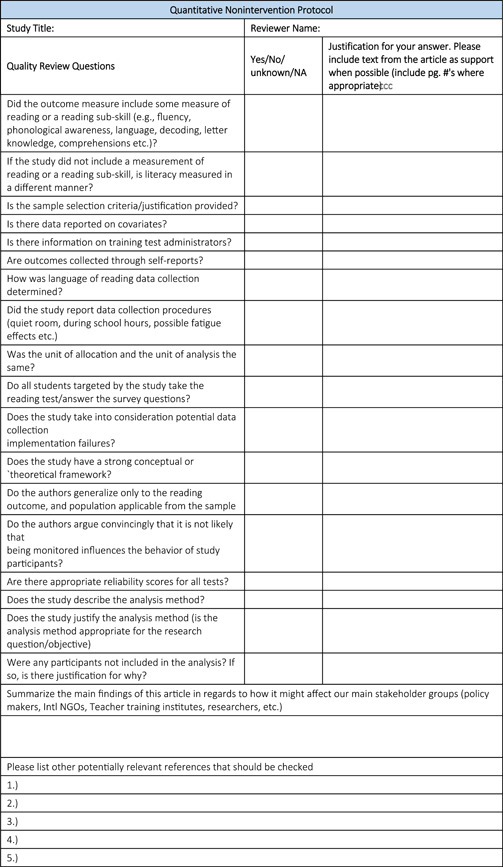

**Table D4:** Quality ratings for quantitative nonintervention studies

Studies	Theoretical framework explaining the study's motivation and findings	Quality of outcome measure	Sample selection quality	Quality of data collection procedures	Quality and relevance of analysis, given the research question	External validity
Guardia (2003)	High	High	Low	High	High	High
Bizama et al. (2011)	High	High	Low	Low	High	High
Muñoz (2011)	High	High	High	Low	High	Medium
Bandini et al. (2006)	Low	Medium	High	Low	Medium	Low
Barrera and Maluf (2003)	Low	High	High	Low	High	High
Cardoso‐Martins and Da Silva (2010)	Low	High	High	High	High	High
Cardoso‐Martins and Fulanete Correa (2008)	High	High	Medium	Medium	Medium	Medium
Cervini (2015)	High	Medium	High	Low	High	High
Giacomoni et al. (2015)	Low	Low	Low	Low	High	Low
Matute et al. (2012)	Low	Low	Medium	Medium	High	High
Torrecilla and Carrasco (2014)	High	Low	High	Low	High	High
De Abreu and Cardoso‐Martins (1998)	Low	High	High	Medium	High	Medium
Massone and Baez (2009)	Low	Medium	Low	Low	Low	Low
Dias et al. (2006)	Low	Medium	Low	Low	Low	Low
Páez et al. (2007)	High	High	High	High	Medium	Medium
Jaichenco and Wilson (2013)	High	High	High	High	High	Medium
Iparraguirre (2014)	High	Low	Low	Low	Low	Medium
Medeiros et al. (2011)	Medium	Medium	Low	Medium	Medium	Medium
Jiménez et al. (2009)	High	Medium	High	High	High	Medium
Gómez‐Pérez et al. (2011)	High	High	High	High	High	High
Athayde et al. (2014)	Low	Medium	Low	Low	High	Medium
Francis (1999)	Low	Medium	Low	Low	Medium	High
Salles and Parente (2002)	High	High	High	High	Medium	High
Goldenberg et al. (2014)	High	High	High	Low	High	Medium
Capovilla et al. (2004)	Low	Medium	Low	Low	Low	Low
Guevara et al. (2008)	High	High	High	High	High	Medium
Capovilla et al. (2004)	Medium	Medium	Low	Low	Low	Low
Benítez et al. (2007)	High	High	High	High	High	High
Silva et al. (2013)	High	High	High	Low	High	High
Moneda et al. (2009)	High	High	High	High	low	medium
Plana and Fumagalli (2013)	High	High	High	High	High	High
Fuller et al. (1999)	Low	Low	High	High	Low	Medium
Janus (2011)	Low	High	High	Low	High	Low
Cueto and Díaz (2013)	High	High	High	Low	High	Medium
Kim and Pallante (2012)	Low	High	High	Low	High	High
Bravo et al. (2002)	High	High	High	High	High	High
Favila et al. (1999)	High	High	High	Medium	High	Medium
Pino and Bravo (2005)	Medium	High	High	High	High	High
Querejeta et al. (2013)	High	High	High	High	High	Medium
de Manrique and Signorini (1994)	Low	High	Medium	Medium	High	Medium
Kudo and Bazan (2009)	Medium	Medium	High	Medium	High	High
Melchiori et al. (2012)	Low	Medium	Low	Low	Low	Medium
Abadzi et al. (2005)	Low	Medium	Low	Low	Low	Low
Morales et al. (2013)	Low	Medium	Medium	Low	Low	Medium
Reigosa‐Crespo et al. (2013)	High	High	High	High	Medium	Low
Oliveira (1996)	Low	High	High	High	High	Medium
Castro et al. (2002)	Medium	High	Medium	Medium	Medium	High
Ramírez et al. (2000)	High	High	Low	High	High	Low
Reynoso‐Alcántara et al. (2010)	High	High	High	High	High	Medium
Salazar et al. (1996)	High	High	High	High	High	Medium
Rosas et al. (2015)	High	High	High	Low	High	High
Salazar‐Reyes and Vega‐Pérez (2013)R	High	High	High	High	High	Medium
Rindermann et al. (2014)	Low	Low	Low	Low	Medium	High
Silva et al. (2014)	Low	Low	High	High	High	High
Reigosa‐Crespo et al. (2013)	High	High	High	Medium	High	Medium
Rego (1997)	Medium	High	High	Low	Medium	Medium
Treiman et al. (2006)	High	Medium	Medium	High	Medium	Medium
Kessler et al. (2013)	High	High	Medium	High	High	High
Correa and Dockrell (2007)	High	High	Medium	Medium	Medium	Low
Villalon and San Francisco (2001)	High	High	Medium	High	Medium	High
Hoddinott et al. (2013)	Medium	High	Medium	Medium	High	High

**Table D5:** Qualitative intervention and nonintervention quality review protocol **Directions:** Please list the title of the article and your name as reviewer in the appropriate rows. After reading the article, please rate each criteria as either high, medium, low or unclear by placing an “X” in the appropriate box. For any of the quality criteria that do not apply to the research in question, please place an “X” under the NA column. If you are unable to rate a particular criteria for low, medium or high levels of evidence because none is provided, then please place an “X” in the Not mentioned column. Whenever possible, provide the justification for your choices in the final column listing both strengths and weaknesses and supplying quotes from the article with page numbers

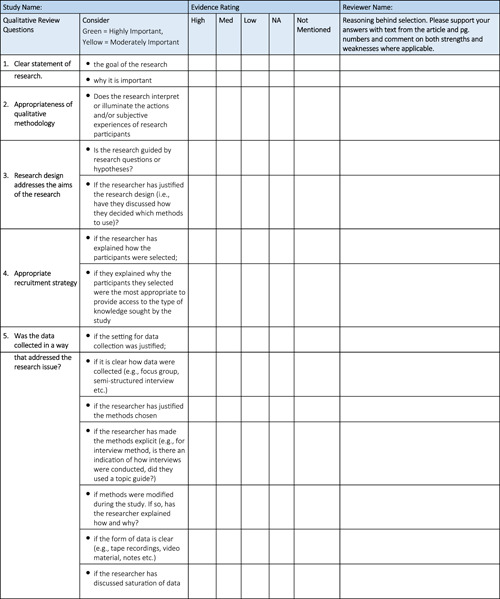

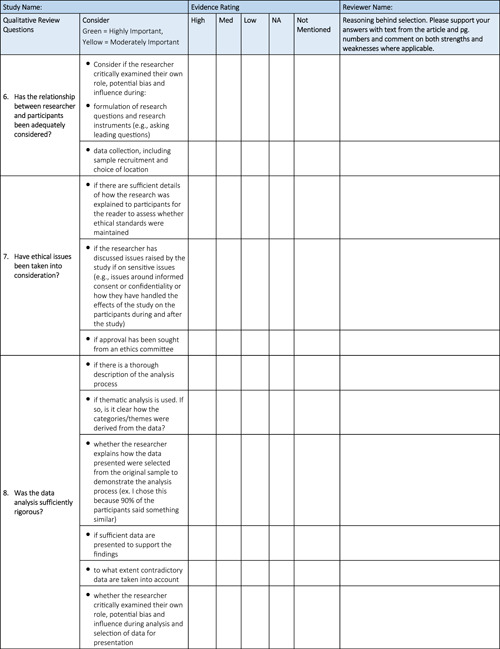

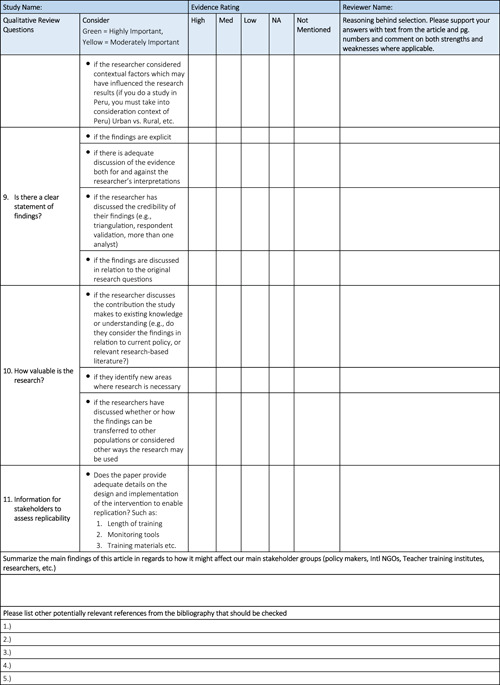

*Note:* High, level of evidence provided is strong; Low, the evidence provided is weak; Medium, level of evidence provided is adequate but not sufficient; NA, the criteria is not applicable to this research; Not Mentioned, no evidence is provided for the criteria.

## APPENDIX E QUALITATIVE REVIEW CRITERIA

**Table E1:** Qualitative review questions 1–2

Author (year)	(1) Clear statement of research		(2) Appropriateness of qualitative methodology
	The goal of the research	Why it is important	Does the research interpret or illuminate the actions and/or subjective experiences of research participants
Diuk (2007)	High	High	High
Gómez Nashiki (2008)	Medium	Not mentioned	Medium
Guevara and Ordoñez (2012)	High	High	High
Kinkhead‐Clark (2014)	High	High	High
Leal Carretero and Suro Sánchez (2012)	Medium	Medium	Not mentioned
Mahurt (1993)	High	High	High
Manrique and Borzone (2010)	High	High	High
Massone and Baez (2009)	High	High	High
Medina and Costa (2013)	High	Medium	High
Neugebauer and Currie‐Rubin (2009)	High	High	High
Porras González (2010)	Low	Unclear	Medium
Ribeiro and Souza (2012)	High	High	Unclear
Roofe (2014)	High	High	Medium
Rosado and Campelo (2011)	High	High	High
Jiménez et al. (2003)	High	High	High
Stein and Rosemberg (2012)	High	High	Not mentioned
Caldera de Briceño et al. (2010)	Low	Medium	Medium
Villamil and Vargas (2010)	Medium	N/A	N/A
Volk and De Acosta (2001)	High	High	High
Volk and De Acosta (2003)	Medium	Medium	High
Warrican et al. (2008)	High	High	High
Webster (2009)	High	Medium	High
González et al. (2013)	High	Medium	High

**Table E2:** Qualitative review questions 3–4

	(3) Research design addresses the aims of the research		(4) Appropriate recruitment strategy	
Author (year)	Is the research guided by research questions or hypotheses?	If the researcher has justified the research design (i.e., have they discussed how they decided which methods to use)?	If the researcher has explained how the participants were selected	If they explained why the participants, they selected were the most appropriate to provide access to the type of knowledge sought by the study
Belintane (2010)	Not mentioned	Low	High	Low
Castanheira et al. (2013)	Unclear	Unclear	Unclear	Unclear
Diuk (2007)	High	High	High	High
Gómez Nashiki (2008)	Not Mentioned	Medium	Medium	Not Mentioned
Guevara and Ordoñez (2012)	High	Not Mentioned	High	High
Kinkhead‐Clark (2014)	Medium	Medium	Low	Not mentioned
Leal Carretero and Suro Sánchez (2012)	Not mentioned	Medium	Not mentioned	Not mentioned
Mahurt (1993)	High	High	High	High
Manrique and Borzone (2010)	Not mentioned	low	High	Not mentioned
Massone and Baez (2009)	High	High	High	High
Medina and Costa (2013)	High	High	Not mentioned	Low
Neugebauer and Currie‐Rubin (2009)	High	High	High	High
Porras González (2010)	Low	Low	Unclear	Unclear
Ribeiro and Souza (2012)	Medium	Low	Low	Low
Roofe (2014)	Low	Low	Medium	Low
Rosado and Campelo (2011)	High	Low	Low	Low
Jiménez et al. (2003)	High	High	High	High
Stein and Rosemberg (2012)	Not mentioned	High	Not mentioned	Not mentioned
Caldera de Briceño et al. (2010)	Unclear	Low	Low	Low
Villamil and Vargas (2010)	High	Low	Medium	Low
Volk and De Acosta (2001)	High	High	High	High
Volk and De Acosta (2003)	Medium	Medium	Medium	High
Warrican et al. (2008)	Low	Not mentioned	High	High
Webster (2009)	High	High	Not mentioned	Not mentioned
González et al. (2013)	Medium	Medium	High	High

**Table E3:** Qualitative review question 5

	(5) Was the data collected in a way that addressed the research issue?						
Author (year)	If the setting for data collection was justified	If it is clear how data were collected (e.g., focus group, semistructured interview etc.)	If the researcher has justified the methods chosen	If the researcher has made the methods explicit (e.g., for interview method, is there an indication of how interviews were conducted, did they used a topic guide?)	If methods were modified during the study. If so, has the researcher explained how and why?	If the form of data is clear (e.g., tape recordings, video material, notes etc.)	If the researcher has discussed saturation of data
Belintane (2010)	Low	Medium	Medium	Medium	Medium	High	Not Mentioned
Castanheira et al. (2013)	High	High	Unclear	Unclear	Unclear	Low	N/a
Diuk (2007)	High	High	High	High	Not mentioned	High	Not mentioned
Gómez Nashiki (2008)	Low	Medium	Medium	Low	Not mentioned	Not mentioned	Not mentioned
Guevara and Ordoñez (2012)	High	High	Not mentioned	High	Not mentioned	High	Not mentioned
Kinkhead‐Clark (2014)	Medium	High	High	Not mentioned	Not mentioned	High	Not mentioned
Leal Carretero and Suro Sánchez (2012)	Not mentioned	Medium	Medium	Medium	Not mentioned	Medium	Not mentioned
Mahurt (1993)	Low	High	High	Low	N/A	High	N/A
Manrique and Borzone (2010)	Medium	Medium	Medium	Low	Not mentioned	High	Not mentioned
Massone and Baez (2009)	High	High	High	High	Unclear	High	Low
Medina and Costa (2013)	High	High	Medium	Not mentioned	Not mentioned	High	Not mentioned
Neugebauer and Currie‐Rubin (2009)	High	High	High	Medium	N/A	Low	N/A
Porras González (2010)	Medium	Low	Low	Low	Unclear	Medium	Unclear
Ribeiro and Souza (2012)	High	Low	Low	Low	Unclear	Unclear	N/A
Roofe (2014)	Low	High	Low	Low	Low	Low	Low
Rosado and Campelo (2011)	Low	Low	Low	Low	Unclear	Unclear	Unclear
Jiménez et al. (2003)	High	High	High	High	N/A	High	N/A
Stein and Rosemberg (2012)	High	High	Low	Not mentioned	Not mentioned	High	Not mentioned
Caldera de Briceño et al. (2010)	Low	Medium	Low	Low	Low	Medium	Low
Villamil and Vargas (2010)	Low	High	Low	Low	Not mentioned	Medium	Not mentioned
Volk and De Acosta (2001)	High	High	High	High	Not mentioned	High	Not mentioned
Volk and De Acosta (2003)	High	High	N/A	Medium	N/A	High	
Warrican et al. (2008)	Medium	High	Medium	High	Not Mentioned	High	Not mentioned
Webster (2009)	Not mentioned	High	High	Medium	Not mentioned	High	Not mentioned
González et al. (2013)	High	High	Low	NA	High	Low	Low

**Table E4:** Qualitative review questions 6–7

Author (year)	(6) Has the relationship between researcher and participants been adequately considered?		(7) Have ethical issues been taken into consideration?		
	Consider if the researcher critically examined their own role, potential bias, and influence during formulation of research questions and research instruments (e.g., asking leading questions)	Consider if the researcher critically examined their own role, potential bias, and influence during data collection, including sample recruitment and choice of location	If there are sufficient details of how the research was explained to participants for the reader to assess whether ethical standards were maintained	If the researcher has discussed issues raised by the study if on sensitive issues (e.g., issues around informed consent or confidentiality or how they have handled the effects of the study on the participants during and after the study)	If approval has been sought from an ethics committee
Diuk (2007)	Not mentioned	Low	Not mentioned	Not mentioned	Not mentioned
Gómez Nashiki (2008)	Not Mentioned	Low	Not mentioned	Not mentioned	Not mentioned
Guevara and Ordoñez (2012)	High	Not Mentioned	Not mentioned	Not mentioned	Not mentioned
Kinkhead‐Clark (2014)	High	High	Not mentioned	Not mentioned	Not mentioned
Leal Carretero and Suro Sánchez (2012)	Not mentioned	Low	Not mentioned	Not mentioned	Not mentioned
Mahurt (1993)	High	Not mentioned	Not mentioned	Not mentioned	Not mentioned
Manrique and Borzone (2010)	Not mentioned	low	Not mentioned	Not mentioned	Not mentioned
Massone and Baez (2009)	High	Medium	N/A	N/A	Not mentioned
Medina and Costa (2013)	High	High	Not mentioned	Not mentioned	Not mentioned
Neugebauer and Currie‐Rubin (2009)	Low	Low	Low	Low	Low
Ribeiro and Souza (2012)	N/A	N/A	Low	Low	N/A
Roofe (2014)	Not mentioned	Low			
Rosado and Campelo (2011)	High	Low			
Jiménez et al. (2003)	High	High			
Stein and Rosemberg (2012)	Not mentioned	Low	Low	Not mentioned	Not mentioned
Volk and De Acosta (2001)	Medium	High	Not mentioned	Not mentioned	Not mentioned
Volk and De Acosta (2003)	N/A	N/A	Medium	N/A	N/A
Warrican et al. (2008)	Not mentioned	Not mentioned	Not mentioned	Not mentioned	Not mentioned
Webster (2009)	High	Low	Not mentioned	Not mentioned	Not mentioned
González et al. (2013)	Low	Low	Low	Low	Low

**Table E5:** Qualitative review questions 8

Authors (year)	(8) Was the data analysis sufficiently rigorous?						
	If there is a thorough description of the analysis process	If thematic analysis is used. If so, is it clear how the categories/themes were derived from the data?	Whether the researcher explains how the data presented were selected from the original sample to demonstrate the analysis process (e.g., We chose this because 90% of the participants said something similar)	If sufficient data are presented to support the findings	To what extent contradictory data are taken into account	Whether the researcher critically examined their own role, potential bias, and influence during analysis and selection of data for presentation	If the researcher considered contextual factors which may have influenced the research results (if you do a study in Peru, you must take into consideration context of Peru) Urban vs. Rural, and so forth
Diuk (2007)	Medium	Low	Not mentioned	Low	Not mentioned	Not mentioned	Not mentioned
Gómez Nashiki (2008)	Low	Low	Not mentioned	Medium	Not mentioned	Not mentioned	Low
Guevara and Ordoñez (2012)	Medium	Not Mentioned	Not mentioned	High	Not mentioned	High	High
Kinkhead‐Clark (2014)	Not mentioned	High	Not mentioned	Low	Not mentioned	High	High
Leal Carretero and Suro Sánchez (2012)	Medium	Low	Medium	Medium	Low	Not mentioned	Not mentioned
Mahurt (1993)	High	Not mentioned	Not mentioned	High	High	Not mentioned	High
Manrique and Borzone (2010)	High	Medium	Not mentioned	Medium	Not mentioned	Not mentioned	Medium
Massone and Baez (2009)	High	High	High	Medium	Medium	High	High
Medina and Costa (2013)	Medium	Medium	Not mentioned	High	Not mentioned	High	High
Neugebauer and Currie‐Rubin (2009)	Low	N/A	High	High	High	Medium	High
Ribeiro and Souza (2012)	Low	Medium	N/A	Medium	N/A	N/A	Medium
Roofe (2014)	Medium	Medium	Low	Not mentioned	Not mentioned	Not mentioned	Low
Rosado and Campelo (2011)	Low	Not mentioned	Not mentioned	Low	N/A	Low	Low
Jiménez et al. (2003)	High	High	High	High	Not mentioned	Not mentioned	High
Stein and Rosemberg (2012)	High	Not mentioned	Low	Not mentioned	Not mentioned		
Volk and De Acosta (2001)	Medium	Low	Not mentioned	High	Not mentioned	Low	High
Volk and De Acosta (2003)	Medium	High	N/A	N/A	N/A	N/A	N/A
Warrican et al. (2008)	Low	N/A	Not mentioned	High	Not mentioned	Not mentioned	High
Webster (2009)	High	High	Not mentioned	High	Not mentioned	Not mentioned	High
González et al. (2013)	Low	Medium	Low	Medium	Low	Low	Low

**Table E6:** Qualitative review questions 9–11

Authors (year)	(9) Is there a clear statement of findings?				(10) How valuable is the research?			(11) Information for stakeholders to assess replicability
	If the findings are explicit	If there is adequate discussion of the evidence both for and against the researcher's interpretations	If the researcher has discussed the credibility of their findings (e.g., triangulation, respondent validation, more than one analyst)	If the findings are discussed in relation to the original research questions	If the researcher discusses the contribution the study makes to existing knowledge or understanding	If they identify new areas where research is necessary	If the researchers have discussed whether or how the findings can be transferred to other populations or considered other ways the research may be used	Does the paper provide adequate details on the design and implementation of the intervention to enable replication? Such as length of training, monitoring tools, training materials, and so forth
Diuk (2007)	High	Medium	Not mentioned	Low	High	High	Not mentioned	Low
Gómez Nashiki (2008)	Medium	Not mentioned	Not mentioned	Not mentioned	Medium	Medium	Not mentioned	N/A
Guevara and Ordoñez (2012)	High	Not Mentioned	Not mentioned	High	High	Not mentioned	High	Not mentioned
Kinkhead‐Clark (2014)	High	Not mentioned	Not mentioned	Medium	High	Not mentioned	Not mentioned	Not mentioned
Leal Carretero and Suro Sánchez (2012)	Medium	Not mentioned	Not mentioned	Low	Medium	N/A	Not mentioned	Not mentioned
Mahurt (1993)	High	Medium	High	High	Not mentioned	High	Not mentioned	Not mentioned
Manrique and Borzone (2010)	Medium	Medium	Medium	Medium	Low	Low	Low	Not mentioned
Massone and Baez (2009)	High	High	High	High	High	High	High	High
Medina and Costa (2013)	Medium	Not mentioned	Medium	High	High	High	Low	Not mentioned
Neugebauer and Currie‐Rubin (2009)	High	High	High	High	High	Medium	High	High
Ribeiro and Souza (2012)	Medium	Low	Low	N/A	Low	Low	Low	N/A
Roofe (2014)	High	Low	Low	Medium	Medium	Low	Low	Low
Rosado and Campelo (2011)	N/A	Low	Low	Medium	N/A	N/A	N/A	N/A
Jiménez et al. (2003)	High	Medium	High	High	High	N/A	High	N/A
Stein and Rosemberg (2012)	High	Not mentioned	Not mentioned	Not mentioned	Low	Not mentioned	High	Not mentioned
Volk and De Acosta (2001)	High	Not mentioned	Low	High	High	Not mentioned	High	Not mentioned
Volk and De Acosta (2003)	Medium	N/A	N/A	N/A	N/A	N/A	N/A	N/A
Warrican et al. (2008)	High	Not mentioned	Not mentioned	N/A	High	Not mentioned	Not mentioned	Not mentioned
Webster (2009)	High	Not mentioned	High	High	High	Not mentioned	Medium	Not mentioned
González et al. (2013)	Medium	Low	Low	High	Medium	Low	Low	Medium

## APPENDIX F ARTICLES REJECTED—BREAKDOWN BY INCLUSION CRITERIA

**Table:**

	Yes	No	Unclear	Unrated (because other criteria are not met)
Published after 1990?	1,138	5	5	
Study on the LAC region?	458	500	6	124
Boys or girls birth through grade 3?	248	215	17	668
Focus on reading or literacy?	186	134	1	827
Is it research?	166	60	0	922
Does the research meet minimum criteria for the analysis?	124	42	0	982
